# Recombinant monoclonal antibody siltartoxatug versus plasma-derived human tetanus immunoglobulin for tetanus: a randomized, double-blind, active-controlled, phase 3 trial

**DOI:** 10.1038/s41591-025-03791-8

**Published:** 2025-07-08

**Authors:** Zijing Liang, Si Liu, Wei Guo, Zhe Deng, Wenkai Bin, Anyong Yu, Junyan Hu, Lidong Wu, Zhanfei Li, Wei Huang, He Li, Dapeng Cheng, Shugui Li, Qinghao Guo, Dongshan Zhang, Xinming Yan, Chunlei Wang, Wenwei Cai, Banghan Ding, Wenqiang Li, Xu Li, Bin Xu, Lei He, Yanhong Ouyang, Hong Zhan, Jianwei Wang, Yan Zhao, Xinyu Liu, Wenxi Xiang, Meizhuo Zhang, Zhihua Zhang, Jiyuan Ding, Xiaohu Kuang, Weihong Zheng, Huaxin Liao, Wanmei Wang, Chuanlin Wang

**Affiliations:** 1 Department of Emergency, The First Affiliated Hospital of Guangzhou Medical University, Guangzhou, China; 2 Department of Emergency, Peking University First Hospital, Beijing, China; 3 Department of Emergency Medicine, The First Affiliated Hospital of Shenzhen University, Shenzhen Second People’s Hospital, Shenzhen, China; 4 Department of Emergency, The Affiliated Nanhua Hospital, Hengyang Medical School, University of South China, Hengyang, China; 5 Department of Emergency, Affiliated Hospital of Zunyi Medical University, Zunyi, China; 6 Department of Emergency, The Third Affiliated Hospital of Guangzhou Medical University, Guangzhou, China; 7 Department of Emergency, The Second Affiliated Hospital of Nanchang University, Nanchang, China; 8 Department of Traumatic Surgery, Tongji Hospital of Tongji Medical College, Huazhong University of Science and Technology, Wuhan, China; 9 Department of Emergency, Liuzhou Worker’s Hospital, Liuzhou, China; 10 Department of Emergency Surgery, The Second Affiliated Hospital of Anhui Medical University, Hefei, China; 11 Department of Emergency, Yuncheng Central Hospital, Yuncheng, China; 12 Department of Emergency, The First People’s Hospital of Jinzhong, Jinzhong, China; 13 Trauma Department, The Central Hospital of Wuhan (Affiliated to Tongji Medical College, Huazhong University of Science and Technology), Wuhan, China; 14 Department of Emergency, The Second Xiangya Hospital of Central South University, Changsha, China; 15 Department of Emergency, Shanxi Bethune Hospital, Shanxi Academy of Medical Sciences, Third Hospital of Shanxi Medical University, Taiyuan, China; 16 Department of Burn Skin Surgery, PKUcare Luzhong Hospital, Zibo, China; 17 Department of Emergency, Zhejiang Provincial People’s Hospital, Hangzhou, China; 18 Department of Emergency, The Second Affiliated Hospital of Guangzhou University of Chinese Medicine, Guangdong Provincial Hospital of Chinese Medicine, Guangzhou, China; 19 Department of Emergency, Renmin Hospital of Wuhan University, Wuhan, China; 20 Department of Emergency, Nanfang Hospital, Southern Medical University, Guangzhou, China; 21 Department of Emergency, Beijing Tiantan Hospital, Capital Medical University, Beijing, China; 22 Department of Gastrointestinal Surgery, Hefei First People’s Hospital, Hefei, China; 23 Department of Emergency, Hainan General Hospital (Hainan Affiliated Hospital of Hainan Medical University), Haikou, China; 24 Department of Emergency, The First Affiliated Hospital, Sun Yat-sen University, Guangzhou, China; 25 Department of Orthopaedic Surgery, Guangzhou First People’s Hospital, Guangzhou, China; 26 Emergency Center, Zhongnan Hospital of Wuhan University, Wuhan, China; 27 Zhuhai Trinomab Pharmaceutical Co., Ltd, Zhuhai, China; 28 Department of Emergency Surgery, Peking University People’s Hospital, Beijing, China

**Keywords:** Drug development, Bacterial infection, Antibody therapy

## Abstract

Tetanus remains an important global public health concern. Currently, the only recommended passive immunization therapy for tetanus prophylaxis is plasma-derived human tetanus immunoglobulin (HTIG), which faces a global supply shortage and can transmit infectious pathogens. Despite not being endorsed by WHO due to safety concerns, equine tetanus antitoxin remains widely used in some countries. We conducted a randomized, double-blind, phase 3 trial to evaluate siltartoxatug—a first-in-class recombinant monoclonal antibody—for tetanus postexposure prophylaxis. Participants (*n* = 675) were randomized (2:1) to receive a single intramuscular injection of siltartoxatug 10 mg or HTIG 250 IU. The study met its primary outcome, with siltartoxatug demonstrating superiority to HTIG in the proportion of participants with an increase of anti-tetanus neutralizing antibody titers from baseline (ΔTiter) ≥ 0.01 IU ml^−^^1^ (95.4% versus 53.2%; intergroup difference 42.3% (95% confidence interval, 35.5–49.1; *P* < 0.0001)). The safety profiles were comparable, with similar incidence of adverse events between the siltartoxatug (38.2%, 168 of 440) and HTIG (33.9%, 75 of 221) groups. These findings highlight siltartoxatug as an effective and safe option for passive immunization against tetanus. ClinicalTrials.gov registration: NCT05664750.

## Abstract (web summary)

Siltartoxatug, a first-in-class recombinant monoclonal antibody for tetanus prophylaxis, was shown to be safe and superior to plasma-derived human tetanus immunoglobulin in achieving anti-tetanus neutralizing antibody titers 12 hours after administration.

## Main

Tetanus is a life-threatening infectious disease caused by a potent neurotoxin produced by the bacterium *Clostridium tetani*. It remains an important public health concern in many parts of the world, particularly in low-income countries or regions with suboptimal vaccination programs.

Globally, an estimated 34,684 deaths were attributable to tetanus in 2019. Most new cases of tetanus occur in South Asia and sub-Saharan Africa^1^. Although tetanus is rare in developed countries due to robust childhood immunization programs, certain groups remain at risk for clinical tetanus, including the elderly, immunocompromised people, immigrants with unclear vaccination histories and unvaccinated children^2^.

Vaccination coverage for tetanus is suboptimal worldwide. In 2022, 14.3 million infants did not receive the initial dose of the diphtheria-tetanus-pertussis (DTP) vaccine, and an additional 6.2 million were only partially vaccinated^3^. Even in Europe, a seroprevalence study has shown that 2–31% of adults lack the protective level of anti-tetanus antibodies^4^.

Active immunization with the vaccine may take weeks to take full effect, whereas the incubation period of tetanus is usually short after exposure, with a median of 7 days^5^. Therefore, patients who lack adequate immunity and suffer from tetanus-prone wounds require passive immunization to provide immediate protection with a ‘protective’ serum level of antibodies.

The World Health Organization (WHO) recommends that passive immunization therapy be readily available in all countries for tetanus prophylaxis and treatment^5^. The administration of equine tetanus antitoxin for passive immunization after injury became common in World War I^6^. However, it is frequently associated with adverse reactions, with allergic reactions occurring in 5–30% of cases^7,8^. Fatal anaphylaxis occurs in approximately 0.001% of cases^9^. In addition, the half-life of equine antitoxin in humans is short, providing passive immunity for only about 2 weeks^10,11^. It has been replaced by human tetanus immunoglobulin (HTIG) in developed countries and was removed from the WHO’s list of essential medicines in 1991 (ref. ^12^). However, equine tetanus antitoxin remains widely used in developing countries due to the shortage and high cost of HTIG^13,14^.

HTIG—prepared from the plasma of healthy donors hyperimmunized with tetanus toxoid—was introduced in the early 1960s^15^. Compared to equine tetanus antitoxin, HTIG has a superior safety profile and a circulating half-life of 28 days^16^, making it a preferable option. It is currently the only passive immunizing agent for tetanus in most developed countries. However, the shortage of human plasma and plasma-derived medical products, including HTIG, has emerged as a pressing concern globally^17^. Low- and middle-income countries face critical long-term supply deficiencies due to insufficient domestic plasma supplies and a lack of technical and financial capacity for plasma fractionation^18^. Even in developed countries, achieving self-sufficiency in plasma is challenging. Other concerns of HTIG include the risk of transmitting infectious pathogens, severe allergic reactions, potential immune reactions in IgA-deficient patients and lot-to-lot variability^19,20^. Therefore, there is an urgent need to develop a safe, highly effective and more accessible alternative to plasma-derived HTIG and equine tetanus antitoxin for tetanus prevention and treatment.

Monoclonal antibodies (mAbs), which can be produced at a large scale through a standardized industrial process, have emerged as alternatives to plasma-derived human immunoglobulin (Ig) for combating several infectious diseases. Successful examples include mAbs targeting respiratory syncytial virus (RSV) and rabies^21^. Recent publications have highlighted the development of mAbs against tetanus as an alternative to substitute TAT and HTIG, underscoring growing interest in this approach^22–27^, which is also endorsed by the WHO^28^.

Siltartoxatug (formerly TNM002 or TT069) is the first tetanus-specific mAb^29^. Its active ingredient is a recombinant native human IgG1 mAb targeting tetanus toxin, produced in Chinese hamster ovary cells. The formulation contains excipients, including histidine, histidine hydrochloride, sodium chloride, sucrose, polysorbate 80 and water for injection. Siltartoxatug neutralizes tetanus toxin by specifically binding to key functional sites on the AB fragment of the toxin, thereby preventing the onset of tetanus^29^. In the completed phase 1 (dose range, 10–250 µg kg^−1^) and phase 2 clinical trials (dose range, 5–15 mg), primarily involving healthy volunteers, siltartoxatug demonstrated a favorable safety profile. Additionally, siltartoxatug provided high anti-tetanus neutralizing antibody titers within 12 h postadministration, with protective antibody levels sustained for several months (ClinicalTrials.gov registrations: NCT04629131, NCT05842798 and NCT05625477).

Here, we present findings from a confirmatory phase 3 clinical trial evaluating the efficacy and safety of siltartoxatug compared to those of HTIG for passive immunization against tetanus in participants with tetanus-prone wounds.

## Results

### Participant disposition and baseline characteristics

**Fig. 1:**
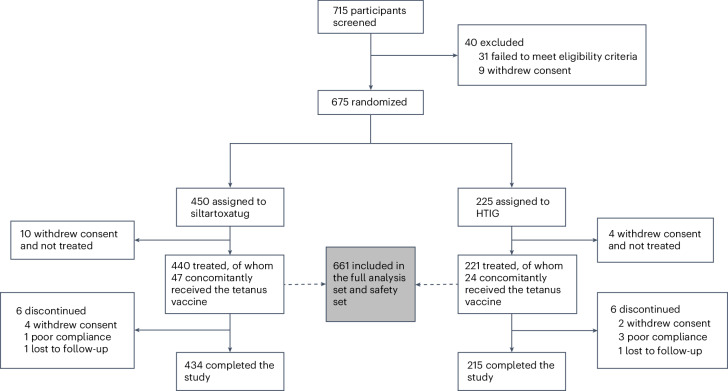
CONSORT flow diagram. The full analysis set included all randomized participants who received the study drug. The safety set included all treated participants with at least one postadministration safety evaluation. The full analysis set was used for efficacy analysis. The safety set was used for safety analysis.

Participants were recruited primarily from emergency departments in 28 hospitals across 14 provinces in China, between 22 December 2022 and 23 March 2023. A total of 715 candidate participants were assessed for eligibility, of whom 675 were enrolled; 661 received either siltartoxatug (*n* = 440) or HTIG (*n* = 221). Among the 661 participants, 71 (approximately 10% as anticipated) concomitantly received adsorbed tetanus vaccine. Most (60) received two doses of the vaccine, administered 4–8 weeks apart. A total of 649 participants completed the 105-day follow-up period. The reasons for discontinuation included withdrawal of consent (6), poor compliance (4) and loss to follow-up (2) (Fig. 1).

The median age at baseline was 43 years, with a range of 19–79 years. Participants presented to the clinical centers with unclean or contaminated wounds posing a risk for tetanus. The most common injuries were incised wounds (58.1%) and puncture wounds (19.4%).

Over half (58.2%) of the participants had baseline anti-tetanus antibody titers below the minimum protective level against tetanus (0.01 IU ml^−1^)^5,30^, while antibodies were completely undetectable in 40.7% of participants. This reflects insufficient previous tetanus immunization in the study participants, as well as in the general adult population in China^31,32^. It underscores the necessity of administering passive immunizing agents to provide rapid protection to those participants. Overall, baseline characteristics were similar between the treatment groups (Table 1).

**Table 1:** Baseline characteristics

	Siltartoxatug	HTIG	Total
	(*N* = 440)	(*N* = 221)	(*N* = 661)
**Age, years**	43.5 (19, 79)	41 (20, 76)	43 (19, 79)
**Male**	255 (58.0)	133 (60.2)	388 (58.7)
**Body mass index**	24.4 (16.7, 39.5)	24.0 (16.4, 38.9)	24.2 (16.4, 39.5)
**Baseline anti-tetanus antibody titers**			
Below the limit of quantitation	188 (42.7)	81 (36.7)	269 (40.7)
<0.01 IU ml^−1^	263 (59.8)	122 (55.2)	385 (58.2)
≥0.01 IU ml^−1^	176 (40.0)	98 (44.3)	274 (41.5)
GMT, IU ml^−1^	0.00729	0.00812	0.00756
**Type of injury**			
Incised wound	256 (58.2)	128 (57.9)	384 (58.1)
Puncture wound	87 (19.8)	41 (18.6)	128 (19.4)
Traffic-related injury	36 (8.2)	16 (7.2)	52 (7.9)
Animal bite or scratch	21 (4.8)	9 (4.1)	30 (4.5)
Burn	13 (3.0)	5 (2.3)	18 (2.7)
Other	27 (6.1)	22 (10.0)	49 (7.4)
**Wound classification**^**a**^			
Contaminated wounds	354 (80.5)	168 (76.0)	522 (79.0)
Unclean wounds	86 (19.5)	53 (24.0)	139 (21.0)
Clean wounds	0	0	0
**Wound site**			
Upper extremities	294 (66.8)	155 (70.1)	449 (67.9)
Lower extremities	117 (26.6)	52 (23.5)	169 (25.6)
Head and face	40 (9.1)	20 (9.0)	60 (9.1)
Chest	3 (0.7)	1 (0.5)	4 (0.6)
Back	1 (0.2)	0	1 (0.2)
Abdomen	1 (0.2)	0	1 (0.2)
Perineum	1 (0.2)	1 (0.5)	2 (0.3)
Other	6 (1.4)	1 (0.5)	7 (1.1)

Each wound site was counted once if a participant had more than one wound site.

Data are presented as *n* (%) or median (min, max) unless otherwise specified.

^a^Definitions for different wound classification: contaminated wounds are wounds contaminated by dirt, organic soil (for example, swamp or jungle soil), feces or saliva (for example, animal or human bites), infected wounds or wounds containing necrotic tissue (for example, necrosis or gangrene), gunshot wounds, frostbite, burns and so on. Unclean wounds are wounds located in areas of the body with high bacterial colonization (for example, armpits, groin and perineum) or simple wounds that have not been treated within 6 h of injury. Clean wounds are simple wounds located in areas of the body with minimal bacterial colonization, and treated promptly after injury (such as a cut from a blade).

### Primary outcome

To determine whether the participants developed protective serum antibody levels after receiving the study drug, the change in anti-tetanus neutralizing antibody titers from baseline (ΔTiter) was calculated. Regarding the primary outcome, the proportion of participants with ΔTiter ≥ 0.01 IU ml^−1^ 12 h postadministration were 95.4% and 53.2% in the siltartoxatug and HTIG groups, respectively. The difference between groups (42.3%; 95% confidence interval (CI), 35.5–49.1%; *P* < 0.0001) was statistically significant (Table 2).

**Table 2:** Summary of anti-tetanus neutralizing antibody ΔTiter level after study drug administration (primary and tertiary outcomes)

Timepoint	Participants with baseline titer and ΔTiter ≥ 0.01 IU ml^−1^ (%)				Geometric mean baseline titer and ΔTiter, IU ml^−1^			
	Siltartoxatug	HTIG	Difference	*P* value	Siltartoxatug	HTIG	Ratio	*P* value
	(*N* = 440)	(*N* = 221)	(95% CI)		(*N* = 440)	(*N* = 221)	(95% CI)	
**Baseline**	40.0%	44.3%	−4.45% (−12.5 to 3.49)	0.2743	0.00729 (313.8)	0.00812 (252.0)	0.92 (0.721–1.17)	0.5038
**12** **h**^**a**^	95.4%	53.2%	42.3% (35.5–49.1)	<0.0001	0.0602 (117.5)	0.0100 (85.0)	6.02 (5.08–7.12)	<0.0001
**3** **days**	99.7%	96.4%	3.35% (1.33–7.03)	0.0011	0.187 (78.9)	0.0332 (63.7)	5.54 (4.88–6.29)	<0.0001
**7** **days**	99.7%	97.4%	2.31% (0.595–5.63)	0.0092	0.233 (61.9)	0.0360 (50.5)	6.52 (5.85–7.27)	<0.0001
**28** **days**	99.7%	95.8%	3.91% (1.73–7.77)	0.0003	0.156 (43.9)	0.0208 (52.5)	7.13 (6.48–7.85)	<0.0001
**90** **days**	91.5%	10.1%	81.3% (75.5–85.8)	<0.0001	0.0253 (70.9)	0.00429 (75.0)	5.38 (4.68–6.18)	<0.0001

The baseline titer and the ΔTiter (increase of titer from baseline) at different timepoints are presented as the geometric mean (percentage of coefficient of variation).

ΔTiter values for the siltartoxatug group were calculated as the postadministration antibody titer minus the baseline antibody titer, measured using the TNM002 PD bioanalytical method. Similarly, ΔTiter values for the HTIG group were calculated based on antibody titers measured by the HTIG PD bioanalytical method.

^a^Primary outcome of the study was the proportion of participants with ΔTiter ≥ 0.01 IU ml^−1^ at 12 h postadministration. The tertiary outcome included the ΔTiter at 3, 7, 28 and 90 days postadministration.

For the tertiary efficacy endpoint analyses, participants who concomitantly received the tetanus vaccine in the siltartoxatug group (*n* = 47) and HTIG group (*n* = 24) were excluded to avoid interference.

The proportion of participants with ΔTiter ≥ 0.01 IU ml^−1^ was calculated, and the corresponding 95% CI was constructed using the Clopper–Pearson method. A 95% CI for intergroup difference was calculated using Miettinen–Nurminen method. A mixed-effect model for repeated measures was used to compare and analyze ΔTiters between groups at each scheduled timepoint. All tests were two-sided. No adjustments were made for multiple comparisons.

### Secondary outcome

The secondary objective of this study was to monitor tetanus occurrence following study drug administration. No cases of tetanus occurred during the study, indicating a 100% protection rate against tetanus in both groups throughout the study period (Extended Data Table 1).

### Safety

Adverse events (AEs) were collected throughout the study to evaluate the safety profile of the study drugs. The overall incidence of AEs was 38.2% (168 of 440) in the siltartoxatug group and 33.9% (75 of 221) in the HTIG group. The incidence of treatment-related AEs was also similar between the groups (4.8% in the siltartoxatug group and 3.6% in the HTIG group). The most common treatment-related AEs in the siltartoxatug group were transaminases increased and hematuria, each occurring in three (0.7%) participants. No participants experienced AEs that resulted in early withdrawal from the study (Table 3).

**Table 3:** Summary of AEs and adverse reactions (safety set)

	Siltartoxatug 10 mg	HTIG 250 IU	Total
	(*N* = 440)	(*N* = 221)	(*N* = 661)
	*n* (%)	*n* (%)	*n* (%)
**Any AE**	168 (38.2)	75 (33.9)	243 (36.8)
Mild	127 (28.9)	54 (24.4)	181 (27.4)
Moderate	69 (15.7)	29 (13.1)	98 (14.8)
Severe	5 (1.1)	2 (0.9)	7 (1.1)
**Serious AE**	6 (1.4)	2 (0.9)	8 (1.2)
**AEs leading to study discontinuation**	0	0	0
**Death**	0	1 (0.5)	1 (0.2)
**Treatment-related AE**	21 (4.8)	8 (3.6)	29 (4.4)
**Investigation**	9 (2.0)	2 (0.9)	11 (1.7)
Transaminases increased	3 (0.7)	0	3 (0.5)
Urinary occult blood positive	2 (0.5)	1 (0.5)	3 (0.5)
Blood glucose increased	2 (0.5)	0	2 (0.3)
Glucose urine present	2 (0.5)	0	2 (0.3)
Aspartate aminotransferase increased	1 (0.2)	1 (0.5)	2 (0.3)
Blood bilirubin increased	1 (0.2)	0	1 (0.2)
Electrocardiogram Q wave abnormal	1 (0.2)	0	1 (0.2)
Gamma-glutamyltransferase increased	1 (0.2)	0	1 (0.2)
Alanine aminotransferase increased	0	1 (0.5)	1 (0.2
**Renal and urinary disorders**	3 (0.7)	1 (0.5)	4 (0.6)
Hematuria	3 (0.7)	1 (0.5)	4 (0.6)
**General disorders and administration site conditions**	2 (0.5)	1 (0.5)	3 (0.5)
Injection site erythema	1 (0.2)	0	1 (0.2)
Injection site hypoesthesia	1 (0.2)	0	1 (0.2)
Injection site pain	1 (0.2)	0	1 (0.2)
Injection site swelling	0	1 (0.5)	1 (0.2)
**Nervous system disorders**	2 (0.5)	1 (0.5)	3 (0.5)
Dizziness	1 (0.2)	1 (0.5)	2 (0.3)
Head discomfort	1 (0.2)	0	1 (0.2)
**Blood and lymphatic system disorders**	2 (0.5)	0	2 (0.3)
Anemia	1 (0.2)	0	1 (0.2)
Coagulopathy	1 (0.2)	0	1 (0.2)
**Cardiac disorders**	2 (0.5)	0	2 (0.3)
Arteriospasm coronary	1 (0.2)	0	1 (0.2)
Ventricular extrasystoles	1 (0.2)	0	1 (0.2)
**Musculoskeletal and connective tissue disorders**	1 (0.2)	2 (0.9)	3 (0.5)
Arthralgia	1 (0.2)	0	1 (0.2)
Muscular weakness	0	1 (0.5)	1 (0.2)
Myalgia	0	1 (0.5)	1 (0.2)
**Skin and subcutaneous tissue disorders**	1 (0.2)	2 (0.9)	3 (0.5)
Allergic dermatitis	1 (0.2)	1 (0.5)	2 (0.3)
Urticaria	0	1 (0.5)	1 (0.2)
**Hepatobiliary disorders**	1 (0.2)	0	1 (0.2)
Abnormal hepatic function	1 (0.2)	0	1 (0.2)
**Metabolism and nutrition disorders**	1 (0.2)	0	1 (0.2)
Hyperuricemia	1 (0.2)	0	1 (0.2)

AEs were coded according to the System Organ Class and Preferred Term of the Medical Dictionary for Regulatory Activities terminology v.26.0.

A total of ten serious AEs (SAEs) were reported in eight participants, including seven SAEs in six (1.4%) participants from the siltartoxatug group and three SAEs in two (0.9%) participants from the HTIG group. All SAEs were considered unrelated to the study drug (Extended Data Table 2).

Most AEs were mild or moderate in severity. Five (1.1%) participants in the siltartoxatug group and two (0.9%) in the HTIG group reported severe AEs, including one fatal event with an unknown cause that occurred 3 months after receiving HTIG. All severe AEs were considered unrelated to the study drugs.

Injection site reactions, including erythema, hypoesthesia, pain and swelling, occurred in two participants in the siltartoxatug and one in the HTIG groups. These reactions were assessed as study-drug-related, mild in severity and resolved spontaneously within 1 week without medical intervention. Three treatment-related allergic reactions were reported, two in the HTIG group (a moderate urticaria and a mild allergic dermatitis) and a mild allergic dermatitis in the siltartoxatug group.

### Tertiary and other outcomes

At 3, 7 and 28 days postadministration, the proportion of participants with ∆Titer ≥ 0.01 IU ml^−1^ was consistently high (>95%) and comparable between the treatment groups. However, at 90 days postadministration, the proportion significantly decreased to 10.1% in the HTIG group, whereas it remained at 91.5% in the siltartoxatug group.

**Fig. 2:**
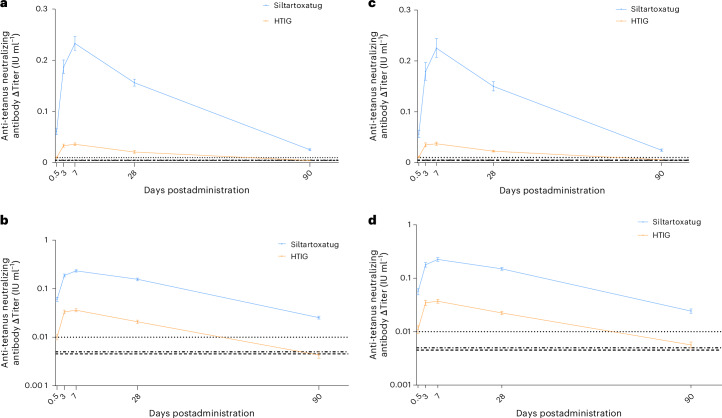
Anti-tetanus neutralizing antibody ΔTiter–time profiles. **a**,**b**, Anti-tetanus neutralizing antibody ΔTiter–time profiles are presented for the overall population (*n* = 393 for siltartoxatug, *n* = 197 for HTIG), with the *y* axis plotted using a linear (**a**) and a logarithmic (**b**) scale. **c**,**d**, Similarly, profiles for participants with baseline antibody titer <0.01 IU ml^−1^ (*n* = 263 for siltartoxatug, *n* = 122 for HTIG) are shown using a linear (**c**) and a logarithmic (**d**) scale. All participants included in the analysis had not received the tetanus vaccine concomitantly by the corresponding timepoints. The dotted line at 0.01 IU ml^−1^ represents the protective antibody threshold. The dashed line and dash-dotted line represent the lower limits of quantification for the bioanalytical methods used for HTIG (0.00453 IU ml^−1^) and siltartoxatug (0.005 IU ml^−1^), respectively. ΔTiter values are presented as the geometric mean; error bars, 95% CI.

The distribution of ∆Titer values in the two treatment groups is shown in Supplementary Fig. 1. The geometric mean ∆Titers of anti-tetanus neutralizing antibodies in the siltartoxatug group were higher than those in the HTIG group at all postadministration timepoints, with ratios (siltartoxatug/HTIG) ranging from 5.38 to 7.13 (Table 2). This demonstrated that, compared to HTIG, siltartoxatug provided higher level of antibodies for passive immunization against tetanus (Fig. 2).

In the analysis of participants with baseline antibody titers <0.01 IU ml^−1^—the most vulnerable subgroup—ΔTiters in both treatment groups aligned with the overall population. At each timepoint, the geometric mean ΔTiter in the siltartoxatug group remained significantly higher than that in the HTIG group (Fig. 2 and Extended Data Table 3).

**Fig. 3:**
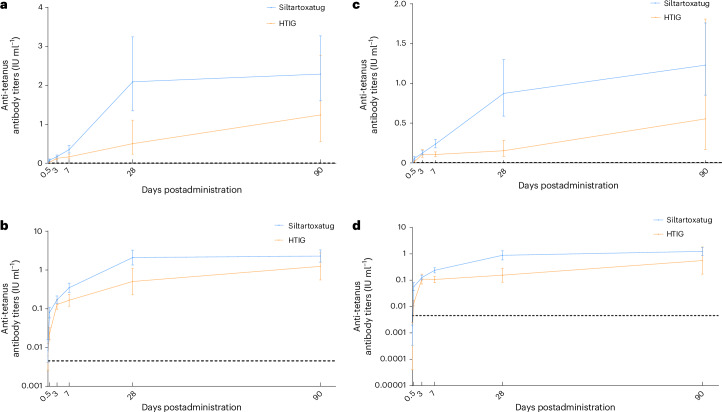
Anti-tetanus antibody titers–time profiles in the siltartoxatug + vaccine and HTIG + vaccine subgroups. **a**,**b**, Anti-tetanus antibody titers–time profiles are shown for the subgroups of participants who concomitantly received the tetanus vaccine (*n* = 47 for siltartoxatug, *n* = 24 for HTIG), with the *y* axis plotted using a linear (**a**) and a logarithmic (**b**) scale. **c**,**d**, Similarly, profiles for participants with baseline antibody titer <0.01 IU ml^−1^ in the subgroups (*n* = 28 for siltartoxatug, *n* = 14 for HTIG) are displayed on a linear (**c**) and a logarithmic (**d**) scale. Anti-tetanus antibody (polyclonal antibody) titers were measured using the HTIG PD bioanalytical method for both subgroups. The dashed line indicates the lower limit of quantification for the bioanalytical method (0.00453 IU ml^−1^). Titers are presented as the geometric mean; error bars, 95% CI.

For the 71 participants who concomitantly received adsorbed tetanus vaccines, the geometric mean titers (GMTs) of anti-tetanus antibodies (polyclonal antibodies induced by the vaccine) were consistently higher in the siltartoxatug+vaccine subgroup than in the HTIG + vaccine subgroup. At the critical timepoint for assessing the efficacy of tetanus vaccines—28 days postadministration—the GMTs of anti-tetanus antibodies were 2.09 (95% CI, 1.35–3.25) IU ml^−1^ in the siltartoxatug + vaccine subgroup, compared to 0.504 (95% CI, 0.230–1.10) IU ml^−1^ in the HTIG + vaccine subgroup. The proportions of participants with GMTs ≥0.1 IU ml^−1^ in the siltartoxatug + vaccine and HTIG + vaccine subgroups were 100% (95% CI, 92.5–100%) and 87.5% (95% CI, 67.6–97.3%), respectively (Extended Data Table 4). In participants with baseline antibody titers <0.01 IU ml^−1^ (*n* = 42), the GMTs of anti-tetanus antibodies were also consistently higher in the siltartoxatug + vaccine subgroup than in the HTIG + vaccine subgroup. At 28 days postadministration, the GMTs of anti-tetanus antibodies in the siltartoxatug + vaccine and HTIG + vaccine subgroups were 0.874 (95% CI, 0.587–1.30) IU ml^−1^ and 0.153 (95% CI, 0.0832–0.282) IU ml^−1^, respectively (Extended Data Table 5). Changes in anti-tetanus antibody titers over time in the siltartoxatug + vaccine and HTIG + vaccine subgroups are illustrated in Fig. 3.

The immunogenicity of siltartoxatug was evaluated among the 436 participants who received siltartoxatug and had immunogenicity data for at least one postadministration timepoint.

The incidence of anti-drug antibodies (ADA) was low, with 59 of 436 (13.5%) participants testing positive for ADAs, including one who was positive at baseline but negative postadministration. Additionally, 6 of 436 (1.4%) participants tested positive for neutralizing antibodies (NAb). NAbs developed in five participants by 90 days postadministration, whereas one participant exhibited NAbs as early as 28 days postadministration, which persisted through 90 days postadministration. This participant also showed the highest ADA titer (1,690) among all study participants. (Extended Data Table 6). A retrospective review showed that 72.4% of ADA-positive participants and all six NAb-positive participants received two doses of the tetanus vaccine.

**Extended Data Fig. 1:**
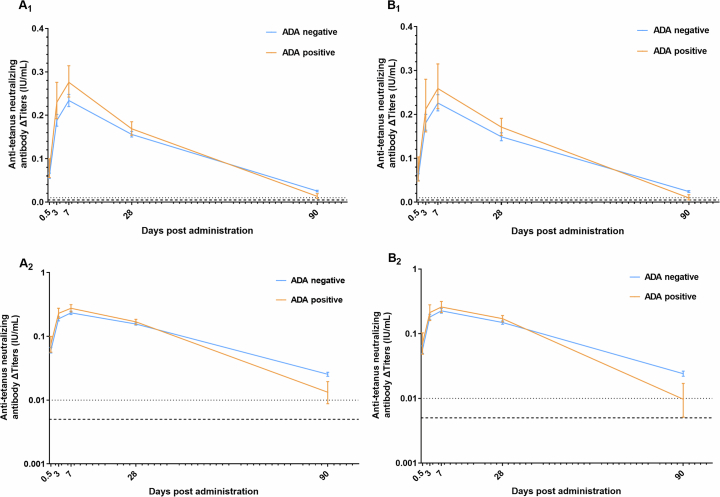
Anti-tetanus neutralizing antibody ΔTiters-time profiles of siltartoxatug by ADA status. The anti-tetanus neutralizing antibody ΔTiters-time profiles for the siltartoxatug group are shown for ADA positive participants (n = 58) and ADA negative participants (n = 379), with the y-axis presented on a linear scale (**a1**) and a logarithmic scale (**a2**). Similarly, profiles for participants with baseline antibody titer <0.01 IU/mL in the siltartoxatug group (n = 228) and HTIG group (n = 33) are displayed on a linear scale (**b1**) and a logarithmic scale (**b2**). All participants included in the analysis received siltartoxatug and had evaluable ADA results. Antibody titers were measured using the TNM002 PD bioanalytical method. The dotted line at 0.01 IU/mL indicates the protective antibody threshold, while the dashed line indicates the lower limit of quantification for the bioanalytical method used for siltartoxatug (0.005 IU/mL). The ΔTiters are presented as the geometric mean ± 95% CI, with error bars indicating the 95% CI.

**Extended Data Fig. 2:**
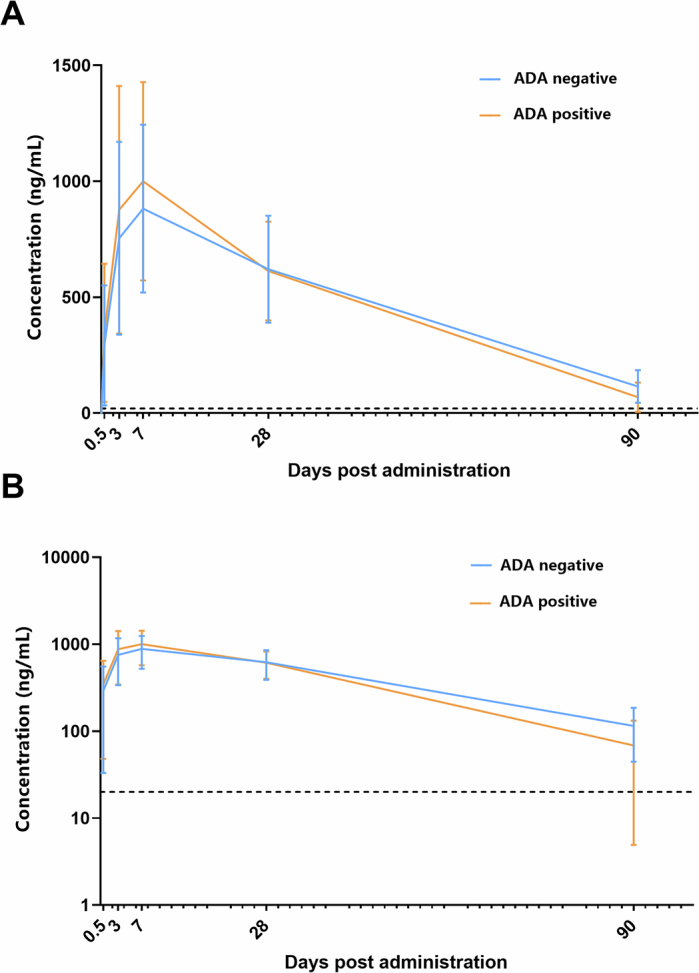
Pharmacokinetic concentration-time profiles by ADA positive and negative (pharmacokinetic set). The siltartoxatug serum concentration-time profiles was presented by ADA-positive (n = 58) and ADA-negative (n = 378) participants, with y-axis plotted using a linear scale (**a**) and a logarithmic scale (**b**). Participants included in the analysis were those who received siltartoxatug and had at least 1 available post-administration data for pharmacokinetics. Data are presented as arithmetic mean ± arithmetic standard deviation. The dashed line indicates the lower limit of quantification for the bioanalytical method for measuring the siltartoxatug concentration (20 ng/mL).

Additional analyses were performed to assess the potential implications of ADA responses on the intended effect of siltartoxatug. The ΔTiter–time profile of anti-tetanus neutralizing antibodies (measured by the TNM002 PD bioanalytical method) was generally similar between the ADA-positive and ADA-negative subgroups (Extended Data Fig. 1). At 90 days postadministration, ΔTiter was slightly lower in the ADA-positive subgroup, particularly in those who co-administered with tetanus vaccines. However, the geometric mean ΔTiter (0.00860) remained twice as high as that of the HTIG group (0.00429) (Table 2, Extended Data Fig. 1 and Extended Data Table 7). The siltartoxatug concentrations, summarized in Extended Data Table 8, were comparable between the ADA-positive and ADA-negative subgroups (Extended Data Fig. 2).

### Sensitivity analyses to primary outcome

Additional analyses were conducted to verify the robustness of the primary efficacy results, including a sensitivity analysis using the stratified Newcombe Wilson method, with supplementary analysis I using the ‘while on treatment strategy’ to handle intercurrent events, and supplemental analysis II in the per-protocol set. All analyses yielded consistent results (Supplementary Table 1).

## Discussion

The study met its primary objective by demonstrating that siltartoxatug was superior to HTIG for prophylaxis against tetanus in patients requiring passive immunization after injuries, using a surrogate endpoint of serum anti-tetanus neutralizing antibody levels. The results support that siltartoxatug could serve as a viable alternative to HTIG and TAT as the standard of care for passive immunization against tetanus. To our knowledge, this is the largest randomized clinical trial evaluating passive immunizing agents for tetanus postexposure prophylaxis.

Immunity to tetanus depends on the presence of antibodies. It is widely accepted that the minimum serum level of antibody required for protection against tetanus, as measured by neutralization assays, is 0.01 IU ml^−1^ (ref. ^30^). Thus, it is warranted to use anti-tetanus neutralizing antibodies as a surrogate endpoint to evaluate the efficacy of tetanus prophylactic drugs. The in vivo neutralization assay represents the gold standard for quantifying anti-tetanus antibody levels. In this established method, mice are injected with various dilutions of test sera that have been incubated with a lethal dose of tetanus toxin. Despite being considered a gold standard, this method has several limitations. Factors such as the subjective nature of the assay’s endpoints (for example, disease symptoms or death of the mice), the potency of the toxin and mouse weight can influence the results and antibody levels. Most critically, the high costs, labor-intensive processes and time-consuming nature of this method make it infeasible for large-scale clinical trials. In light of these challenges, the WHO advocates developing alternative in vitro techniques that should be verified against the in vivo neutralization method wherever possible^30^. In our study, two in vitro bioanalytical methods were developed and validated for measuring anti-tetanus neutralizing antibodies in both the siltartoxatug and HTIG groups. These methods demonstrated a strong correlation with in vivo neutralization assays in mice. Therefore, the antibody titers determined by both methods can be compared directly (data to be published separately). Given that some participants may have pre-existing antibodies, ∆Titer (defined as a difference, calculated as postdose titer minus baseline titer) was used to reflect and compare antibody titers provided solely by the two passive immunizing agents, siltartoxatug and HTIG.

The study results revealed that, from 3 to 28 days after administration, both 10 mg of siltartoxatug and 250 IU of HTIG exhibited good protective effect, with more than 95% of participants achieving anti-tetanus neutralizing antibody ∆Titers greater than the minimal protective level (0.01 IU ml^−1^). This finding aligns with previous studies indicating that 250 IU of HTIG is the lowest dose that provides 4 weeks of passive protection above the nominal threshold^33^. However, compared to the HTIG group, a significantly higher proportion of participants in the siltartoxatug group achieved ∆Titers ≥ 0.01 IU ml^−1^ at both the earliest (12 h postadministration) and latest (90 days postadministration) timepoints (95.4% versus 53.2% at 12 h and 91.5% versus 10.1% at 90 days). These results suggest that siltartoxatug provides better protection than HTIG in both the short- and long-term after injury. This is clinically relevant because, although most tetanus cases have an incubation period of 3–21 days postexposure, approximately 10% occur within 2 days and 3% occur after 30 days, with the longest reported incubation period being 178 days^10^.

Based on the proposed mechanism of protection, the level of antitoxin required to prevent tetanus likely correlates with the severity of the infection and the amount of toxin produced. HTIG (250 IU) is considered the minimum dose required for passive protection against tetanus^34^. However, patients with severe, heavily contaminated wounds, or those with a delay in treatment, may have higher levels of tetanus toxin in the body, rendering 250 IU insufficient to provide immunity. Death from tetanus has been reported despite the administration of HTIG 250 IU^35^. Our study demonstrated that 10 mg of siltartoxatug generated significantly higher (approximately five- to sevenfold) anti-tetanus neutralizing antibody titers compared with 250 IU of HTIG. Therefore, siltartoxatug 10 mg is expected to offer superior protection for higher-risk patients, including those with severe wounds, heavily contaminated wounds or delayed treatment, without requiring dose escalation.

According to clinical guidelines^5,36^, the tetanus vaccine should be administered simultaneously with passive immunization for wounded patients who have not completed the primary tetanus vaccination series or whose vaccination history is uncertain. A previous study showed that when HTIG 250 IU was administered concomitantly with tetanus vaccines, the onset of active immunity may be delayed by approximately 2 weeks, although it does not substantially suppress active immunity after the second dose of vaccination given 28 days later^33^. Our study revealed that when the tetanus vaccine was administered with siltartoxatug, higher levels of anti-tetanus antibodies (polyclonal antibodies induced by the vaccine) were elicited compared to HTIG plus vaccine, both at 28 days (2.09 IU ml^−1^ versus 0.504 IU ml^−1^) and 90 days (2.29 IU ml^−1^ versus 1.24 IU ml^−1^) postadministration. These results suggest that siltartoxatug may have less inhibitory effect on the induction of antibody response when administered concomitantly with tetanus vaccine, compared to HTIG.

Furthermore, the study confirmed that siltartoxatug demonstrated an acceptable safety profile with good tolerability and low immunogenicity. Allergic reactions remain a clinical concern with passive tetanus immunotherapies, whereas data are limited on allergic reactions following HTIG administration^37^. Our data indicated an allergic reaction rate of 0.9% (2 of 221) in the HTIG group, compared to 0.2% (1 of 440) in the siltartoxatug group. The siltartoxatug concentration-time profile observed in this phase 3 trial was consistent with the typical characteristics of an IgG1 monoclonal antibody^38^. The full pharmacokinetic properties of siltartoxatug, based on data from the phase 1 and phase 2 studies, will be published separately.

In summary, the results demonstrate that siltartoxatug exhibits a favorable risk–benefit profile, supporting its use in a broader clinical population following market approval.

As a recombinant native human mAb, siltartoxatug can be manufactured in large quantities with high specificity and consistency and possesses minimal biohazard potential. It can be stored at the same temperatures (2–8 °C) as HTIG. Siltartoxatug is expected to be more cost-effective and significantly address the current global supply shortage of plasma-derived HTIG, while effectively mitigating the risk of blood-borne infections. Furthermore, HTIG may interfere with the development of an immune response to live attenuated virus vaccines, such as rubella, mumps and varicella^39^. In contrast, siltartoxatug contains monoclonal antibodies specific to the tetanus toxin and does not interfere with these vaccines.

This trial has some limitations. First, using the tetanus protection rate as the primary endpoint was not feasible due to the low incidence of tetanus and limited sample size in a clinical trial setting. A surrogate endpoint was used instead. Although no cases of tetanus occurred in the trial, large-scale postmarketing studies are needed to evaluate the protective effectiveness in real-world setting. Second, the study limited the time window from injury to drug administration to <24 h. Future postmarketing studies should further assess the impact of delayed administration.

In conclusion, this phase 3 trial established siltartoxatug as an effective and safe option for passive tetanus immunization. Siltartoxatug may represent a more accessible and standardized alternative to current therapies, with great potential to replace plasma-derived HTIG and equine tetanus antitoxin in clinical practice.

## Methods

### Trial design and treatment

This was a randomized, double-blind, parallel-group, active-controlled, phase 3 study to compare the efficacy and safety of siltartoxatug and HTIG as prophylaxis against tetanus in patients with tetanus-prone wounds. Eligible participants were randomized at a 2:1 ratio to receive a single intramuscular gluteal injection of siltartoxatug 10 mg or HTIG 250 IU. Randomization was stratified based on whether or not the adsorbed tetanus vaccine was concomitantly administered, as determined at randomization. In the current clinical practice in China, the proportion of patients concomitantly receiving tetanus vaccines is low. Furthermore, antibodies induced by the tetanus vaccine could interfere with the comparison of antibody levels produced by the two passive immunizing agents, siltartoxatug and HTIG. To mitigate this, the study limited concomitant vaccination to approximately 10% of participants, administered exclusively at four designated study sites certified for vaccine administration. The randomization code was generated by an independent randomization specialist. Investigators registered participants and assigned them according to the randomization code obtained from an Interactive Response Technology system. Participants, investigators and study site personnel (apart from the unblinded pharmacist and/or unblinded study nurses responsible for drug preparation and administration) remained blinded to all randomization assignments throughout the study. Siltartoxatug and HTIG were both administered via intramuscular gluteal injection. Due to the different volumes of the two drugs, measures were taken to block the participants’ line of sight during the injection process to maintain blinding. Both study drugs were administered over 10 (±1) s.

The study lasted for approximately 106 days for each participant and included a screening period, a treatment period (within 24 h after injury) and a follow-up period (105 days). Clinical data were captured electronically using Medidata Rave EDC v.2022.3.0–v.2023.1.4. Serum samples were collected at the following timepoints: preadministration, and 12 h, 3, 7, 28 and 90 days postadministration, to analyze both siltartoxatug and HTIG anti-tetanus neutralizing antibody levels, as well as siltartoxatug concentration. For immunogenicity analysis, samples were collected at preadministration and at 7, 28 and 90 days postadministration. The samples were analyzed at a central laboratory.

The study was conducted at 28 hospitals across China in accordance with the Declaration of Helsinki and Good Clinical Practice guidelines. The study protocol (Supplementary Information) was approved by the independent ethics committees of two lead study sites, Peking University People’s Hospital and The First Affiliated Hospital of Guangzhou Medical University, as well as by the ethics committee of each participating study site (detailed in ‘List of ethical committees’; Supplementary Information). The trial is registered at ClinicalTrials.gov (NCT05664750).

### Participants

Eligible participants must meet all the following inclusion criteria:

1. Voluntary signing of the informed consent form;
2. Male or female participants aged ≥18 years;
3. Participants with unclean or contaminated wounds resulting from various injuries, including incised wounds, burns, traffic-related injuries, animal scratches and bites, who required passive immunization for tetanus prophylaxis;
4. The time from the injuries to study drug administration was required to be less than 24 h;
5. Sexually active women of childbearing potential who are at risk of pregnancy, as well as men, must agree to use at least one of the highly effective contraceptive methods throughout the study and for 150 days after dosing.

Participants were excluded if they met any of the following criteria:

1. All wounds were clean;
2. Requirement for HTIG 500 IU due to severe contaminated wounds, as determined by the investigator;
3. Suspected or diagnosed tetanus;
4. Fever (body temperature ≥38 °C) in the 3 days before dosing;
5. History of receiving at least three doses of tetanus toxoid or a tetanus toxoid-containing vaccine;
6. History of tetanus infection;
7. Previously diagnosed with IgA deficiency with anti-IgA antibodies;
8. Receipt of immunoglobulins, blood or blood products within 6 months before dosing, or planned to receive live viral vaccines within 3 months after dosing;
9. Participants with hemorrhagic conditions or at high risk of bleeding, clinically relevant active bleeding, abnormal platelet function, prothrombin time >3 s above the upper limit of normal, or platelet count <100 × 10^9^ l^−1^ (except for bleeding associated with injury);
10. Use of anticoagulants in the 3 weeks before dosing;
11. Pregnant female participants; female participants of childbearing potential who planned to become pregnant during the study; breastfeeding female participants;
12. Current alcohol abuse, drug abuse or drug addiction;
13. Known or suspected allergy to the test product or its excipients, or a history of allergy to human immunoglobulin products or other therapeutic monoclonal immunoglobulins;
14. Participation in other clinical studies involving investigational drugs or devices within 3 months or five times the drug’s half-life (whichever was longer) before dosing, except for observational or noninterventional studies;
15. Investigator site staff, sponsor employees directly involved in the study, site staff under the investigator’s supervision and their family members;
16. Any other factors deemed by the investigator to render the participant ineligible for the study.

All participants provided written informed consent before enrollment, including consent for reporting and sharing individual-level data. Participants’ sex was determined based on self-report and official identification records before being documented in the electronic case report form. Study objectives and endpoints were designed regardless of sex.

### Endpoints and assessment

The primary efficacy endpoint was the increase of anti-tetanus neutralizing antibody titer from baseline (ΔTiter) at 12 h postadministration. The estimand for the primary efficacy endpoint was the proportion of participants with anti-tetanus neutralizing antibody ΔTiter ≥0.01 IU ml^−1^ at 12 h postadministration, in all randomized participants who have received study drug.

The secondary efficacy endpoint was the tetanus protection rate (1 − tetanus incidence) within 28 days postadministration. Tertiary efficacy endpoints included the anti-tetanus neutralizing antibody ΔTiter at 3, 7, 28 and 90 days postadministration and the tetanus protection rate within 90 and 105 days postadministration. Since the antibodies induced by tetanus vaccine could interfere with the comparison of anti-tetanus neutralizing antibody levels between siltartoxatug and HTIG, analyses for tertiary efficacy endpoints were conducted in patients who had not received tetanus vaccine by the corresponding timepoints. The investigators assessed potential occurrence of tetanus through patient interviews conducted at each study visit.

The safety assessment included AEs, SAEs, laboratory tests, vital signs, physical examinations and electrocardiograms. The severity of an AE was classified into three grades: mild (asymptomatic or mild symptoms; clinical or diagnostic observations only; or intervention not indicated), moderate (requiring small, local or noninvasive treatment; restricted in age-appropriate instrumental activities of daily living) and severe (serious or clinically consequential but not immediately life-threatening; requiring hospitalization or prolongation of hospitalization; disabling; restricted in self-care activities of daily living).

An SAE was defined as an untoward medical event that met any of the following criteria after a participant received the study drug at any dose: resulting in death, life-threatening, hospitalization or prolongation of hospitalization, permanent or clinically consequential disability/dysfunction, congenital anomalies/birth defects or important medical event.

The immunogenicity was assessed via the presence of ADAs in participants who received siltartoxatug. The pharmacokinetic endpoint was the serum siltartoxatug concentration.

Two quantitative determination methods were developed for the siltartoxatug and HTIG groups to measure the levels of anti-tetanus neutralizing antibodies. A quantitative ligand-binding electrochemiluminescence assay using a Meso Scale Discovery (QUICKPLEX SQ120MM, model 1300) platform was utilized to quantify anti-tetanus neutralizing monoclonal antibodies in the siltartoxatug group, referred to as the TNM002 PD bioanalytical method. A quantitative ligand-binding chemiluminescence assay, referred to as the HTIG PD bioanalytical method, was developed to detect anti-tetanus neutralizing polyclonal antibodies produced by participants in the HTIG group. The titers determined by the two ligand-binding assays are comparable, as both have been validated for consistency with the in vivo neutralization assay in mice, which is considered the gold standard method to assess the anti-tetanus antibody levels^30^.

The anti-tetanus neutralizing antibody ∆Titer in each group was calculated as the postadministration value at each timepoint minus the baseline value measured by the respective bioanalytical method. Furthermore, the HTIG PD bioanalytical method was also utilized to evaluate the levels of anti-tetanus antibodies (polyclonal antibodies) in the subgroup of participants who received the concomitant adsorbed tetanus vaccine, as well as to measure the baseline anti-tetanus antibody levels of all participants.

### Statistical methods

Sample size calculation was based on the following hypotheses and assumptions: (1) the lower limit of the 95% CI of the difference (siltartoxatug group vs HTIG group) in the primary efficacy outcome would be >0; (2) the proportion of participants with ∆Titer ≥ 0.01 IU ml^−1^ at 12 h after receiving siltartoxatug and HTIG would be 91.8% and 55.9%, respectively, based on the results of the phase 2 trial; (3) a 2:1 randomization ratio between the siltartoxatug and HTIG groups and a one-sided type I error of 0.025; (4) the dropout rate was expected to be 20% and (5) siltartoxatug required adequate exposure during clinical development to fully understand its safety. The planned sample size was 675 participants, that is, 450 in the siltartoxatug group and 225 in the HTIG group. This would provide a power of >99% to evaluate the above efficacy hypothesis, while ensuring adequate exposure of siltartoxatug to evaluate its safety.

The efficacy analysis was conducted in the full analysis set, which included all randomized participants who received the study drugs. Additionally, a per-protocol set analysis was performed, encompassing all participants from the full analysis set who did not have important protocol deviations that could substantially impact the primary efficacy endpoint. Safety was analyzed in the safety set, including all treated participants with at least one postadministration safety evaluation. Immunogenicity was analyzed in all randomized participants who received siltartoxatug and had available immunogenicity data for at least one postadministration timepoint (immunogenicity set).

For primary and secondary endpoint analyses, the proportion of participants with ΔTiter ≥ 0.01 IU ml^−1^ and the tetanus protection rate were calculated, and the corresponding 95% CI was estimated using the Clopper–Pearson method. A 95% CI for intergroup difference was calculated using the Miettinen–Nurminen method, stratified by whether concomitantly administered with the tetanus vaccine as determined at randomization. A *P* value was reported for the primary efficacy endpoint. The following analyses were performed to verify the robustness of primary efficacy analysis, guided by ICH E9 (R1): a sensitivity analysis using stratified Newcombe Wilson method for intergroup difference 95% CI construction, supplementary analysis I using ‘while on treatment strategy’ to handle the intercurrent events compared with ‘treatment policy strategy’ used for the primary efficacy analysis, and supplementary analysis II conducted in the per-protocol set in lieu of the full analysis set.

The tertiary efficacy endpoints summarized the anti-tetanus neutralizing antibody ΔTiter level at different timepoints other than 12 h, conducted in participants who had not received the tetanus vaccine by the corresponding timepoint, since the antibodies produced by the vaccine may interfere with the antibody assessment of the study drugs. The proportion of participants with ΔTiter ≥ 0.01 IU ml^−1^ was analyzed using the same methods as the primary endpoint. A mixed-effect model for repeated measures was used to compare and analyze ΔTiter between groups at each scheduled timepoint.

In the subgroup consisting of participants who received the tetanus vaccine, the anti-tetanus antibody titers measured by HTIG PD bioanalytical method were summarized using descriptive statistics.

Safety and immunogenicity were summarized using descriptive statistics. All statistical analyses were performed with SAS v.9.4. The statistical analysis plan is available in Supplementary Information.

### Reporting summary

Further information on research design is available in the Nature Portfolio Reporting Summary linked to this article.

## Online content

Any methods, additional references, Nature Portfolio reporting summaries, source data, extended data, supplementary information, acknowledgements, peer review information; details of author contributions and competing interests; and statements of data and code availability are available at 10.1038/s41591-025-03791-8.

## Supplementary information

Supplementary Information List of ethic committees, Supplementary Fig. 1 and Table 1, Clinical study protocol and Statistical analysis plan.

Reporting Summary

## Figures and Tables

**Extended Data Table 1:**
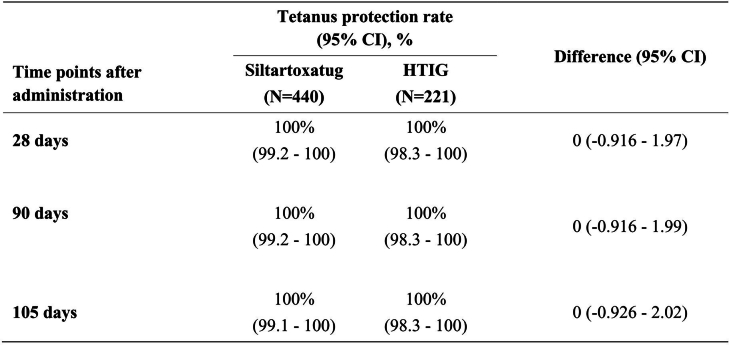
Anti-tetanus protection rate within 28, 90, and 105 days after administration

**Extended Data Table 2:**
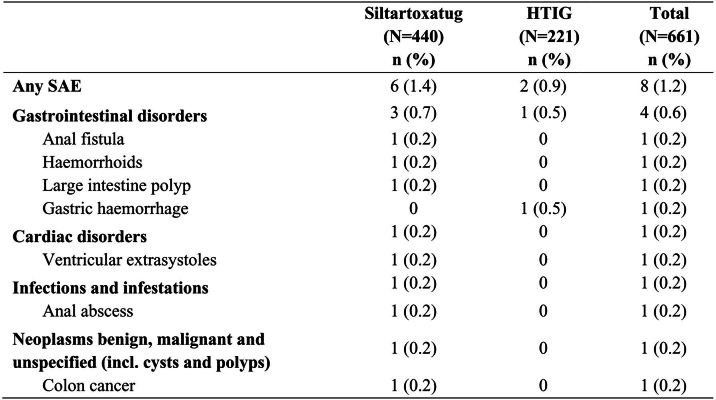
Serious adverse events by System Organ Class and Preferred Term

Preferred terms were defined according to the Medical Dictionary for Regulatory Activities terminology version 26.0, coded for all adverse events during the study.

**Extended Data Table 3:**
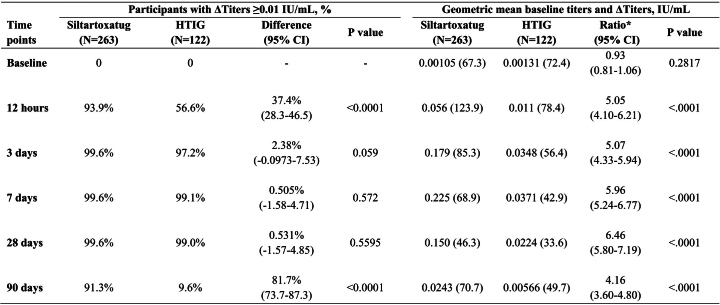
Summary of anti-tetanus neutralizing antibody ΔTiters level after study drug administration in participants with baseline antibody titers <0.01 IU/mL

CI, confidence interval.

The baseline titers and the ΔTiters (increase of titers from baseline) at different time points are presented as the geometric mean (% coefficient of variation).

The ΔTiters for the siltartoxatug group were calculated as the post-administration antibody titers minus the baseline antibody titers, measured using the TNM002 PD bioanalytical method. Similarly, the ΔTiters for the HTIG group were calculated based on antibody titers measured by the HTIG PD bioanalytical method.

For tertiary efficacy endpoint analyses, participants who concomitantly received the tetanus vaccine in the siltartoxatug group (n = 47) and HTIG group (n = 24) were excluded to avoid interference.

The proportion of participants with ΔTiters ≥0.01 IU/mL were calculated, and the corresponding 95% CI was constructed using the Clopper–Pearson method. A 95% CI for intergroup difference was calculated using Miettinen–Nurminen method. A mixed-effect model for repeated measures was used to compare and analyze ΔTiters between groups at each scheduled time point. All tests were two-sided. No adjustments were made for multiple comparisons.

**Extended Data Table 4:**
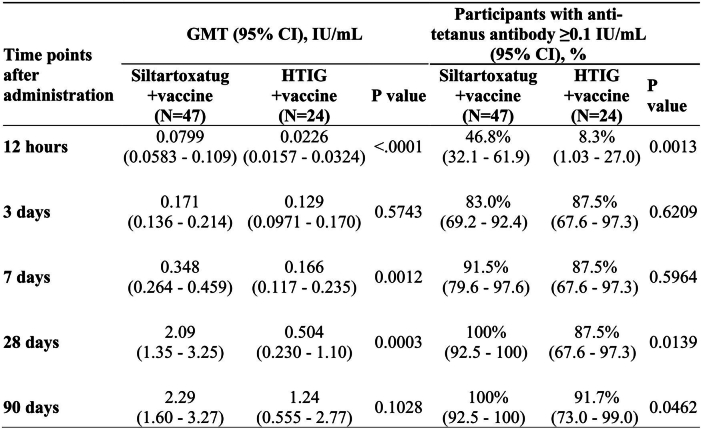
Anti-tetanus antibodies in participants co-administered with tetanus vaccine at different time points after administration

GMT, geometric mean titer.

The GMTs are presented as the geometric mean (% coefficient of variation).

The anti-tetanus antibody (polyclonal antibody) titers were measured using the HTIG PD bioanalytical method for both vaccine combination subgroups.

The proportion of participants with anti-tetanus antibody ≥0.1 IU/mL were calculated, and the corresponding 95% CI was constructed using the Clopper–Pearson method. A 95% CI for intergroup difference was calculated using Miettinen–Nurminen method. A mixed-effect model for repeated measures was used to compare and analyze anti-tetanus antibody between groups at each scheduled time point. All tests were two-sided. No adjustments were made for multiple comparisons.

**Extended Data Table 5:**
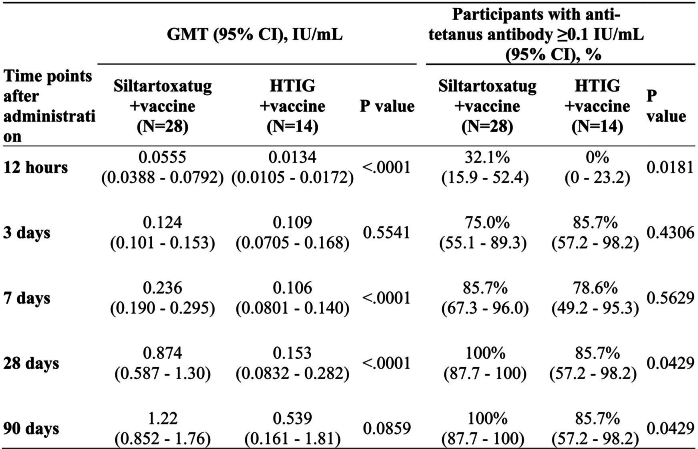
Anti-tetanus antibodies in participants co-administered with tetanus vaccine at different time points after administration, with baseline antibody titers < 0.01 IU/mL

GMT, geometric mean titer.

The GMTs are presented as the geometric mean (% coefficient of variation).

The anti-tetanus antibody (polyclonal antibody) titers were measured using the HTIG PD bioanalytical method for both vaccine combination subgroups.

The proportion of participants with anti-tetanus antibody ≥0.1 IU/mL were calculated, and the corresponding 95% CI was constructed using the Clopper–Pearson method. A 95% CI for intergroup difference was calculated using Miettinen–Nurminen method. A mixed-effect model for repeated measures was used to compare and analyze anti-tetanus antibody between groups at each scheduled time point. All tests were two-sided. No adjustments were made for multiple comparisons.

**Extended Data Table 6:**
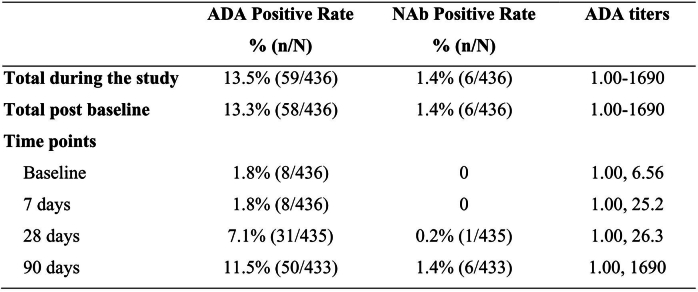
Summary of immunogenicity of siltartoxatug (immunogenicity set)

ADA, anti-drug antibody; NAb, neutralizing antibodies. N: number of participants in the immunogenicity set with available data.

**Extended Data Table 7:**
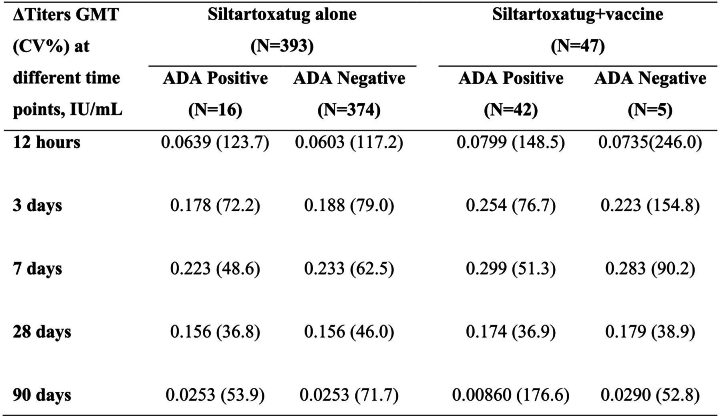
Comparison of anti-tetanus neutralizing antibodies between ADA-positive and ADA-negative participants, by siltartoxatug alone and siltartoxatug+ vaccine

ADA, anti-drug antibody; CV, coefficient of variation; GMT, geometric mean titer.

The antibody titers were measured by TNM002 PD bioanalytical method.

**Extended Data Table 8:**
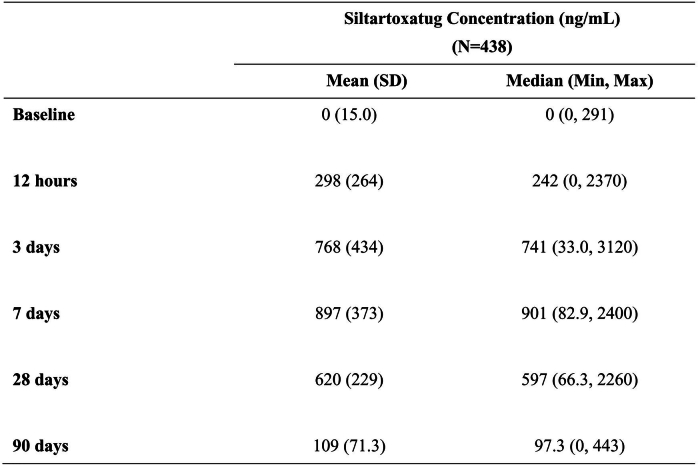
Summary of pharmacokinetic concentrations

Siltartoxatug pharmacokinetic concentrations were assessed in all randomized participants who received siltartoxatug and had at least one post-dose evaluable concentration.

## Data Availability

The data supporting the findings of this trial are available within the article and its Supplementary Information. To ensure protection of participant privacy and proprietary information, deidentified individual participant data are available under restricted access. All requests for additional data sharing must be reviewed by the lead study sites (Peking University People’s Hospital and The First Affiliated Hospital of Guangzhou Medical University), as well as the sponsor (Zhuhai Trinomab Pharmaceutical Co., Ltd) to assess any potential intellectual property or confidentiality concerns. Data requests should be sent to the corresponding authors, accompanied by a detailed proposal outlining the intended use of the data. Responses to such requests can be expected within 1 month. A signed data access agreement with the sponsor is required before data sharing.

## Code availability

The use of custom code is not applicable in this paper.
