# Supplementary material for: Recombinant monoclonal antibody siltartoxatug versus plasma-derived human tetanus immunoglobulin for tetanus: a randomized, double-blind, active-controlled, phase 3 trial
Source: Nat Med. 2025 Jul 8;31(8):2673–81. doi: 10.1038/s41591-025-03791-8 (PMC12353795; doi:10.1038/s41591-025-03791-8)
Supplement: Supplementary file 1 — List of ethic committees, Supplementary Fig. 1 and Table 1, Clinical study protocol and Statistical analysis plan. [file 41591_2025_3791_MOESM1_ESM.pdf]

nature medicine

Supplementary information https://doi.org/10.1038/s41591-025-03791-8

Recombinant monoclonal antibody siltartoxatug versus plasma-derived human tetanus immunoglobulin for tetanus: a randomized, double-blind, active-controlled, phase 3 trial

In the format provided by the authors and unedited

Supplementary Information

List of Ethic Committees

Supplementary Fig. 1

Supplementary Table 1

Clinical Study Protocol

Statistical Analysis Plan

List of Ethic Committees

**Ethic Committees**	**Location**
Peking University People's Hospital	Beijing, China
The First Affiliated Hospital of Guangzhou Medical University	Guangzhou, China
Peking University First Hospital	Beijing, China
The First Affiliated Hospital of Shenzhen University, Shenzhen Second People's Hospital	Shenzhen, China
The Affiliated Nanhua Hospital, Hengyang Medical School, University of South China	Hengyang, China
Affiliated Hospital of Zunyi Medical University	Zunyi, China
The Third Affiliated Hospital of Guangzhou Medical University	Guangzhou, China
The Second Affiliated Hospital of Nanchang Medical University	Nanchang, China
Tongji Hospital of Tongji Medical College, Huazhong University of Science and Technology	Wuhan, China
Liuzhou Worker's Hospital	Liuzhou, China
The Second Affiliated Hospital of Anhui Medical University	Hefei, China
Yuncheng Central Hospital	Yuncheng, China
The First People's Hospital of Jinzhong	Jinzhong, China
The Central Hospital of Wuhan, Tongji Medical College, Huazhong University of Science and Technology	Wuhan, China
The Second Xiangya Hospital of Central South University	Changsha, China

Shanxi Bethune Hospital, Shanxi Academy of Medical Sciences, Third Hospital of Shanxi Medical University	Taiyuan, China
PKUcare Luzhong Hospital	Zibo, China
Zhejiang Provincial People's Hospital	Hangzhou, China
Guangdong Provincial Hospital of Chinese Medicine	Guangzhou, China
Renmin Hospital of Wuhan University	Wuhan, China
Nanfang Hospital, Southern Medical University	Guangzhou, China
Beijing Tiantan Hospital, Capital Medical University	Beijing, China
Hefei First People’s Hospital	Hefei, China
Hainan General Hospital (Hainan Affiliated Hospital of Hainan Medical University)	Haikou, China
The First Affiliated Hospital, Sun Yat-sen University	Guangzhou, China
Guangzhou First People’s Hospital	Guangzhou, China
Zhongnan Hospital of Wuhan University	Wuhan, China

Supplementary Fig. 1 Histogram of change in anti-tetanus neutralizing antibody titers from baseline (ΔTiters, both raw and log-transformed) at 12 hours and 28 days after study treatment administration

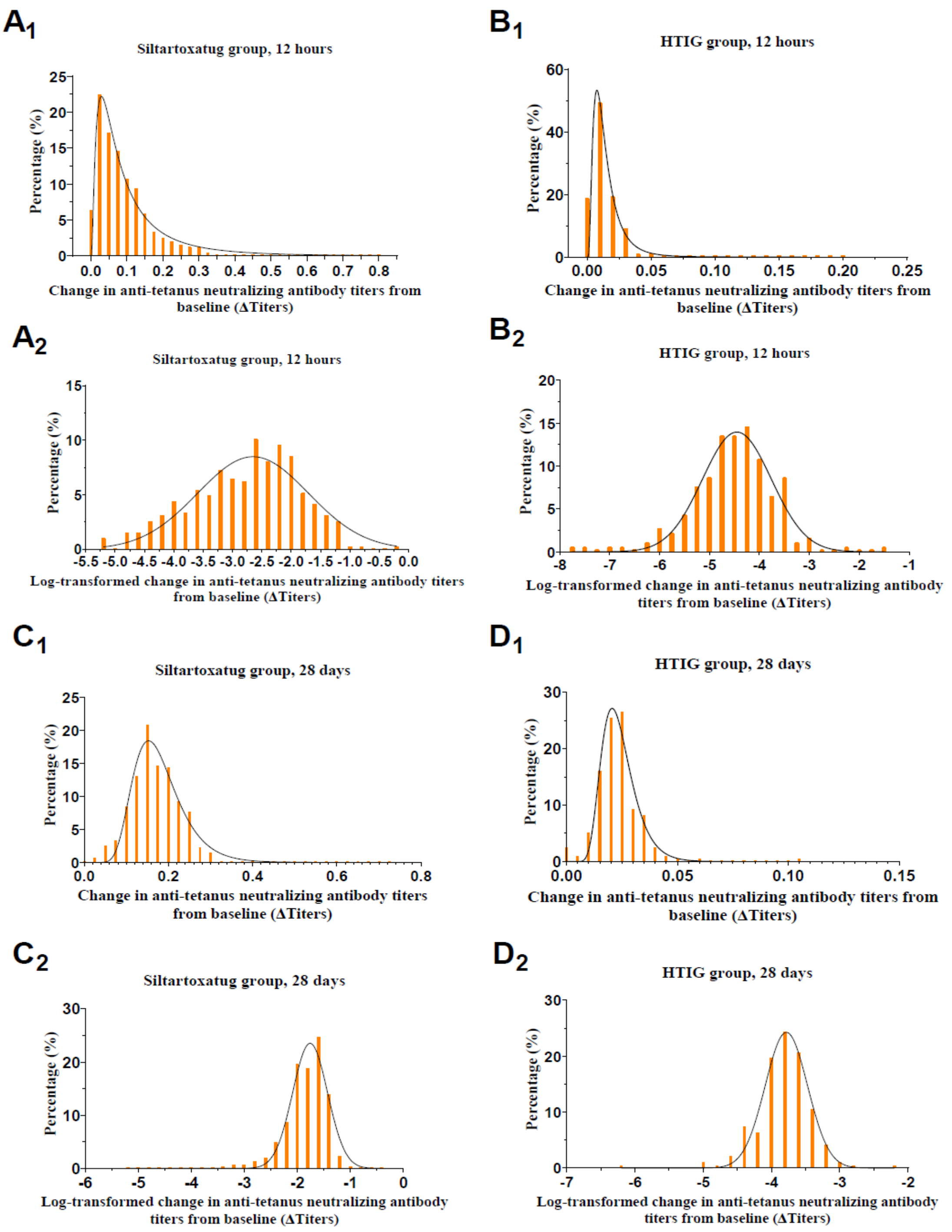

The histograms depict the log-normal distribution of anti-tetanus neutralizing antibody ΔTiters for both the siltartoxatug and HTIG groups, 12 hours (**A_1**&**A_2**; **B_1**&**B_2**) and 28 days (**C_1**&**C_2**; **D_1**&**D_2**) after study treatment administration.

Supplementary Table 1 Sensitivity and supplementary analyses for the primary efficacy analysis

	Participants with ΔTiters ≥0.01 IU/mL (95% CI [a]), %		Difference (95% CI)
	**Siltartoxatug (N=440)**	**HTIG (N=221)**	
**Sensitivity Analysis [b] (full analysis set)**	95.4% (93.0-97.2)	53.2% (46.4 - 59.9)	42.3% (35.3 - 49.0)
**Supplementary Analysis I [c] (full analysis set)**	95.4% (93.0 - 97.2)	52.1% (45.2 - 59.0)	43.3% (36.3 - 50.2)
**Supplementary Analysis II [d] (per-protocol set)**	95.4% (92.9 - 97.1)	52.1% (45.2 - 59.0)	43.2% (36.3 - 50.1)

[a] Clopper-Pearson confidence interval.

[b] The stratified Newcombe Wilson method was used for intergroup difference 95% CI construction of the sensitivity analysis, in contrast to the stratified Miettinen-Nurminen method used for the primary efficacy analysis.

[c] The 'while on treatment strategy' was used to handle intercurrent events, in contrast to the 'treatment policy strategy' used for the primary efficacy analysis.

[d] The per-protocol set was used instead of the full analysis set for the primary efficacy analysis. The per-protocol set was defined as participants in the full analysis set who did not have any important protocol deviations that may significantly affect the primary efficacy outcome.

A Multicenter, Randomized, Double-Blind, Parallel-Group, Active-Controlled Phase 3 Study to Compare the Efficacy and Safety of Passive Immunization with TNM002 Injection and Human Tetanus Immunoglobulin as Prophylaxis Against Tetanus

Protocol No.: TNM002-301

**Investigational Drug:**	TNM002 Injection
**Clinical Trial Approval Notice No.:**	2021LP00757
**Protocol No:**	TNM002-301
**Version No.:**	3.0
**Version Date:**	16-June-2023
**Study Phase:**	3
**Sponsor:**	Zhuhai Trinomab Pharmaceutical Co., Ltd.

[redacted]

TABLE OF CONTENTS

TABLE OF CONTENTS **1**
- List of Tables 4

SYNOPSIS **5**

SCHEDULE OF ACTIVITIES **12**

1. INTRODUCTION **15**
- 1.1. Background 15
  - 1.1.1. Tetanus 15
  - 1.1.2. Prophylaxis Against Tetanus 15
- 1.2. Introduction of Investigational Drug 16
  - 1.2.1. General Information of Investigational Drug 16
  - 1.2.2. Nonclinical Studies 16
  - 1.2.3. Previous Clinical Studies 19
- 1.3. Objective and Rationale 21
- 1.4. Benefit/Risk Assessment 22

2. OBJECTIVES AND ENDPOINTS **23**

3. STUDY DESIGN **25**
- 3.1. Overall Design 25
- 3.2. Rationale for the Design 26
  - 3.2.1. Study Population and Comparator 26
  - 3.2.2. Primary Endpoint 26
  - 3.2.3. Justification for Dose 27
  - 3.2.4. Proportion of Participants Receiving Adsorbed Tetanus Vaccine Concomitantly 28
  - 3.2.5. Stratified Randomization 29
  - 3.2.6. Study Drug Administration and Blinding 29

4. STUDY POPULATION **29**
- 4.1. Inclusion Criteria 29
- 4.2. Exclusion Criteria 30

5. STUDY DRUG **31**
- 5.1. Study Drug Information 31
- 5.2. Drug Dispensing and Preparation 31
- 5.3. Administration 32
- 5.4. Handling, Storage, and Accountability 32
- 5.5. Randomization and blinding 33
  - 5.5.1. Randomization 33
  - 5.5.2. Blinding 33
  - 5.5.3. Emergency Unblinding 33
  - 5.5.4. Routine Unblinding 33

5.6. Study Drug Compliance 34
5.7. Concomitant Medications and Therapies 34
5.7.1. Prohibited Medicines 34
5.7.2. Permitted During the Study 34
5.7.3. Concomitant administration of Vaccine 35
5.8. Early Withdrawal and Lost to Follow-up 35
5.8.1. Early Withdrawal from Study 35
5.8.2. Lost to Follow-up 36
6. STUDY PROCEDURES 36
6.1. Screening (within 24 hours prior to dosing) 36
6.2. Visit 1 (dosing) 37
6.3. Visit 2 (12 hours after dosing) 37
6.4. Visit 3 (3 days after dosing) 38
6.5. Visit 4 (7 days after dosing) 38
6.6. Visit 5 (28 days after dosing) & Visit 6 (90 days after dosing) 39
6.7. Visit 7 (105 days after dosing) 39
6.8. End of Study Definition 39
7. STUDY ASSESSMENTS 40
7.1. Efficacy Assessments 40
7.1.1. Assessment of Anti-Tetanus Neutralizing Antibody Titer 40
7.1.2. Assessment of Tetanus Protection Rate 40
7.2. Safety Assessment 40
7.2.1. Physical examination 41
7.2.2. Vital signs 41
7.2.3. 12-lead ECG 41
7.2.4. Safety Laboratory Assessments 41
7.2.5. Serum Pregnancy Test 42
7.2.6. Assessment of Injection Site 42
7.3. Pharmacokinetics 42
7.4. Pharmacodynamics 43
7.5. Immunogenicity Evaluation 43
7.6. Other Evaluations 43
7.6.1. Demographic Information 43
7.6.2. Medical, Treatment and Vaccination History 43
8. ADVERSE AND SERIOUS ADVERSE EVENTS 44
8.1. Definition of AE 44
8.2. Time Period of Collecting AE 45
8.3. Recording of AEs 45
8.3.1. AE 45

8.3.2. Start Date and Time ... 46
8.3.3. Assessment of Severity ... 46
8.3.4. Assessment of Causality ... 46
8.3.5. Action Taken with the Study Drug ... 47
8.3.6. Other Action Taken ... 47
8.3.7. Outcome and Date and Time of Outcome ... 47
8.4. Adverse Events of Special Interest ... 48
8.5. Special Precautions and Management of AEs ... 48
8.5.1. Anaphylaxis ... 48
8.6. DEFINITION OF SAE ... 49
8.7. Collecting and Reporting of SAE ... 50
8.8. Regulatory Reporting Requirements for SAEs ... 50
8.9. Exposure During Pregnancy ... 51
9. STATISTICAL ANALYSIS ... 51
9.1. Sample Size Determination ... 51
9.2. Analysis Set ... 52
9.3. Statistical Analysis ... 54
9.3.1. Efficacy Analysis ... 54
9.3.2. Safety Analysis ... 55
9.3.3. PK Analysis ... 56
9.3.4. Immunogenicity Analysis ... 56
9.4. Analysis Timeline ... 56
9.5. Data Monitoring Committee ... 56
10. REGULATORY, ETHICAL, AND STUDY OVERSIGHT CONSIDERATIONS .. 56
10.1. Regulatory and Ethical Considerations ... 56
10.2. Informed Consent Process ... 57
10.3. Data Protection ... 57
10.4. Study and Site Closure ... 58
11. DATA HANDLING ... 58
11.1. Source Documents ... 58
11.2. Electronic Case Report Form ... 58
11.3. Data Management ... 59
11.4. Provision of Additional Information ... 59
12. DATA QUALITY ASSURANCE ... 59
13. REPORTING AND PUBLICATION ... 60
13.1. Clinical Study Report ... 60
13.2. Confidentiality and Ownership of Trial Data ... 60
13.3. Publication Policy ... 60

14. REFERENCES **62**
15. APPENDICES AND ANNEXES **63**
Appendix 1: Wound Exposure Category 63
Appendix 2: Diagnostic Basis for Non-neonatal Tetanus: 64
Appendix 3: Abbreviations 65
Annex 1: Package Insert for Tetanus Human Immunoglobulin 68
Annex 2: Package Insert for Adsorbed Tetanus Vaccine 68

LIST OF TABLES

Table 1: Laboratory Tests 42
Table 2: Probability of Observing at Least One Adverse Event at Different Sample Sizes and Different True Incidences of Adverse Events 52

Synopsis

**Investigational Drug**	TNM002 Injection
**Protocol Title**	A Multicenter, Randomized, Double-Blind, Parallel-Group, Active-Controlled Phase 3 Study to Compare the Efficacy and Safety of Passive Immunization with TNM002 Injection and Human Tetanus Immunoglobulin as Prophylaxis Against Tetanus
**Study Protocol No.**	TNM002-301
**Study Phase**	3
**Study Site**	The study is planned to be conducted at 20 to 30 study sites in China
**Study Objectives, Study Endpoints and Estimands**	(see table below)

Objectives	Endpoints	Estimands
**Primary Efficacy**		
To compare the efficacy of TNM002 Injection 10 mg with human tetanus immunoglobulin (HTIG) 250 IU as prophylaxis against tetanus following a single intramuscular (IM) injection	The increase of anti-tetanus neutralizing antibody titers from baseline at 12 hours after receipt of study drug	In all randomized subjects who have received study drug, the proportion of subjects with an increase of anti-tetanus neutralizing antibody titers (Δ titers) ≥ 0.01 IU/mL from baseline at 12 hours after receipt of study drug.<br>Subjects who are diagnosed with tetanus within 12 hours after receipt of study drug, or have received additional passive immunizing agent due to the original wound, or have experienced AE leading to study discontinuation within 12 hours after receipt of study drug, are considered to have a Δ titer < 0.01 IU/mL.
**Secondary Efficacy**		
To compare the clinical outcomes after administration of TNM002 10 mg with HTIG 250 IU as prophylaxis against tetanus following a single IM injection	Tetanus protection rate within 28 days after receipt of study drug	In all randomized subjects who have received study drug: Tetanus protection rate (1 - tetanus incidence) within 28 days after receipt of study drug
**Safety**		

		To compare the safety profile of TNM002 10 mg with HTIG 250 IU as prophylaxis against tetanus following a single IM injection	• AEs<br>• SAEs<br>• Laboratory tests, vital signs, physical examinations, and electrocardiograms (ECGs)	In all randomized subjects who have received study drug and having at least one post-dose safety evaluation<br>• Incidence of adverse events (AEs) for up to 105 days after receipt of study drug<br>• Incidence of treatment related AEs for up to 105 days after receipt of study drug<br>• Incidence of SAEs for up to 105 days after receipt of study drug<br>• Summary statistics and shift tables (if appropriate) of laboratory tests, vital signs, physical examinations, and electrocardiograms (ECGs) for up to 90 days after receipt of study drug
		**Tertiary**		
		To compare the protective antibody level of TNM002 10 mg with HTIG 250 IU as prophylaxis against tetanus at different time points following a single IM injection	The increase of anti-tetanus neutralizing antibody titers from baseline at 3, 7, 28 and 90 days after receipt of study drug	In all randomized subjects who have received study drug, and haven't received tetanus vaccine up to the respective time points:<br>• Proportion of subjects with Δ titers ≥ 0.01 IU/mL from baseline at 3, 7, 28 and 90 days after receipt of study drug, respectively<br>• Geometric mean of Δ titers from baseline at 12 hours, and 3, 7, 28 and 90 days after receipt of study drug, respectively<br>Subjects who are diagnosed with tetanus as of the respective time point, or have received additional passive immunizing agent due to the original wound, are considered to have an antibody titer of LLOQ/2 and a Δ titer < 0.01 IU/mL at that time point.
		To compare the long-term clinical outcomes after administration of TNM002 10 mg with HTIG 250 IU as prophylaxis against	Tetanus protection rate within 90 and 105 Days after receipt of study drug	In all randomized subjects who have received study drug: Tetanus protection rate (1 - tetanus incidence) within 90 and 105 days after receipt of study drug, respectively

<table>
<tr>
<td></td>
<td>
<table>
<tr><td>tetanus following a single IM injection</td><td></td><td></td></tr>
<tr><td>To evaluate the pharmacokinetic (PK) properties of TNM002 in subjects requiring passive tetanus passive immunization</td><td>Serum TNM002 concentrations in subjects following TNM002 administration</td><td>In all randomized subjects who have received TNM002, having at least one post-dose evaluable PK concentration, and without major protocol deviations that affect PK concentrations of the respective time point: Serum TNM002 concentration at baseline, 12 hours, and 3, 7, 28 and 90 days after receipt of TNM002, respectively</td></tr>
<tr><td>To evaluate the immunogenicity of TNM002 in subjects requiring tetanus passive immunization</td><td>Anti-drug antibodies (ADA) in subjects following TNM002 administration</td><td>In all randomized subjects who have received TNM002 and hav post-dose immunogenicity data: ADA positive rate at 7, 28 and 90 days after receipt of TNM002, respectively</td></tr>
</table>
Note: The increase of anti-tetanus neutralizing antibody titers from baseline in the TNM002 group is defined as: the post-dose values by quantitative detection of TNM002 serum titer using the MSD platform (referred to as TNM002 PD bioanalysis method) at respective time points minus the pre-dose baseline values;
The increase of anti-tetanus neutralizing antibody titers from baseline in the HTIG group is defined as: the post-dose values at respective time points by quantitative detection of HTIG serum concentration using the chemiluminescence method (referred to as HTIG PD bioanalysis method) minus the pre-dose baseline values.
</td>
</tr>
<tr>
<td>Study Design</td>
<td>
This is a phase 3, multicenter, randomized, double-blind, parallel-group, active-controlled study to compare the efficacy and safety of TNM002 10 mg and HTIG 250 IU as prophylaxis against tetanus in subjects with unclean or contaminated wounds caused by various injuries and who require passive immunization against tetanus.
Approximately 675 subjects are planned to be enrolled in this study. These subjects will be randomized and receive a single IM gluteal injection of TNM002 10 mg or HTIG 250 IU/mL in a 2:1 ratio. Randomization will be stratified by with or without concomitant administration of adsorbed tetanus vaccine. The proportion of subjects with concomitant adsorbed tetanus vaccine administration will be approximately 10%. The vaccine will be provided by study sites qualified to administer tetanus vaccines. It will be intramuscularly injected into deltoid muscle of upper arm according to the vaccine manufacture instructions.
The study personnel responsible for drug preparation and administration will be unblinded due to the apparent difference in the dosing volumes of TNM002 (0.5 mL) and HTIG (2.5 mL). During the study drug administration, measures should be taken to block subject's line of sight, in order to ensure that the subject cannot observe any identifying information of the study drug. The injection time for all study drugs should be 10 (± 1) seconds. The subjects, investigators, and the other study personnels at the study centers will be blinded throughout the study.
</td>
</tr>
</table>

	Before and after study drug administration, wound treatment should be performed in accordance with the clinical standard procedure. Concomitant medicine or other treatment required for injury itself or comorbidities are permitted during the study.<br><br>Serum samples for anti-tetanus neutralizing antibody and PK will be collected from all subjects at pre-dose, 12 hours, and 3, 7, 28 and 90 days after dosing. Serum samples for immunogenicity tests will be collected from all subjects at pre-dose, and 7, 28 and 90 days after dosing. These tests will be performed at a central bioassay laboratory.<br><br>During the visits, subjects will also undergo safety assessments including collection of AEs, physical examinations, vital signs, 12-lead ECGs, and safety laboratory tests (hematology, clinical chemistry, coagulation, and urinalysis), and the tetanus incidence will also be monitored. The last visit for this study is planned to be performed at 105 days after dosing and will be completed by telephone contact.<br><br>As the diagnosis of tetanus is mainly based on clinical symptoms and no reliable objective diagnostic criteria are available, a clinical adjudication committee consisting of clinical experts in the field of tetanus disease will be established. For subjects with tetanus-related symptoms, the final diagnosis will be made by experts in this committee after discussion.<br><br>The primary analysis is planned to be performed after the last enrolled subject has completed the visit at 28 days after dosing, in order to evaluate the efficacy and safety of the study drug. The analysis will be carried out by an independent unblinded team.
**Inclusion Criteria**	**Participants must meet all of the following inclusion criteria to be eligible for the study:**<br>1. Voluntary signing of the informed consent form;<br>2. Male or female subjects aged ≥ 18 years;<br>3. Participants with unclean or contaminated wounds caused by various injuries such as incised wound, burn, traffic wound, animal scratch and bite wounds who require passive immunization as prophylaxis against tetanus;<br>4. The time from above injury to study drug administration is expected less than 24 hours<br>5. Women of childbearing potential and at risk of pregnancy and males who are sexually active must agree to use at least one of the following highly effective contraceptive methods throughout the study and for 150 days after dosing, including:<br>• Intrauterine device (IUD);<br>• Oral, injectable, or implantable hormonal contraceptives;<br>• Barrier contraception + spermicide;<br>• Surgical contraceptive methods (e.g., vasectomy, salpingectomy, hysterectomy, etc.);<br>Women of no childbearing potential will be authorized to participate in this study if at least one of the following criteria are met:<br>• Documented hysterectomy and/or bilateral oophorectomy;<br>• Medically confirmed ovarian function failure;<br>• Postmenopausal, defined as follows: cessation of regular menses for at least 12 consecutive months with no alternative pathological or physiological cause.
**Exclusion Criteria**	**Participants who meet any of the following criteria will be excluded from the study:**

	1. All wounds are clean (Appendix 1);<br>2. Need to use HTIG 500 IU due to severe or serious contaminated wound, as judged by the investigator;<br>3. Suspect or diagnosed as tetanus;<br>4. Fever (body temperature ≥ 38 °C) within 3 days prior to dosing;<br>5. Prior vaccination history of ≥ 3 doses of tetanus toxoid or tetanus toxoid-containing vaccine;<br>6. Previously diagnosed with tetanus infection;<br>7. Previously diagnosed as IgA deficiency with anti-IgA antibodies;<br>8. Participants have received immunoglobulins, blood or blood products within 6 months prior to dosing; subjects plan to receive live viral vaccines within 3 months after dosing;<br>9. Participants with hemorrhagic factors, or at high risk of bleeding, including congenital hemorrhagic disorders (e.g., hemophilia) or any clinically significant active bleeding, or abnormal platelet function, or prothrombin time (PT) > 3 seconds above the ULN by coagulation tests, or platelet count < 100 × 10^9/L (except for bleeding associated with injury such as incised wound, burn, and traffic wound);<br>10. Have received anticoagulants within 3 weeks prior to dosing;<br>11. Pregnant women or women who plan to become pregnant during the study and nursing mothers;<br>12. Current alcohol abuse, drug abuse or drug addiction;<br>13. Known or suspected allergy to the investigational product or its excipients, or have a history of allergy to human immunoglobulin products or other therapeutic monoclonal immunoglobulins;<br>14. Have participated in other clinical studies with investigational drugs or devices within 3 months or 5 times the half-life of the drugs (whichever is longer) prior to dosing, except for observational or non-interventional clinical studies;<br>15. Investigator site staff or sponsor employees directly involved in the conduct of the study, site staff otherwise supervised by the investigator, and their respective family members.<br>16. Participants who are ineligible for the study due to other factors judged by the investigator.
**Planned number of subjects**	Approximately 675 subjects will be randomized such that approximately 540 evaluable subjects will complete the study.
**Study Duration**	The study duration will be approximately 106 days, including screening (< 24 hours) and follow-up (105 days).
**Study Drug**	**TNM002**<br>• Dosage form: Injection, solution<br>• Strength: 10 mg/0.5 mL/vial<br>• Route: IM gluteal injection<br>**Human Tetanus Immunoglobulin**<br>• Dosage form: Injection, solution<br>• Strength: 250 IU/2.5 mL/vial

	• Route: IM gluteal injection
**Statistical Analysis**	**Sample Size Estimation**<br>• **Primary Hypothesis:**<br>The hypothesis is that TNM002 has efficacy that is superior to HTIG as prophylaxis against tetanus. To be more specific, the lower limit of the 95% confidence interval (CI) of the difference (TNM002 group - HTIG group) in the proportion of subjects with Δtiters ≥ 0.01 IU/mL at 12 hours after the receipt of a single IM injection is > 0.<br>The sample size estimation is based on the above hypothesis and following assumptions: (1) The proportion of subjects with Δtiter ≥ 0.01 IU/mL at 12 hours after receipt of TNM002 and HTIG based on results of earlier clinical trials; (2) Due to the fact that HTIG is a well-studied product whose efficacy and safety does not need to be evaluated in large amount of subjects in a clinical trial setting, the randomization ratio between TNM002 and HTIG groups is 2:1; (3) One-sided Type I error of 0.025; (4) TNM002 requires adequate exposure during clinical development to fully understand its safety.<br>Taken together, the estimated sample size is approximately 540 subjects, i.e., 360 in the TNM002 group and 180 in the HTIG group. This will provide a power of >99% to evaluate the above efficacy hypothesis. The dropout rate is expected to be 20% in this study. Therefore, the planned enrollment will be 675 subjects, i.e., 450 in the TNM002 group and 225 in the HTIG group.<br>• **Secondary Hypothesis:**<br>The tetanus protection rate is assumed to be 99.99% within 28 days post-dose in the HTIG group and 99.999% within 28 days post-dose in the TNM002 group. The current sample size will not provide sufficient power to test a formal statistical hypothesis in terms of tetanus protection rate between TNM002 and HTIG.<br>• **Safety Hypothesis:**<br>Table 2 shows the probability of observing at least 1 AE for a given true event rate of a particular AE, for various sample sizes. For example, if the true AE rate is 1%, with 300 subjects in the TNM002 group, there is a 95.1% probability of observing at least 1 AE.<br><br>**Analysis Set:**<br>• **All-Screened Set**: all subjects who have signed the informed consent form.<br>• **Intention-to-Treat (ITT) population**: all subjects who have been randomized.<br>• **Full analysis set (FAS):** all randomized subjects who have received the study drug.<br>• **Safety set (SS):** all randomized subjects who have received the study drug with at least one post-dose safety evaluation.<br>• **Immunogenicity set (IAS):** all randomized subjects who have received TNM002 with at least one post-dose immunogenicity data point.<br>• **Pharmacokinetic set (PKS):** all randomized subjects who have received TNM002 with at least one post-dose evaluable PK concentration. If the PK concentration results are affected by important protocol deviations, all or partial PK data of corresponding subjects will not be included in the PK analysis.

- **Per Protocol Set (PPS):** all subjects in the FAS who do not have any important protocol deviations considered to have a major effect on the primary efficacy endpoint.

Primary Efficacy Analysis

In all randomized subjects who have received the study drug, the proportion of subjects with an increase of anti-tetanus neutralizing antibody titers (Δ titers) ≥ 0.01 IU/mL from baseline at 12 hours after receipt of study drug and its two-sided 95% CI will be calculated for each treatment group. Subjects who are diagnosed with tetanus within 12 hours after receipt of study drug, or have received additional passive immunizing agent due to the original wound, or have experienced AE leading to study discontinuation within 12 hours after receipt of study drug, are considered to have a Δ titer < 0.01 IU/mL.

Secondary Efficacy Analysis

In all randomized subjects who have received study drug, the tetanus protection rate within 28 days after receipt of study drug and its two-sided 95% CI will be calculated for each treatment group. The difference in tetanus protection rate between the TNM002 and HTIG groups and its two-sided 95% CI will be calculated.

Safety Analysis

AEs will be coded using the Medical Dictionary for Regulatory Activities (MedDRA) version 25.0 or higher. The number and incidence of all AEs, AEs related to the study drug, and SAEs in both treatment groups will be calculated and presented by system organ class (SOC) and preferred term (PT) based on the safety set.

The quantitative safety variables such as laboratory tests, vital signs, ECGs, and their changes from baseline will be summarized using descriptive statistics. The changes from baseline in safety variables such as laboratory tests, ECGs, and physical examinations will also be described using the shift tables. The test items with abnormal values and clinical significance will be listed.

In the safety analysis, subjects will be analyzed according to the study drug they actually receive.

Pharmacokinetic Analysis

Drug concentrations will be summarized descriptively by nominal collection time points for serum samples.

In order to describe the PK properties and to identify and quantify the source and extent of PK variation, the nonlinear mixed effect model (NONMEM) will be used to establish a population PK model of TNM002. PK/PD analysis will also be carried out, if appropriate.

Analysis timeline

The primary analysis will be performed after the last enrolled subject has completed the Day 28 visit. This analysis includes the final analysis of the primary and secondary efficacy endpoints and the interim analysis of the safety and other endpoints.

SCHEDULE OF ACTIVITIES

The Schedule of Activities (SoA) outlines each visit and procedure of this protocol. Section 6 STUDY PROCEDURES and Section 7 STUDY ASSESSMENTS will provide detailed information required for compliance with the protocol. The investigator may schedule visits (unplanned visits) in addition to those listed in the SoA table, in order to conduct necessary evaluations or assessments required to protect the safety of the subject.

**Visits**	**Screening**	**Visit 1**	**Visit 2**	**Visit 3**	**Visit 4**	**Visit 5**	**Visit 6**	**Visit 7**
**Time points**	**< 24 hours[1]**	**Enrollment/Baseline0 h, dosing**	**12 hours after dosing**	**3 days after dosing**	**7 days after dosing**	**28 days after dosing**	**90 days after dosing**	**105 days after dosing/Early Withdrawal**
**Time windows**			**± 30 mins**	**± 4 hours**	**± 1 day**	**± 2 days**	**± 5 days**	**+7 days**
Informed consent	x							
Inclusion/exclusion criteria	x							
Tetanus vaccination history	x							
Collection of medical history/treatment history	x							
Randomization[2]	x							
Demographics	x							
Weight/height	x							
Vital signs[3]	x	x	x	x	x	x	x	
Physical examination	x				x	x	x	
12-lead ECG	x				x	x	x	
Serum pregnancy test[4]	x					x	x	

Visits	Screening	Visit 1	Visit 2	Visit 3	Visit 4	Visit 5	Visit 6	Visit 7
Time points	< 24 hours[1]	Enrollment/Baseline0 h, dosing	12 hours after dosing	3 days after dosing	7 days after dosing	28 days after dosing	90 days after dosing	105 days after dosing/Early Withdrawal
Time windows			± 30 mins	± 4 hours	± 1 day	± 2 days	± 5 days	+7 days
Laboratory tests (including hematology, clinical chemistry, coagulation, and urinalysis)[5]	x				x	x	x	
Study drug administration		x						
Adsorbed tetanus vaccine administration[6]		x						
Blood collection for anti-tetanus neutralizing antibody test[7]		x	x	x	x	x	x	
Blood collection for PK[7]		x	x	x	x	x	x	
Blood collection for immunogenicity[7]		x			x	x	x	
Collection of prior/concomitant medications	x	x	x	x	x	x	x	x
Observation after study drug administration[8]		x						
Assessment of injection site[9]		x	x	x				
Collection of tetanus occurrence		x	x	x	x	x	x	x
Collection of AEs[10]		x	x	x	x	x	x	x

1. The time from the injury to study drug administration should be less than 24 hours.

2. Randomization will be stratified by with or without concomitant administration of adsorbed tetanus vaccine. The proportion of subjects with concomitant adsorbed tetanus vaccine administration will be approximately 10%.
3. If the screening vital sign measurement is performed within 2 hours prior to dosing, the pre-dose baseline vital signs are not required to be repeated.
4. Blood pregnancy test is only for women of childbearing potential. The test will be performed at screening, and 28 and 90 days after dosing.
5. The details of laboratory tests can be found at Section 7.2.4.
6. The proportion of subjects (TNM002 or HTIG group) with concomitant administration of adsorbed tetanus vaccine will be approximately 10%. The vaccine will be provided by study sites qualified to administer tetanus vaccines.
7. Serum samples should be collected for PK, anti-tetanus neutralizing antibody, and immunogenicity tests.
8. Participants should be observed in study site not less than 30 mins after study drug dosing.
9. An injection site reaction assessment should be performed 30 (± 5) minutes, 12 hours (± 30 mins) and 3 days (± 4 hours) after IM injection of the study drug.
10. AEs will be collected from study drug dosing to end of study.

1. INTRODUCTION

TNM002 Injection is a recombinant fully human native mAb against tetanus toxin and is currently under development for indication of prophylaxis against tetanus.

1.1. Background

1.1.1. Tetanus

Tetanus is an acute infectious disease characterized by muscular rigidity and spasms caused by *Clostridium tetani*. The bacterium invades the human body through skin or mucosal wounds, thrives in an anaerobic environment, and produces exotoxin that attacks motor neurons of the nervous system[1]. The incubation period of tetanus can range from 1 day to several months after injury, but the disease typically manifests between 3 and 21 days after infection, with a median onset of 7 days[2].

The global mortality rate of tetanus is approximately 30% to 50%, and for severe cases, the mortality rate can reach close to 100% without medical intervention, particularly among the elderly and infants[3]. Thus, tetanus is a serious and potentially life-threatening disease.

1.1.2. Prophylaxis Against Tetanus

After injury, wound treatment and use of tetanus immunizations are crucial for preventing tetanus infection. Strategies for preventing tetanus include active immunization (using tetanus vaccine) and passive immunization (using anti-tetanus immunoglobulin).

According to the clinical guidelines[1,3], passive immunization, along with active immunization, is recommended for patients with unclean or contaminated wounds and having incomplete or unknown tetanus vaccination history. Long-term protection can be obtained after full vaccination schedule, and the protective effect can be maintained for 5 to 10 years. However, active vaccination is characterized by a slow onset of action, and antibodies generally reach the protective titer after about 2 weeks of injection. The incubation period of tetanus usually varies between 3 and 21 days after infection, with a median of 7 days. Therefore, passive immunizing agents play an important role in the prophylaxis of tetanus after injury in the above-mentioned population.

The passive immunization against tetanus mainly refers to injecting exogenous antibodies into the body to neutralize the toxins, thus granting immediate immunity, and offering short-term urgent protection against tetanus. The existing tetanus passive immunizing agents in China include human tetanus immunoglobulin (HTIG), equine tetanus antitoxin (TAT) and equine anti-tetanus immunoglobulin $F(ab')_2$. At present, the most widely used TAT is an antitoxin immunoglobulin preparation obtained from equine serum. However the rate of adverse reactions for TAT is high, and the rate of allergic reactions can reach up to 5%-30%, mainly presenting as anaphylactic shock and serum sickness[4], thus a "skin test" is required before adminstration. Due to its high incidence of adverse reactions, the TAT has been banned in developed countries since the 1960s[5], and the World Health Organization (WHO) has also removed it from the Essential Medicines List. In addition, the protection duration of TAT/$F(ab')_2$ is short, typically only lasting 10 days. Currently, HTIG is the preferred passive immunizing agent for tetanus in China and the rest of the world. However, due to the limited

supply of human plasma, its production cannot meet the medical demand, and its supply in China has been in a state of shortage and fluctuation for a long time. As a blood product, there is still a potential risk of transmitting known (e.g., acquired immune deficiency syndrome [AIDS], viral hepatitis) and unknown infectious diseases.

Based on the above unmet medical needs, it is urgent to develop a new passive immunizing agents with high safety, good efficacy and reliable quality as well as suitable for large-scale production.

1.2. Introduction of Investigational Drug

1.2.1. General Information of Investigational Drug

TNM002 Injection (hereinafter referred to as TNM002) is developed by Zhuhai Trinomab Pharmaceutical Co., Ltd. as a first-in-class drug. Its active ingredient is a recombinant native human monoclonal antibody (mAb) against tetanus toxin. TNM002 is an IgG1 mAb and consists of two identical light chains and two identical heavy chains which are linked by disulphide bonds. There are 1324 amino acids in total, and the theoretical molecular weight (MW) of the complete deglycosylated protein is 144.2 kDa. TNM002 can bind to specific surface of toxin protein through ionic bonds, and effectively prevents the toxin from binding to gangliosides on the surface of nerve cells.

1.2.2. Nonclinical Studies

The completed nonclinical studies with TNM002 include an in vitro pharmacodynamic (PD) study, an in vivo PD study in mice, human and cynomolgus monkeys tissue cross-reactivity studies, a safety pharmacology study in cynomolgus monkeys, a single-dose pharmacokinetic (PK) study in cynomolgus monkeys, an acute toxicity study in cynomolgus monkeys, a repeat-dose toxicity study in cynomolgus monkeys, a repeat-dose immunogenicity study in cynomolgus monkeys, a local tolerance study in cynomolgus monkeys, and an in vitro hemolysis study with rabbit erythrocytes. Additionally, a study on the co-administered of TNM002 with tetanus vaccine to evaluate antibody titers in mice, as well as an interaction study of TNM002 co-administered with tetanus vaccine in mice, have been completed.

The in vitro PD study showed that the relative binding activity and affinity of TNM002 to antigen Ag002(recombinant tetanus toxin [A + B fragment] protein) and tetanus toxin were high, and the binding activity of the two batches of the investigational drug to Ag002 and tetanus toxin was the same.

In the in vivo PD study, the preventive effect of TNM002 was assessed in an ICR challenge model of tetanus toxin-treated mice. Mice were given 1 to 100 IU/kg tetanus immunoglobulin (TIG) or 1 to 120 μg/kg TNM002 by intramuscular (IM) injection, followed by challenge with 10 times the median lethal dose (LD_{50}, 372.7 μg/kg) of tetanus toxin. The toxic manifestations and survival of mice were observed and recorded. The results showed that 1 μg/kg, 3.75 μg/kg, and 30 μg/kg TNM002 could achieve 50% survival protection,100% survival protection and complete disease-free protection in mice challenged with tetanus toxin, respectively.

In order to further evaluate the international unit titer of TAT corresponding to TNM002, the TAT titer assay recommended in Chinese Pharmacopoeia (in vivo neutralization method in mice)

was performed in the National Institutes for Food and Drug Control. The results showed that TNM002 (100 mg/5 mL/vial) had a strong anti-tetanus toxin effect and the potency was estimated at 7000 IU/mL (equivalent to 0.35 IU/μg) for Good Manufacturing Practice (GMP) batches.

In the acute toxicity study, no abnormalities were reported in animals and no mortality was observed throughout the study. There were no notable changes in clinical signs, body weights, food consumption, clinical pathology (hematology, clinical chemistry, coagulation, urinalysis), gross (necropsy) evaluations, and histopathological evaluations. The maximum tolerated dose (MTD) of TNM002 was considered to be 62.2 mg/kg/week.

In the repeat-dose toxicity study, TNM002 was well tolerated by IM injection at the doses of 3, 10 or 31.1 mg/kg/dose once a week (a total of three doses). There were no mortalities. No clinical adverse effects or toxicologically significant changes were noted in body weight, food consumption, ophthalmology, body temperature, safety pharmacology (ECG, blood pressure, heart rate, respiratory and neurological examinations), clinical pathology (hematology, coagulation, clinical chemistry, and urinalysis), organ weights, and macroscopic examinations. The treatment-related and investigational product-related microscopic changes were limited to the injection site. The treatment-related abnormalities were typical changes expected from injection trauma, and reported in both treatment and control groups. In the injection site irritation study, no investigational product-related injection site changes were observed up to the dose level of 31.1 mg/kg/day. Therefore, under the conditions of this study, the no observed adverse effect level (NOAEL) of TNM002 was considered to be 31.1 mg/kg/dose in males and females.

The results of the immunogenicity study showed TNM002 had a very low immunogenicity. In the in vitro hemolysis study, no hemolytic toxicity was observed with TNM002DS (test article) or TNM002 tox control buffer (control article). In the tissue cross-reactivity study, no positive staining of Biotin-TNM002 was observed in any human or cynomolgus monkey tissues.

In addition, two in vivo studies of TNM002 co-administered with tetanus vaccine have been completed: the first is a study on the co-administered of TNM002 with tetanus vaccine to evaluate antibody titers in mice, and the second is an interaction study of antibody co-administered with vaccine, including safety, toxicokinetic, immunogenicity and immunotoxicity investigations.

In the effect study of antibody co-administered with vaccine on TNM002 titer, 180 ICR mice were randomized into 30 groups with 6 mice each. The test groups included toxin control group, TNM002 group, TIG (human tetanus immunoglobulin standard) group, vaccine control group, TNM002 + vaccine group, and TIG + vaccine group. Seven dose cohorts were respectively set in the antibody and TIG groups. The corresponding vaccine was administered according to the group design, and challenge was performed 24 h after dosing (challenge dose: 10 times LD_{50}). In the combination group, the vaccine was administered at the dose of 0.667 IU/kg (converted based in the adult immunization standard dose of 40 IU/60 kg). The lowest non-fatal dose was 7.5 μg/kg in the TNM002 group, 15 μg/kg in the TNM002 + vaccine group, 2.5 IU/kg in the TIG group and 2.5 IU/kg in the TIG + vaccine group, respectively. The lowest non-morbidity

dose was 15 μg/kg in the TNM002 group, 15 μg/kg in the TNM002 + vaccine group, 5 IU/kg in the TIG group and 10 IU/kg in the TIG + vaccine group, respectively. The TNM002 antibody had a significant protective effect on tetanus toxin challenge in mice. In addition, the immunization of this antibody co-administered with the vaccine showed no significant effect on the antibody titer.

In the interaction safety study of TNM002 co-administered with tetanus vaccine, in order to ensure that antibodies could be developed in mice, the dose of adsorbed tetanus vaccine was 5 times the human dose, the dose in TNM002 low-dose group was 5 times the intended clinical dose (100 μg/ kg), and the dose in TNM002 high-dose group was 25 times the intended human dose. The results showed that when 500 and 2500 μg/kg TNM002 were given by a single IM injection, and 500 and 2500 μg/kg TNM002 (a single IM injection) co-administered with 0.216 IU/animal adsorbed tetanus vaccine (2 IM injections) were given within 4 weeks, no test article-related mortality was observed in ICR mice after 4-week recovery. No test article-related significant abnormalities were observed in clinical observations, body weight, food consumption, hematology, coagulation, clinical chemistry, gross anatomical observations, organ weights and coefficients, as well as pathological examinations. The NOAEL was 2500 μg/kg. This study was conducted under GLP conditions.

The toxicokinetic and immunogenicity study with TNM002 + tetanus vaccine showed that TNM002 exposure (C_{max} and AUC_{last}) increased with increasing dose in male and female mice in the dose range of 500-2500 μg/kg, with no significant gender difference. The coadministration of 0.216 IU/animal adsorbed tetanus vaccine had no significant effect on TNM002 exposure at the same dose. The antibody induced by the adsorbed tetanus vaccine was first detected on D15. The antibody titers increased first and then decreased over time. After the second dose, the antibody titers increased sharply on D36 and decreased on D57. After coadministration of 500 and 2500 μg/kg TNM002, the antibody induced by adsorbed tetanus vaccine was first detected on D8, and the titers were higher than that of the adsorbed tetanus vaccine monotherapy group, suggesting that TNM002 could induce adsorbed tetanus vaccine to develop antibody earlier in mice and increase the antibody titer. But with the increase of TNM002 dose, there was no significant difference. These data indicated that the combination of TNM002 and adsorbed tetanus vaccine had no negative effect on antibody induced by adsorbed tetanus vaccine.

The anti-TNM002 antibodies were detected in the TNM002 500 μg/kg, TNM002 2500 μg/kg and 500 and 2500 μg/kg combination groups, suggesting that TNM002 had certain immunogenicity in ICR mice. The antibody titer and positive rate in the TNM002 group tended to increase with the dose. The combination of TNM002 and adsorbed tetanus vaccine could significantly increase the positive rate and titer of anti-TNM002 antibodies.

In addition, the immunotoxicity study of TNM002 co-administered with tetanus vaccine showed that no test article-related significant abnormal changes were observed in cytokines such as interleukin (IL)-2, IL-4, IL-5, IL-6, tumor necrosis factor-α (TNF-α), interferon-γ (IFN-γ) in any group at 6 h and 24 h after the first and last dose.

1.2.3. Previous Clinical Studies

Two phase 1 clinical studies have been completed during the clinical development of TNM002. A phase 2 clinical study is currently ongoing and the primary analysis has been completed. Based on the data from the completed phase 1 clinical studies, the population PK (PopPK) and PK/PD analysis has also been conducted.

<u>Phase 1 Clinical Study with TNM002 in Australia (TNM002-P1-AU01)</u>

This study entitled "A Randomized, Double-Blind, Placebo-Controlled, Dose-Escalation Study to Evaluate the Safety, Tolerability, Pharmacokinetics and Pharmacodynamics following Intramuscular Administration of a Single Dose of TNM002 in Healthy Subjects" was the first-in-human (FIH) clinical study with TNM002.

A total of 32 subjects were enrolled in the study and sequentially enrolled into 4 dose cohorts (10, 35, 100, and 250 μg/kg). The mean age of the 32 subjects participating in this study was 30.0 years (range: 19 to 55 years), with 16 males and 16 females. The majority of subjects were Caucasian, accounting for 43.8% (14 subjects) of the total subjects, and 25% of the subjects (8 subjects) were Asian. The mean weight of the subjects was 71.2 kg (range: 45.7 to 112.8 kg), and the weights were generally balanced among cohorts.

The safety results showed that TNM002 Injection was safe and well tolerated in healthy adults. The incidence of treatment emergent adverse events (TEAEs) in the TNM002 cohorts ([redacted] was comparable to that in the placebo group ([redacted]). All TEAEs were Common Terminology Criteria for Adverse Events (CTCAE) Grade 1 or 2 in severity. During the study, 1 serious adverse event (SAE) [redacted]) occurred in a subject who received placebo. No subjects died during the study and no subjects withdrew from the study prematurely due to AEs.

The most common TEAEs in the TNM002 cohorts were headache ([redacted], upper respiratory tract infection ([redacted]), and injection site pain ([redacted]). The incidences of headache and upper respiratory tract infection were comparable to those in the placebo group. The injection site pain all occurred in the 250 μg/kg cohort and none was reported in the placebo group. In this study, a total of 3 subjects reported drug-related TEAEs ([redacted]). These 3 subjects were all from the 250 μg/kg dose cohort, and these TEAEs were all CTCAE Grade 1 in severity and resolved spontaneously.

PK and PD results showed that serum TNM002 exposure increased linearly with increasing dose, and anti-tetanus neutralizing antibody titers also increased with increasing dose. The median time to maximum plasma concentration (T_{max}) of TNM002 was [redacted] and the elimination half-life ($t_{1/2}$) was [redacted] At 24 hours after IM injection of TNM002, no subject in the 10 μg/kg dose cohort achieved anti-tetanus neutralizing antibody titers ≥ 0.01 IU/mL, while in the 35, 100, and 250 μg/kg dose cohorts, the percentage of subjects with anti-tetanus neutralizing antibody titers ≥ 0.01 IU/mL was [redacted], respectively. At 24 hours, the anti-tetanus neutralizing antibody geometric mean titers (GMTs) were [redacted] in the above four dose cohorts, respectively.

On Day 64 after IM injection of TNM002, [redacted] subject in the 10 µg/kg dose cohort achieved anti-tetanus neutralizing antibody titers ≥ 0.01 IU/mL, while in the 35, 100, and 250 µg/kg dose groups, the percentage of subjects with anti-tetanus neutralizing antibody titers ≥ 0.01 IU/mL was [redacted] On Day 64, the anti-tetanus neutralizing antibody GMTs were [redacted] in the above four dose cohorts, respectively.

In this study, ADA testing was performed pre-dose (baseline), on Days 6, 15, 29, 43, 64, 85, and 106, respectively. All subjects had negative ADA test results at all time points, indicating that TNM002 Injection had a very low immunogenicity.

Phase 1 Clinical Study with TNM002 in China (TNM002-P1-CH01)

This study entitled "A Randomized, Double-Blind, Placebo-Controlled, Dose-Escalation Phase 1 Study to Evaluate the Safety, Tolerability, Pharmacokinetics and Pharmacodynamics following Intramuscular Administration of a Single Dose of TNM002 in Healthy Subjects" was conducted in Chinese healthy subjects. A total of 28 subjects were enrolled in this study, including 12 subjects in the 35 µg/kg cohort and 8 subjects in the 100 and 250 µg/kg cohorts, respectively (TNM002: placebo = 6: 2).

Sixteen (57.1%) subjects were male, and 12 (42.9%) subjects were female. The mean age of the subjects was 29.7 years (range: 20 to 45 years). The mean weight was 62.61 kg (range: 45.0 to 83.6 kg) and mean body mass index was 22.92 kg/m^2 (range: 19.1 to 26.0 kg/m^2). The demographic characteristics of the subjects were not significantly different between the TNM002 cohorts and placebo group.

A total of [redacted] subjects reported TEAEs. Of these, a total of [redacted] in the TNM002 cohorts [redacted] and [redacted] in the placebo group [redacted] reported TEAEs. No study drug-related TEAEs occurred during the study.

All reported TEAEs were CTCAE Grade 1 or 2 in severity. A total of 1 SAE was reported in 1 subject (TNM002 35 µg/kg cohort). The subject underwent induced abortion due to pregnancy, and this SAE was judged by the investigator to be unrelated to the study drug. There were no AEs leading to death, no injection-related AEs, and no AEs leading to withdrawal from the study.

PK and PD results showed that serum TNM002 exposure generally increased linearly with increasing dose, and anti-tetanus neutralizing antibody titers also increased with increasing dose. The primary PK parameters in this study were similar to those in phase 1 study in Australia: the median T_{max} was [redacted] and $t_{1/2}$ was [redacted].

In the 100 µg/kg dose cohort, [redacted] of subjects had an increase of neutralizing antibody titers (Δ titers) ≥ 0.01 IU/mL from baseline at 24 h post-dose and all subjects had an increase of Δ titers from baseline ≥ 0.01 IU/mL at 48 h post-dose. Until 1512 h post-dose (Day 63), the neutralizing antibody titers remained above this cut point in all subjects. The geometric mean of Δ titers was [redacted] at 48 h post-dose, which was significantly higher than 0.01 IU/mL, the lowest protective titer of tetanus neutralizing antibody. The geometric mean of Δ titers was [redacted] at 1512 h (Day 63) post-dose.

In the 250 µg/kg dose cohort, all subjects had an increase of Δ titers ≥ 0.01 IU/mL from baseline

at 6 h post-dose. Until the last visit time point, 2520 h post-dose (Day 105), the neutralizing antibody titers remained above this cut point in all subjects. The geometric mean of Δ titers was [redacted] at 6 h post-dose, and [redacted] at 2520 h (Day 105) post-dose, respectively.

PopPK and PK/PD Analysis Based on PK and PD Data from Phase 1 Clinical Studies in Australia and China

The PopPK and PK/PD analysis was conducted based on PK and PD data from the phase 1 clinical studies in Australia and China. Based on the established PopPK model and PK/PD model, 1000 subjects of each sex were randomly generated within the baseline weight range derived from 60 subjects in phase 1 clinical studies in China and Australia. The dosing regimens were 2 mg, 5 mg, 6 mg, 7 mg, 8 mg, 9 mg, 10 mg, 15 mg, and 20 mg by IM injection at fixed doses. The results showed that the percentage of subjects with neutralizing antibody titers ≥ 0.01 IU/mL at 24 h post-dose increased with increasing dose. The percentage of subjects with neutralizing antibody titers ≥ 0.01 IU/mL basically reached the maximum effect level at 9 mg and 10 mg. After a single IM injection of TNM002 with a fixed dose of 10 mg, the tetanus neutralizing antibody titers in subjects increased rapidly, and the tetanus neutralizing antibody titers in more than 99% subjects was ≥0.01 IU/mL at 24 hours post-dose, which could provide rapid and effective protection for subjects.

Phase 2 Clinical Study with TNM002 (TNM002-P2-CH01)

This study is a multicenter, randomized, double-blind, parallel-controlled, dose-finding phase 2 study to compare the anti-tetanus neutralizing antibody titers and safety of TNM002 Injection with human tetanus immunoglobulin or placebo following a single intramuscular injection in Chinese adult subjects. The primary objective of this study is to compare the anti-tetanus neutralizing antibody titers of TNM002 Injection with HTIG following a single IM injection in Chinese adult subjects. The secondary objectives of this study are to compare the safety and tolerability of TNM002 Injection with HTIG and placebo following a single IM injection, to assess the PK properties of TNM002 Injection in Chinese adult subjects and to assess the immunogenicity of TNM002 Injection in Chinese adult subjects. In this study, 240 Chinese adult subjects are enrolled as planned and randomized in a ratio of 2:2:1:2:1 to TNM002 Injection 5 mg, TNM002 Injection 10 mg, TNM002 Injection 15 mg, HTIG 250 IU, and placebo groups.

As of the cut-off date for the primary analysis (June 25, 2022), no subjects withdrew from the study due to AEs and no volunteer died. One volunteer received the study drug on May 20, 2022 and was hit by a car while riding an electric bicycle on June 10, 2022. Then he/she was sent to another hospital for inpatient treatment. According to the diagnosis in the hospital, the volunteer experienced an SAE and the SAE was reported as "traumatic moderate craniocerebral injury: left frontotemporal subdural bleeding". His/her condition was stable after hospitalization and the volunteer was discharged from the hospital on June 20, 2022. The SAE was judged by the investigator to be unrelated to the study drug.

1.3. Objective and Rationale

Tetanus is a serious and potentially life-threatening disease. After injury, wound treatment and use of tetanus immunizations are crucial to prevent tetanus infection. Passive immunization is

required for patients with unclean or contaminated wounds and having incomplete or unknown tetanus immunization history. At present, the widely used TAT has problems such as high incidence of adverse reactions, need for skin test and short protection time, while the production capacity of HTIG as a blood product is limited and cannot meet the clinical needs. Therefore, it is urgent to develop a new passive immunizing agent with high safety, good efficacy and reliable quality as well as suitable for large-scale production.

Based on the results of completed phase 1 studies in Australian and China, and primary analysis results from ongoing phase 2 study, a proposed clinical dose of 10 mg is determined for further pivotal phase 3 study to compare the efficacy and safety of passive immunization with TNM002 Injection and human tetanus immunoglobulin as prophylaxis against tetanus, the result of which will be used to support the marketing application of TNM002.

1.4. Benefit/Risk Assessment

The mortality rate of the disease is approximately 30% to 50% worldwide, and the mortality rate for subjects with severe tetanus is close to 100% without medical intervention, especially in the elderly and infants[2]. Thus, tetanus is a serious and potentially life-threatening disease. Passive immunization is required for patients with unclean or contaminated wounds and having incomplete or unknown tetanus immunization history. At present, TAT has problems such as high incidence of adverse reactions, need for skin test and short protection time (usually only 10 days). For HTIG as a preferred product, the production capacity is limited; there is a potential risk of transmitting known (e.g., AIDS, viral hepatitis) and unknown infectious diseases; and the protection time is approximately 28 days.

Based on the unmet clinical needs, TNM002 Injection, a recombinant fully human native mAb against tetanus toxin, has potential to be a new tetanus passive immunizing agent with high safety, good efficacy and reliable quality as well as suitable for large-scale production which may bring more benefit to the clinical subjects. On the basis of phase 1 and phase 2 studies, this study is intended to further evaluate the efficacy and safety of the investigational drug and to confirm the potential benefit-risk relationship in the actual clinical subject population.

Currently, the comparator HTIG is the preferred passive immunizing agents for tetanus prophylaxis. It has been used for many years and shows a definite efficacy and low risk of adverse reactions such as allergy. Available clinical trial data of TNM002 Injection shows good safety and tolerability. The incidence of TEAEs is comparable to placebo, and no specific risks in human have been identified. Compared to HTIG 250 IU, TNM002 10 mg is expected to rapidly achieve protective level of anti-tetanus neutralizing antibody in a higher proportion of subjects, and to maintain a longer duration.

In addition, the study also adopts a series of settings to reduce the risk of enrolled subjects. This includes excluding subjects at high risk of potential allergic reactions or bleeding through exclusion criteria; performing TNM002 or HTIG injection as soon as possible on the basis of wound treatment in accordance with clinical practice; close observation after administration, and medical intervention when local injection reactions or allergic reactions occur. For female subjects of childbearing potential, negative serum pregnancy test at screening should be obtained and highly effective contraceptive methods should be used. During the study, a series

of risk control measures such as standard and intensive follow-up monitoring and close monitoring of the safety will also be adopted to minimize risks and protect the rights and interests of subjects.

More detailed information about the known and expected benefits and risks and reasonably expected adverse events (AEs) of TNM002 Injection may be found in the investigator's brochure (IB).

2. OBJECTIVES AND ENDPOINTS

Primary Objectives, Endpoints and Estimands:

Objectives	Endpoints	Estimands
• To compare the efficacy of TNM002 Injection 10 mg with HTIG 250 IU as prophylaxis against tetanus following a single IM injection	• The increase of anti-tetanus neutralizing antibody titers from baseline at 12 hours after receipt of study drug	In all randomized subjects who have received study drug, the proportion of subjects with an increase of anti-tetanus neutralizing antibody titers (Δ titers) ⩾ 0.01 IU/mL from baseline at 12 hours after receipt of study drug.<br>Subjects who are diagnosed with tetanus within 12 hours after receipt of study drug, or have received additional passive immunizing agent due to the original wound, or have experienced AE leading to study discontinuation within 12 hours after receipt of study drug, are considered to have a Δ titer < 0.01 IU/mL.

Secondary Efficacy Objectives, Endpoints and Estimands:

Objectives	Endpoints	Estimands
• To compare the clinical outcomes after administration of TNM002 10 mg with HTIG 250 IU as prophylaxis against tetanus following a single IM injection	• Tetanus protection rate within 28 days after receipt of study drug	• In all randomized subjects who have receive study drug: Tetanus protection rate (1 - tetanus incidence) within 28 days after receipt of study drug

Safety Objectives, Endpoints and Estimands:

Objectives	Endpoints	**Estimands**
• To compare the safety profile of TNM002 10 mg with HTIG 250 IU as prophylaxis against tetanus following a single IM injection	• AEs<br>• SAEs<br>• Laboratory tests, vital signs, physical examinations, and ECGs	In all randomized subjects who have received study drug and having at least one post-dose safety evaluation<br>• Incidence of AEs for up to 105 days after receipt of study drug<br>• Incidence of treatment related AEs for up to 105 days after receipt of study drug<br>• Incidence of SAEs for up to 105 days after receipt of study drug<br>• Summary statistics and shift tables (if appropriate) of laboratory tests, vital signs, physical examinations, and ECGs for up to 90 days after receipt of study drug

Tertiary Objectives, Endpoints and Estimands:

Objectives	Endpoints	**Estimands**
• To compare the protective antibody level of TNM002 10 mg with HTIG 250 IU as prophylaxis against tetanus at different time points following a single IM injection	• The increase of anti-tetanus neutralizing antibody titers from baseline at 3, 7, 28 and 90 days after receipt of study drug	In all randomized subjects who have received study drug, and haven’t received tetanus vaccine up to the respective time points:<br>• Proportion of subjects with Δ titers ≥ 0.01 IU/mL from baseline at 3, 7, 28 and 90 days after receipt of study drug, respectively<br>• Geometric mean of Δ titers from baseline at 12 hours, and 3, 7, 28 and 90 days after receipt of study drug, respectively<br>Subjects who are diagnosed with tetanus as of the respective time point, or have received additional passive immunizing agent due to the original wound, are considered to have an antibody titer of LLOQ/2 and a Δ titer < 0.01 IU/mL at that time point.
• To compare the long-term clinical outcomes after administration of TNM002 10 mg with HTIG 250 IU as prophylaxis against tetanus following a single IM injection	• Tetanus protection rate within 90 and 105 Days after receipt of study drug	• In all randomized subjects who have received study drug: Tetanus protection rate (1 - tetanus incidence) within 90 and 105 days after receipt of study drug, respectively

Objectives	Endpoints	**Estimands**
• To evaluate the pharmacokinetic (PK) properties of TNM002 in subjects requiring passive tetanus immunization	• Serum TNM002 concentrations in subjects following TNM002 administration	• In all randomized subjects who have received TNM002, having at least one post-dose evaluable PK concentration, and without major protocol deviations that affect PK concentrations of the respective time point: Serum TNM002 concentration at baseline, 12 hours, and 3, 7, 28 and 90 days after receipt of TNM002, respectively
• To evaluate the immunogenicity of TNM002 in subjects requiring passive tetanus immunization	• Anti-drug antibodies (ADA) in subjects following TNM002 administration	• In all randomized subjects who have received TNM002 and having post-dose immunogenicity data: ADA positive rate at 7, 28 and 90 days after receipt of TNM002, respectively

Note: The increase of anti-tetanus neutralizing antibody titers from baseline in the TNM002 group is defined as: the post-dose values by quantitative detection of TNM002 serum titer using the MSD platform (referred to as TNM002 PD bioanalysis method) at respective time points minus the pre-dose baseline values;

The increase of anti-tetanus neutralizing antibody titers from baseline in the HTIG group is defined as: the post-dose values at respective time points by quantitative detection of HTIG serum concentration using the chemiluminescence method (referred to as HTIG PD bioanalysis method) minus the pre-dose baseline values.

3. STUDY DESIGN

3.1. Overall Design

This is a phase 3, multicenter, randomized, double-blind, parallel-group, active-controlled study to compare the efficacy and safety of TNM002 injection (hereinafter referred to as TNM002) 10 mg and HTIG 250 IU as prophylaxis against tetanus in subjects with unclean or contaminated wounds caused by various injuries and who require passive tetanus immunization.

Approximately 675 subjects are planned to be enrolled in this study. These subjects will be randomized and receive a single IM gluteal injection of TNM002 10 mg (450 subjects) or HTIG 250 IU (225 subjects) in a 2:1 ratio. Randomization will be stratified by with or without concomitant administration of adsorbed tetanus vaccine. The proportion of subjects with concomitant adsorbed tetanus vaccine administration will be approximately 10%. The vaccine will be provided by study sites qualified to administer tetanus vaccines. It will be intramuscularly injected into deltoid muscle of upper arm according to the vaccine manufacture instructions.

The study personnel responsible for drug preparation and administration will be unblinded due to the apparent difference in the dosing volumes of TNM002 (0.5 mL) and HTIG (2.5 mL). During the study drug administration, measures should be taken to block subject’s line of sight, in order to ensure that the subject cannot observe any identifying information of the study drug. The injection time for all study drugs should be precisely 10 (± 1) seconds. The subjects,

investigators, and the other study personnels at the study sites will be blinded throughout the study.

Before and after study drug administration, wound treatment should be performed in accordance with the clinical standard procedure. Concomitant medicine or other treatment required for injury itself or comorbidities are permitted during the study.

Serum samples for anti-tetanus neutralizing antibody and PK will be collected from all subjects at pre-dose, 12 hours, and 3, 7, 28 and 90 days after dosing. Serum samples for immunogenicity tests will be collected from all subjects at pre-dose, and 7, 28 and 90 days after dosing. These tests will be performed at a central bioassay laboratory.

During the visits, subjects will also undergo safety assessments including collection of AEs, physical examinations, vital signs, 12-lead ECGs, and safety laboratory tests (hematology, clinical chemistry, coagulation, and urinalysis), and the tetanus occurrence will also be monitored. The last visit for this study is planned to be performed at 105 days after dosing and will be completed by telephone contact.

As the diagnosis of tetanus is mainly based on clinical symptoms and no reliable objective diagnostic criteria are available, a clinical adjudication committee consisting of clinical experts in the field of tetanus disease will be established. For subjects with tetanus-related symptoms, the final diagnosis will be made by experts in this committee after discussion.

The primary analysis is planned to be performed after the last enrolled subject has completed the visit at 28 days after dosing, in order to evaluate the efficacy and safety of the study drug. The analysis will be carried out by an independent unblinded team.

3.2. Rationale for the Design

3.2.1. Study Population and Comparator

TNM002 is being developed for passive tetanus immunizations and the intended indication is prophylaxis against tetanus. Therefore, this study is planned to enroll subjects with unclean or contaminated wounds caused by various injuries such as incised wound, burn, traffic wound, animal scratch and bite wounds who require passive immunization as prophylaxis against tetanus.

HTIG is the preferred passive immunizing agents by Chinese and internation clinical guidelines for tetanus prophylaxis. It can effectively prevent tetanus and has been widely used in clinical practice for a long time. A single IM injection of HTIG 250 IU is a routine dose for tetanus prophylaxis. Therefore, this study includes a test group and an active control group to assess the efficacy and safety of TNM002 as prophylaxis against tetanus in the target population. HTIG at a dose of 250 IU is selected as the active control.

3.2.2. Primary Endpoint

Immunity to tetanus is antibody-mediated. The mechanism of tetanus prophylaxis and treatment is neutralizing the toxin by neutralizing antibodies, which prevents the toxin from binding to nerve cells. The level of serum anti-tetanus neutralizing antibody is critical to occurrence of

tetanus after infection with *Clostridium tetani*. Therefore, it can be used as a surrogate endpoint to evaluate the efficacy for tetanus prophylaxis.

The incubation period of tetanus varies from 1 day to several months after injury, and the onset of the disease in most cases occurs between 3 days and 3 weeks after injury. A pooled analysis of 1191 non-neonatal cases in the United States from 1972 to 2001 showed that the median time from injury to tetanus onset was 7 days (range: 0 to 178 days). The time from injury to onset of symptoms was less than 2 days in approximately 10% of cases and more than 30 days in approximately 3% of cases[6,7]. To evaluate the protective effect of passive immunizing agents in prophylaxis against tetanus after injury, especially in terms of early prevention, the 12 h post-dose was selected as the time point for the evaluation of primary efficacy endpoint.

According to recommendations of WHO and general consensus, the minimum neutralizing antibody level for tetanus protection is 0.01 IU/mL[8]. Therefore, in this phase 3 confirmatory clinical trial, the primary endpoint is set as the proportion of subjects with an increase of anti-tetanus neutralizing antibody titers (Δ titers) ≥ 0.01 IU/mL from baseline at 12 hours after receipt of study drug. In addition, in order to comprehensively assess and compare the prophylactic efficacy of the investigational drug at multiple key time points during the tetanus incubation period, the levels of anti-tetanus neutralizing antibodies at 3, 7, 28, and 90 days after receipt of study drug will also be included as other endpoints.

3.2.3. Justification for Dose

The safety data from the primary analysis of the phase 2 study, as well as the safety data from the completed phase 1 studies in Australia and China, indicated that the 10 mg dose of TNM002 has good safety and tolerability. The overall incidence of TEAEs was similar to that of the placebo group or the HTIG group.

The preclinical study results for TNM002 indicated that [redacted]

The PD data from the primary analysis of the phase 2 study supports that, [redacted].

Although the incubation period of tetanus is mostly 3 days to 3 weeks after injury, literature reports suggest that approximately 10% of cases develop symptoms in less than 2 days after injury, and about 3% of cases after more than 30 days [6,7]. Considering the serious outcomes once tetanus onset, along with the heavy social and family burdens it may incur, passive immunization for tetanus should aim to achieve protective levels of neutralizing antibodies in as many participants as possible soon after injury, covering an extended duration to effectively protect even those with long incubation periods. In addition, there are documented cases in clinical practice where tetanus still occurred despite antibody levels being above the protective threshold. Therefore, it is necessary to maintain a higher levels of neutralizing antibody titers

in participants for an extended duration to provide more effective protection and benefit to them. It is anticipated that TNM002 10 mg dosage can rapidly provide higher levels of antibody titers in participants.

In conclusion, the safety and tolerability of TNM002 at a 10 mg dosage are favorable. Existing data provides justification for choosing TNM002 10 mg for the phase 3 trial, aiming to assess its superior efficacy compared to the current standard-of-care passive tetanus immunization with HTIG 250 IU.

3.2.4. Proportion of Participants Receiving Adsorbed Tetanus Vaccine Concomitantly

According to the *Guidelines for the use of post-traumatic tetanus vaccines and passive immune preparation*[1], for patients with unclean or contaminated wounds and having incomplete or uncertain tetanus vaccination history, tetanus vaccine is recommended to be concomitantly administrated with passive immunizing agent. This approach effectively prevents tetanus following the current injury while also could induce long-term (approximately 5-10 years) immunoprotection through vaccination. Therefore, a subset of subjects receiving tetanus vaccine concomitantly will be included in this study. However, due to the following reasons, the proportion of this subset of subjects was set at approximately 10%:

1) Currently, very few subjects receive tetanus vaccine concomitantly with passive immunization in clinical practice in China. Our survey results indicate that approximately ≤ 10% of hospitals can provide tetanus vaccine for adults.

2) After receiving tetanus vaccination, subjects may develop tetanus antibodies within 3-7 days[9]. If TNM002 or HTIG is given simultaneously with the vaccine, antibody levels at 3 days after dosing actually reflect the combined effect of passive (TNM002 or HTIG) and active immunizations (vaccine). However, since each subject's tetanus immunization history varies, the individual differences in antibody levels induced after the administration of tetanus vaccine for adults are significant. The combined antibody levels may not accurately reflect the efficacy comparison between the two passive immunizing agents (TNM002 and HTIG). Therefore, including a high proportion of subjects with concomitant administration of vaccine in phase 3 trial would make it difficult to achieve the study objective of compare the efficacy of TNM002 and HTIG accurately and comprehensively at key time points during the tetanus incubation period (such as 3, 7, 28, 90 days).

3) The evaluation of tetanus passive immunizing agents mainly focuses on their effectiveness in preventing tetanus following the present injury, rather than providing long-term protection against tetanus for potential future injuries.

In this clinical trial, the proportion of subjects with concomitant tetanus vaccine administration was set at approximately 10% to minimize the impact on the comprehensive and accurate assessment of TNM002 and HTIG while in consistent with current clinical practices. Participants receiving concomitant administration of the tetanus vaccine will be recruited and enrolled at sites qualified to administer tetanus vaccines for adults.

3.2.5. Stratified Randomization

To maintain balance in the use of tetanus vaccine across treatment groups, stratified randomization will be implemented based on whether tetanus vaccine will be concomitant administered or not. The proportion of subjects with concomitant tetanus vaccine administration will not exceed approximately 10%.

3.2.6. Study Drug Administration and Blinding

The majority of tetanus incubation periods range from 3 days to 3 weeks after injury, so passive immunizing agents should be administered as early as possible. In clinical trials setting, in order to achieve optimal conditions and results, the timing of post-exposure passive immunization is defined. In this trial, the administration of study drug is required within 24 hours after the injury.

In this trial, there are differences in the appearance and volume of administration between the test durg TNM002 and the active control drug HTIG. To maintain blinding, unblinded personnel will be designated for drug preparation and administration. Additionally, during administration, each participant should receive the injection in an independent physical spaces to ensure blinding. Measures will also be taken before and after administration to ensure that participants cannot observe any identifying information about the injected drug (such as the outer packaging, shape of the syringe, volume of the drug in the syringe, and color of the drug). The drug preparation and administration personnel are prohibited from discussing any information related to the study drug with investigators or participants.

4. STUDY POPULATION

4.1. Inclusion Criteria

Participants must meet all of the following inclusion criteria to be eligible for the study:

1) Voluntary signing of the informed consent form (ICF);
2) Male or female subjects aged ≥ 18 years;
3) Participants with unclean or contaminated wounds caused by various injuries such as incised wound, burn, traffic wound, animal scratch and bite wounds (Appendix 1) who require passive immunization as prophylaxis against tetanus;
4) The time from above injury to study drug administration is expected less than 24 hours;
5) Women of childbearing potential and at risk of pregnancy and males who are sexually active must agree to use at least one of the following highly effective contraceptive methods throughout the study and for 150 days after dosing, including:

- Intrauterine device (IUD);
- Oral, injectable, or implantable hormonal contraceptives;
- Barrier contraception + spermicide;
- Surgical contraceptive methods (e.g., vasectomy, salpingectomy, hysterectomy, etc.);

Women of no childbearing potential will be authorized to participate in this study if at least one of the following criteria are met:

- Documented hysterectomy and/or bilateral oophorectomy;
- Medically confirmed ovarian function failure;
- Postmenopausal, defined as follows: cessation of regular menses for at least 12 consecutive months with no alternative pathological or physiological cause.

4.2. Exclusion Criteria

Participants who meet any of the following criteria will be excluded from the study:

1) All wounds are clean (Appendix 1);
2) Need to use HTIG 500 IU due to severe or serious contaminated wound, as judged by the investigator;
3) Suspect or diagnosed as tetanus;
4) Fever (body temperature ≥ 38 °C) within 3 days prior to dosing;
5) Prior vaccination history of ≥ 3 doses of tetanus toxoid or tetanus toxoid- containing vaccine;
6) Previously diagnosed with tetanus infection;
7) Previously diagnosed as IgA deficiency with anti-IgA antibodies;
8) Participants have received immunoglobulins, blood or blood products within 6 months prior to dosing; subjects plan to receive live viral vaccines within 3 months after dosing;
9) Participants with hemorrhagic factors, or at high risk of bleeding, including congenital hemorrhagic disorders (e.g., hemophilia) or any clinically significant active bleeding, or abnormal platelet function, or prothrombin time (PT) > 3 seconds above the ULN by coagulation tests, or platelet count < 100×10^9/L (except for bleeding associated with injury such as incised wound, burn, and traffic wound);
10) Have received anticoagulants within 3 weeks prior to dosing;
11) Pregnant women or women who plan to become pregnant during the study and nursing mothers;
12) Current alcohol abuse, drug abuse or drug addiction;
13) Known or suspected allergy to the investigational product or its excipients, or have a history of allergy to human immunoglobulin products or other therapeutic monoclonal immunoglobulins;
14) Have participated in other clinical studies with investigational drugs or devices within 3 months or 5 times the half-life of the drugs (whichever is longer) prior to dosing, except for observational or non-interventional clinical studies;

15) Investigator site staff or Sponsor employees directly involved in the conduct of the study, site staff otherwise supervised by the investigator, and their respective family members;

16) Participants who are ineligible for the study due to other factors judged by the investigator.

5. STUDY DRUG

Study drug is defined as any investigational drug(s) or marketed product(s) administered to the subjects according to the study protocol. In this report, the term study drug refers to TNM002 and/or HTIG, and the term test product refers to TNM002. Adsorbed tetanus vaccine is a non-study drug. The package inserts for the comparator HTIG and adsorbed tetanus vaccine are detailed in Annex 1 and Annex 2.

5.1. Study Drug Information

	**Test Product**	**Comparator**
Drug Name	TNM002 Injection	Human tetanus immunoglobulin
Dosage Form	Injection, solution	Injection, solution
Strength	10 mg/0.5 mL/vial	250 IU/2.5 mL/vial
Composition	Active ingredient:<br>Recombinant native human mAb against tetanus toxin	Active ingredient:<br>Human tetanus immunoglobulin
	Excipients:<br>Histidine/histidine hydrochloride, 20 mM; sucrose, 4%; polysorbate 80, 0.02%; 70 mM sodium chloride	Excipients:<br>Glucose, polysorbate 80, sodium chloride, glycine, thimerosal, sterile water of injection
Manufacturer	Zhuhai Trinomab Pharmaceutical Co., Ltd.	Hualan Biological Chongqing Co., Ltd.
Storage Conditions	Stored and transported at 2-8 °C, protected from light	Stored and transported at 2-8 °C, protected from light
Dose	10 mg (0.5 mL)	250 IU (2.5 mL)
Route	IM gluteal injection	IM gluteal injection

5.2. Drug Dispensing and Preparation

After screening, the study drug will be dispensed using an Interactive Response Technology (IRT) drug management system.

All study drugs will be prepared by an unblinded pharmacist and/or study nurse, with corresponding volume drawn according to the randomization list. Upon completion of preparation, the study drug will be administered by the study nurse.

5.3. Administration

Each subject will receive one dose of study drug at the visit specified in the study procedures. All administrations will be carried out by the study nurse. The study nurse will inject 0.5 mL or 2.5 mL of the assigned study drug into the participant's gluteal muscle. During administration, each participant will receive the injection in an independent physical space. Measures will be taken to block the participant's view before and after administration to ensure they cannot see any identifying information about the injected product, such as the outer packaging, shape of the syringe, volume of the drug in the syringe, and the color of the drug. The injection time for all study drugs should be precisely 10 (± 1) seconds. See the Pharmacy Manual for details.

5.4. Handling, Storage, and Accountability

The investigator or designee must confirm appropriate temperature conditions have been maintained during transit for all study drugs received and any discrepancies are reported and resolved before use of the study drug.

All study drugs must be stored in their original containers and must be stored in a secure, environmentally controlled, and monitored (manual or automated recording) area in accordance with the labeled storage conditions with access limited to the investigator and authorized site staff. At a minimum, daily minimum and maximum temperatures for all site storage locations must be documented and available upon request. Data for nonworking days must indicate the minimum and maximum temperature since previously documented for all site storage locations upon return to business.

Any excursions from the labeled storage conditions should be reported to the Sponsor Trinomab upon discovery along with any actions taken. The site should actively pursue options for returning the study drug to the storage conditions described in the labeling, as soon as possible. Once an excursion is identified, the study drug must be quarantined and not used until Trinomab provides permission to use the study drug. It will not be considered a protocol deviation if Trinomab approves the use of the study drug after the temperature excursion. Use of the study drug prior to Trinomab approval will be considered a protocol deviation. Specific details regarding the definition of an excursion and information the site should report for each excursion will be provided in the Pharmacy Manual.

The investigator or the head of the medical institution (where applicable) will be responsible for study drug accountability, reconciliation, and record maintenance (ie, receipt, reconciliation, and final disposition records). All study drugs will be accounted for using an accountability form/record.

The Sponsor or designee will provide guidance on the destruction of unused study drug (eg, at the site). If destruction is authorized to take place at the investigator site, the investigator must ensure that the materials are destroyed in compliance with applicable environmental regulations, institutional policy, and any special instructions provided by Trinomab, and all destruction must be adequately documented.

Additional details about accountability, storage, destruction, and excursion reporting can be found in the Pharmacy Manual.

5.5. Randomization and blinding

5.5.1. Randomization

Participants participating in this study will be randomized and receive TNM002 or HTIG in a 2:1 ratio. A stratified block randomization approach will be used. Randomization will be stratified by with or without concomitant administration of tetanus vaccine. The proportion of subjects with concomitant tetanus vaccine administration will be approximately 10%.

The randomization list will be generated by an independent statistician who is not directly involved in the study via a computer program. The treatment assignment will be randomized in the IRT system. The assigned randomization number cannot be used for another subject.

IRT Reference Manual will provide further details on the use of the IRT system.

5.5.2. Blinding

The investigator, Sponsor, and subject will remain blinded to the study drug assigned to the subject throughout the study. The appropriate blind maintenance plan will be prepared. In order to maintain the blinded state, the drug preparation and administration will be performed by an unblinded pharmacist and/or study nurse. The unblinded personnel will be trained according to the blind maintenance plan prior to the start of the study. During the dosing, ensure that each subject is administered by injection in an independent physical space. Measures will be taken to block the participant's view before and after administration to ensure they cannot see any identifying information about the injected product, such as the outer packaging, shape of the syringe, volume of the drug in the syringe, and the color of the drug. See the Pharmacy Manual for details. The pharmacist and/or study nurse should avoid discussing any information related to the study drug with the investigator and the subject, such as injection volume and injection time.

5.5.3. Emergency Unblinding

Procedures will be established in the IRT system to support unblinding during the study.

In the event of urgent situation, the investigator may decide whether to trigger emergency unblinding to know which study treatment the subject received. Subject safety must always be the first consideration in making such a determination. If the investigator decides that unblinding is warranted, the investigator should make every effort to contact the Sponsor prior to unblinding unless this could delay further management of the subject. After emergency unblinding, the investigator must notify the Sponsor within 24 hours, and the reasons, time, and personnel performing the unblinding should be documented.

5.5.4. Routine Unblinding

The data lock plans to be performed after the last enrolled subject has completed the visit at 28 days after dosing. Data entry, data cleaning, blind data review and other necessary lock procedures should be completed before the lock. In principle, no subsequent modifications will be made to the locked data unless any significant data entry error that the investigator and the

Sponsor confirm and decide to correct. Before these data unlocking and modification, an application shall be submitted along with reasons and approved. Data that are not locked or newly generated can continue to be entered into the database.

Unblinding for primary analysis : the project statistician will submit an unblinding application after the database is locked. After approval from the Sponsor, the randomization statistician will send the blind code to the unblinded statistician independent of the project for analysis. This unblinding is scope-limited, only relevant Sponsor staff will be informed of the unblinded analysis result. The Sponsor will clearly document the personnel who are informed of the unblinded analysis result and the measures taken to maintain the double-blind status for the rest of the study. The entire study is still being performed in a blinded manner.

Unblinding at end of study: at the end of the entire clinical study, the data will be completely locked after data entry and data cleaning are completed and the blind verification is completed. The entire study will be unblinded after the database is completely locked. After the unblinding application is submitted by the project statistician and approved by the Sponsor, the randomization statistician will send the randomization schedule to the designated recipient (e.g., project statistician, Sponsor representative, etc.).

5.6. Study Drug Compliance

All investigational and non-investigational products will be administered by designated study staff at the investigator site. The administration of TNM002, HTIG, and tetanus vaccines will be recorded in the eCRF for the assessment of compliance with the investigational and non-investigational products.

5.7. Concomitant Medications and Therapies

Any concomitant medications or therapies received by subjects during the study (from the signing of the ICF) until the end of the study should be recorded. The drug name (or therapy name), dosage, and time should be recorded in the case report form.

5.7.1. Prohibited Medicines

The following medications are prohibited during the study except for the protocol-specified study drugs and those permitted during the study as specified in Section 5.7.2:

- Immunoglobulins (passive immunizing agents for tetanus, etc.), blood or blood products.
- Live virus vaccine (prohibited within 3 months after dosing).

5.7.2. Permitted During the Study

- Hormonal contraceptives that meet the requirements of this study are allowed to be used in subjects who are women of childbearing potential (WOCBP).
- The investigator is allowed to intervene by giving the subject the necessary medication or treatment based on the subject's adverse event.
- Medicinal products or treatments required for injury itself or comorbidities are permitted.

- Concomitant medications or therapies necessary for the treatment of prior comorbid chronic diseases are permitted.
- The investigator is allowed to use necessary medication or treatment (including passive immunizing agents for tetanus) due to tetanus occurrence.

5.7.3. Concomitant administration of Vaccine

While receiving the study drug, subjects with concomitant vaccine administration will receive adsorbed tetanus vaccine for adults and it will be intramuscularly injected into deltoid muscle of upper arm. The vaccine will be provided by study sites qualified to administer tetanus vaccines. The preparation and vaccination will be conducted according to the manufacture instructions. See the Pharmacy Manual for details.

5.8. Early Withdrawal and Lost to Follow-up

5.8.1. Early Withdrawal from Study

A subject is considered to have completed the study if he/she has completed all periods of the study including the last visit or the last scheduled procedure shown in the SoA.

Participants may withdraw from the study at any time at his/her own request, or may be withdrawn at any time at the discretion of the investigator for safety, behavioral, or compliance reasons.

Whenever possible, an attempt should be made to complete the premature withdrawal visit. SoA will provide the assessments to be performed at the time of early withdrawal from the study.

The premature withdrawal visit applies only to subjects who are randomized and then are prematurely withdrawn from the study. Participants should be questioned regarding their reasons for withdrawal. The subject will be permanently discontinued from the study at that time.

If a subject withdraws from the study, he/she may request destruction of any remaining samples, but data already generated from the samples will continue to be available and may be used to protect the integrity of existing analyses. The investigator must document any such requests in the site study records.

If the subject withdraws from the study and also withdraws consent for disclosure of future information, no further evaluations should be performed, and no additional data should be collected. The Sponsor may retain and continue to use any data collected before such withdrawal of consent.

Lack of completion of all or part of the early withdrawal procedures will not be viewed as protocol deviations so long as the subject’s safety was preserved.

If a subject explicitly withdraws consent from the investigator for any further contact with him/her, the investigator should be notified in writing whenever possible. The investigator should record the withdrawal of informed consent in detail in the medical record and enter relevant information into the eCRF.

5.8.2. Lost to Follow-up

A subject will be considered lost to follow-up if he or she repeatedly fails to return for scheduled visits and is unable to be contacted by the study site.

The following actions must be taken if a subject fails to return to the clinic for a required visit:

- The site must attempt to contact the subject and reschedule the missed visit as soon as possible and counsel the subject on the importance of maintaining the assigned visit schedule and ascertain whether or not the subject wishes to and/or should continue in the study.
- Before a subject is deemed lost to follow-up, the investigator or designee must make every effort to regain contact with the subject (3 telephone calls or appropriate local equivalent methods). These contact attempts should be documented in the subject's medical record.
- Should the subject continue to be unreachable, he/she will be considered withdrawn from the study.

6. STUDY PROCEDURES

Refer to SoA for all procedures to be performed throughout the study.

6.1. Screening (within 24 hours prior to dosing)

The investigator (or an appropriate delegate at the investigator site) must obtain a signed and dated ICF before performing any study-specific procedures. All screening evaluations must be completed and reviewed to confirm that potential subjects meet all eligibility criteria.

The investigator will maintain a screening log to record details of all subjects screened and to confirm eligibility or record reasons for screening failure.

Screen failures are defined as subjects who consent to participate in the study but are not subsequently randomly assigned to receive the study drug. A minimal set of screen failure information is required to ensure transparent reporting of screen failure subjects to meet the Consolidated Standards of Reporting Trials (CONSORT) requirements and to respond to queries from regulatory authorities. These information includes demography, eligibility criteria related to screen failure, and related details. Participants who fail screening may not be re-screened.

The procedures to be conducted during the Screening period are listed as followed:

- Written informed consent
- Collection of past medical history, present medical history (current injury history), treatment history, and tetanus vaccination history
- Demographics
- Weight and height
- Vital signs
- Physical examination

- 12-lead ECG
- Serum pregnancy test, only for WOCBP
- Laboratory tests (including hematology, clinical chemistry, coagulation, and urinalysis)
- Review of inclusion/exclusion criteria

6.2. Visit 1 (dosing)

The following procedures should be performed as soon as possible after the subject is confirmed to be eligible to participate in the study:

- Randomization
- Serum samples for the following tests will be collected within 1 hour prior to dosing
  - Anti-tetanus neutralizing antibody;
  - PK;
  - Immunogenicity;
- Vital Signs (If the screening vital sign measurement is performed within 2 hours prior to dosing, this is not necessary to be repeated)
- Study drug administration

  Note: procedures prior to study drug administration, including screening procedures, should be completed as soon as possible to ensure that dosing is completed within 24 hours after the injury.
- Participants should be observed in study site not less than 30 mins after study drug dosing
- An injection site reaction assessment should be performed 30 minutes after dosing of the study drug
- Adsorbed tetanus vaccine administration (only for subjects coadministered with vaccine)
- Collection of tetanus occurrence
- Collection of concomitant medications and current injury management
- Collection of AEs

6.3. Visit 2 (12 hours after dosing)

The Subject should return to the study site at 12 hours after dosing to collect serum samples for the following tests:

- Anti-tetanus neutralizing antibody;
- PK;

At this visit, the following procedures will also be completed :

- Vital signs
- Assessment of injection site
- Collection of tetanus occurrence
- Collection of concomitant medications
- Collection of AEs

6.4. Visit 3 (3 days after dosing)

The subjects should return to the study site to complete the following procedures at 3 days after dosing:

- Vital signs
- Assessment of injection site
- Serum samples will be collected for the following tests:
  - Anti-tetanus neutralizing antibody;
  - PK;
- Collection of tetanus occurrence
- Collection of concomitant medications
- Collection of AEs

6.5. Visit 4 (7 days after dosing)

The subjects should return to the study site to complete the following procedures at 7 days after dosing:

- Vital signs
- Physical examination
- 12-lead ECG
- Laboratory tests (including hematology, clinical chemistry, coagulation, and urinalysis)
- Serum samples will be collected for the following tests:
  - Anti-tetanus neutralizing antibody;
  - PK;
  - Immunogenicity;
- Collection of tetanus occurrence
- Collection of concomitant medications
- Collection of AEs

6.6. Visit 5 (28 days after dosing) & Visit 6 (90 days after dosing)

The subjects should return to the study site to complete the following procedures at 28 and 90 days after dosing, respectively:

- Vital signs
- Physical examination
- 12-lead ECG
- Laboratory tests (including hematology, clinical chemistry, coagulation, and urinalysis)
- Serum pregnancy test
- Serum samples will be collected for the following tests:
  - Anti-tetanus neutralizing antibody;
  - PK;
  - Immunogenicity;
- Collection of tetanus occurrence
- Collection of concomitant medications
- Collection of AEs

6.7. Visit 7 (105 days after dosing)

The subjects should be contacted by telephone to complete the following procedures at 105 days after dosing:

- Collection of tetanus occurrence
- Collection of concomitant medications
- Collection of AEs

6.8. End of Study Definition

The end of study includes the completion of the study and the early discontinuation of the study. The completion of the study is defined as the date of the last visit of the last subject in the study or last scheduled procedure shown in the SoA for the last subject.

The early discontinuation of the study refers to the discontinuation of the entire study or part of the study (such as certain site) while the study has not been completed for all subjects as per the protocol. In general, the study will not be discontinued early at will. However, the entire study (or certain site) may be discontinued early if any of the following occurs:

- The total sample size for competitive enrollment has met the study requirements, but the planned enrollment number at the site has not been completed as contracted and/or all subjects at the site have ended the study;
- The investigator at the site is unable to comply with the protocol or Good Clinical Practice (GCP);

- New information is obtained that shows an unfavorable risk-benefit evaluation of investigational drug, including sufficient evidence suggesting efficacy deficiency or unacceptable safety;
- The Sponsor considers it inappropriate to continue the clinical study for medical, ethical, or commercial reasons;
- Discontinuation is ordered by the National Medical Products Administration or the Ethics Committee for certain reason;
- If the clinical study is discontinued early, all parties (Sponsor, investigator, EC, clinical study institution and administrative authority) should be notified in writing in a timely manner.

7. STUDY ASSESSMENTS

Study procedures and their timing are summarized in the SoA. Adherence to the study design requirements, including those specified in the SoA, is essential and required for study conduct.

7.1. Efficacy Assessments

7.1.1. Assessment of Anti-Tetanus Neutralizing Antibody Titer

The time points for collection of serum samples for anti-tetanus neutralizing antibodies are shown in the SoA.

The time window for these samples is the same as the time window of follow-up visits.

Test samples for anti-tetanus neutralizing antibodies must be collected, processed, stored and shipped as indicated in the Lab Manual provided to the investigator site. Any deviations during sample collection, handling, storage and shipment, including any actions taken, must be documented and reported to the Sponsor. On a case-by-case basis, the Sponsor may make a determination as to whether sample integrity has been compromised.

Samples collected for measurement of serum concentrations of investigational drug will be analyzed using a validated analytical method in compliance with applicable standard operating procedures (SOPs).

7.1.2. Assessment of Tetanus Protection Rate

The time points for collection of tetanus occurrence will last from enrollment to the end of the study. The diagnostic basis for tetanus is detailed in Appendix 2. As the diagnosis of tetanus is mainly based on clinical symptoms and no reliable objective diagnostic criteria are available, a clinical adjudication committee consisting of clinical experts in the field of tetanus disease will be established. For subjects with tetanus-related symptoms, the final diagnosis will be made by experts in this committee after discussion.

7.2. Safety Assessment

Planned timepoints for all safety assessments are provided in the SoA.

Unscheduled clinical laboratory measurements may be obtained at any time during the study to assess any perceived safety concerns. Then test results should be followed up at the discretion

of the investigator until it has returned to the normal range or stabilized, and/or a diagnosis is made to adequately explain the abnormality.

7.2.1. Physical examination

Timepoints for physical examinations are provided in the SoA.

The physical examination generally includes skin, lymph nodes, eyes, head and neck, chest, abdomen, spine, extremities, etc. The frequency of physical examinations may be increased if necessary.

Investigators should pay special attention to clinical signs related to previous serious illnesses.

7.2.2. Vital signs

Timepoints for vital signs are provided in the SoA.

The vital signs mainly include blood pressure, pulse rate, respiratory rate, and body temperature (ear temperature).

Systolic and diastolic blood pressures will be measured each time after the subject has rested quietly for at least 5 minutes. All measurements and recording will be performed using calibrated equipment. Both automated and manual measurements will be acceptable, but all assessments for individual subjects should be performed using the same measurement method during the study. When scheduled at the same timepoints, vital signs and 12-lead ECG must be assessed first before physical examinations, blood sample collections, and urine collection.

7.2.3. 12-lead ECG

Timepoints for 12-lead ECG are provided in the SoA.

All scheduled ECGs should be performed after the subject has rested quietly for at least 10 minutes. Results should be obtained using an electrocardiograph that automatically calculates the heart rate and measures PR, QT, and QTc intervals and QRS complex.

In some cases, it may be appropriate to repeat abnormal ECGs to rule out improper lead placement as contributing to the ECG abnormality. It is important that leads are placed in the same positions each time in order to achieve precise ECG recordings.

7.2.4. Safety Laboratory Assessments

The laboratory tests include hematology, clinical chemistry, coagulation and urinalysis. Timepoints are provided in the SoA.

The investigator is required to review and document the laboratory results, and record any clinically relevant changes occurring during the study on the AE page of the eCRF.

All laboratory tests with values considered clinically significantly abnormal during the study should be repeated regularly until the values return to normal or baseline or are no longer considered clinically significant by the investigator. If these values do not return to normal or baseline afterquiet a reasonable time period , the etiology should be identified, and the sponsor should be notified when available.

If laboratory values from non-protocol-specified laboratory assessments performed at the local laboratory are considered clinically significant by the investigator, then the results must be recorded in the eCRF.

The parameters of laboratory tests are described in Table 1.

Table 1: Laboratory Tests

<table>
<tr><th>Chemistry</th><th>Hematology</th><th>Urinalysis</th></tr>
<tr><td rowspan="3">• Total protein
• Albumin
• Sodium
• Potassium
• Chloride
• Calcium
• Glucose
• Aspartate aminotransferase
• Alanine aminotransferase
• Total bilirubin
• Direct bilirubin
• Urea (blood urea nitrogen)
• Creatinine</td><td>• Red blood cell count
• Hemoglobin
• Platelet count
• White blood cell count
• Absolute neutrophil count
• Neutrophil percentage
• Absolute lymphocyte count
• Lymphocytes percentage
• Absolute monocyte count
• Hematocrit</td><td>• Protein
• Glucose
• Urobilinogen
• Urine occult blood
• Red blood cell
• White blood cell</td></tr>
<tr><td>• Coagulation</td><td>Others</td></tr>
<tr><td>• Prothrombin time
• Activated partial thromboplastin time</td><td>• Serum pregnancy test (β-hCG)</td></tr>
</table>

7.2.5. Serum Pregnancy Test

Women of childbearing potential will undergo the serum pregnancy test to determine the pregnancy status. Timepoints are provided in the SoA.

Following a negative pregnancy test result at screening, effective contraception must be commenced.

7.2.6. Assessment of Injection Site

An injection site reaction assessment should be performed 30 (± 5) minutes, 12 hours (± 30 mins) and 3 days (± 4 hours) after IM injection of the study drug, and the local reactions at the injection site will be closely monitored and recorded.

7.3. Pharmacokinetics

Timepoints for collection of PK serum samples are provided in the SoA. The time window for these samples is the same as the time window of follow-up visits.

Drug concentration-time relationship will be summarized descriptively. In order to describe the PK properties and to identify and quantify the source and extent of PK variation, the nonlinear mixed effect model (NONMEM) will be used to establish a PopPK model of TNM002. PK/PD analysis will also be carried out, if appropriate.

The PK samples must be collected, processed, stored and shipped as indicated in the Lab Manual provided to the investigator site. Any deviations during PK sample collection, handling, storage and shipment, including any actions taken, must be documented and reported to the Sponsor. On a case-by-case basis, the Sponsor may make a determination as to whether sample integrity has been compromised.

Samples collected for measurement of serum concentrations of investigational drug will be sent to a central bioassay laboratory and analyzed using a validated analytical method in compliance with applicable SOPs.

7.4. Pharmacodynamics

The PD endpoint in this study is the level of anti-tetanus neutralizing antibody, and no other PD endpoints are involved.

7.5. Immunogenicity Evaluation

Timepoints for collection of immunogenicity serum samples are provided in the SoA.

The time window for these samples is the same as the time window of follow-up visits.

Immunogenicity will be evaluated by the rate of ADA positivity in the subject and, if confirmed as ADA positive, by further testing for Neutralizing antibodies (NAbs). The effect of ADA on the PK profile and safety of the study drug will be further evaluated if the data allow. The immunogenicity samples must be collected, processed, stored and shipped as indicated in the Lab Manual. Any deviations during immunogenicity sample collection, handling, storage and shipment, including any actions taken, must be documented and reported to the Sponsor. On a case-by-case basis, the Sponsor may make a determination as to whether sample integrity has been compromised. Samples collected for immunogenicity analysis will be analyzed using a validated analytical method in compliance with applicable SOPs.

7.6. Other Evaluations

7.6.1. Demographic Information

The demographic data, including age, date of birth, gender, height, weight, ethnicity, etc., will be collected at screening and recorded in the eCRF.

7.6.2. Medical, Treatment and Vaccination History

Medical history refers to the information on prior or concomitant diseases that exist prior to dosing, including history of infectious diseases, surgery, injury and allergy. The medical history will be recorded in the original record and eCRF.

Treatment history refers to the information on prior or concomitant treatments that exist prior to dosing, including history of tetanus vaccination, immunoglobulins or blood products, mAb, any drugs (including prescription drugs, over-the-counter medications and Chinese herbal medicines) and health care products, which should be recorded in the eCRF during the screening period. Participants with specific prior diseases cannot participate in this clinical study (see Inclusion Criteria in Section 4.1 and Exclusion Criteria in Section 4.2).

8. ADVERSE AND SERIOUS ADVERSE EVENTS

The investigator and any qualified designees are responsible for collecting and recording events that meet the definition of an AE or SAE and remain responsible to pursue and obtain adequate information until the outcome is stable or the patient is lost to follow up.

In addition, the investigator may be requested by Trinomab Pharmacovigilance to obtain specific follow-up information in an expedited fashion.

8.1. Definition of AE

An AE is defined as any untoward medical event that occurs after a subject receives the study drug, which may be manifested as diseases, symptoms, signs, or laboratory abnormalities, but may not necessarily have a causal relationship with the study drug.

Examples of AEs include but not limited to:

- Any abnormal laboratory test results (hematology, clinical chemistry, or urinalysis) or other safety assessments (eg, ECG, imaging, vital signs), including those that worsen from baseline, considered clinically significant upon medical and scientific judgment of the investigator (ie, not related to progression of underlying disease).

The criteria for determining whether an abnormal objective test finding should be reported as an AE are as follows:

  - Test result is accompanied with symptoms; and/or
  - Test result requires extra diagnostic testing or medical/surgical intervention; and/or
  - Test results leads to the addition of concomitant medications or other treatments, and/or
  - Test result is considered to be an AE by the investigator or Sponsor.

- New conditions detected or diagnosed after study drug administration even though it may have been present before the start of the study.
- Symptoms, signs, or the clinical sequelae of a suspected drug-drug interaction.

The following events are not considered to meet the AE definition.

- Medical or surgical procedure (eg, endoscopy, appendectomy): the medical condition that leads to the procedure is AE.
- Anticipated day-to-day fluctuations of preexisting disease(s) detected at the start of the study that do not worsen.
- “Lack of efficacy” or “failure of expected pharmacological action” per se will not be reported as an AE or SAE. Such instances will be captured in the efficacy assessments. However, the signs, symptoms, and/or clinical sequelae resulting from lack of efficacy will be reported as an AE or SAE if they fulfill the definition of an AE or SAE.
- Any clinically significant abnormal laboratory findings or other abnormal safety assessments which are associated with the underlying disease, unless judged by the investigator to be more severe than expected for the subject’s condition.

- The expected progression, signs, or symptoms of the disease being studied, unless more severe than expected.
- In this study, tetanus incidence will be collected as a secondary efficacy endpoint rather as an AE.

8.2. Time Period of Collecting AE

For each subject, the period for proactive solicitation and collection of adverse events (AE) (proactive collection period) extends from the subject's first dose of study drug until the Day 105 visit. For subjects who are withdrawn prematurely, the AE collection period will continue until the last visit or 7 days after the early withdrawal, whichever comes late.

The investigator should continue to follow up AEs until the subject has recovered or recovered with sequelae, returned to baseline, or stabilized to an acceptable level as judge by the investigator. The follow-up for AEs may last beyond the active collection period.

Investigators are not obligated to actively seek AEs or SAEs after end of the study. However, if the investigator learns of any SAE, including a death, at any time after a subject has been discharged from the study, and he/she considers the event to be reasonably related to the study drug or study participation, the investigator must promptly notify the Sponsor.

8.3. Recording of AEs

During the active collection period, all AEs will be recorded on the CRF including information as following:

- AE,
- Start time,
- Seriousness,
- Severity,
- Causal relationship to study drug,
- Action taken to study drug,
- Other action taken,
- Outcome,
- End time.

8.3.1. AE

AEs should be recorded as diagnoses of certain diseases, if available. Otherwise, the respective symptoms and signs should be recorded. If a subject suffers from the same AE more than once and the subject recovers in between the events, the AEs should be recorded separately.

During the study, AEs related to injection site reactions will be collected in a solicited manner.

Note the following: A procedure is not an AE; the reason for conducting the procedure is. Hospitalization is not an AE; the reason for hospitalization is. Death is not an AE, but the cause of death is (an exception is sudden death of unknown cause, which is an AE).

8.3.2. Start Date and Time

The start date and time corresponding to the date and time when the first symptom(s) or sign(s) were noted. If the AE is an abnormal clinically significant laboratory test or outcome of an examination, the onset date and time are those on which the sample was taken, or the examination was performed.

8.3.3. Assessment of Severity

The severity of an AE is defined as a qualitative assessment of the degree of intensity of an AE as determined by the investigator or reported to him/her by the subject. The severity does not reflect the clinical seriousness of the event, only the degree or extent of the affliction or occurrence, and does not reflect the relationship to the study drug. AEs will be classified as mild, moderate and severe in severity as follows:

- Mild; asymptomatic or mild symptoms; clinical or diagnostic observations only; or intervention not indicated;
- Moderate; requiring small, local or noninvasive treatment; restricted in age-appropriate instrumental activities of daily living (ADLs)(ADLs refer to cooking, shopping for clothing, using the telephone, managing money, etc.);
- Severe: serious or medically significant but not immediately life-threatening; requiring hospitalization or prolongation of hospitalization; disabling; restricted in self-care ADLs (ADLs refer to bathing, dressing, undressing, eating, toileting, taking medications, etc., rather than bedridden).

If an AE changes in intensity, a worst-case approach should be used when recording the event, i.e., the highest intensity and the longest duration of the event.

8.3.4. Assessment of Causality

The investigator is responsible for assessing the causal relationship between the study drug and the AE, i.e., determining whether there exists a reasonable possibility that the study drug caused or contributed to an AE.

The correlation between all AEs occurring during the course of the clinical study and the study drug needs to be assessed. The following factors should be considered in judging the relationship of AEs to the study drug:

- Whether there is a temporal relationship between the AE and the use of the study drug;
- Whether the AE disappears or alleviates after discontinuation of the study drug;
- Whether the AE is consistent with the known knowledge of the pharmacology and toxicology of the study drug. Whether there are clinical symptoms, laboratory tests, or imaging manifestations to support the confirmation of correlation;
- Whether the AE can be explained by the subjects past medical history, current medical history, psychological factors, concomitant medications or treatments, other environmental factors, etc.

Investigators should use their knowledge of the subject, the circumstances surrounding the event, and an evaluation of any potential alternative causes to determine whether an AE is considered to be related to the study drug.

The causal relationships to the study drug include "Related" and "Not Related" .

- Related: There is a plausible temporal relationship between the onset of the AE and the administration of study treatment, and the AE cannot be explained by the subjects past medical history, current medical history, or concomitant therapies; and/or the AE follows a known pattern of response to study treatment; and/or the AE abates or resolves upon discontinuation of study treatment or dose reduction;
- Not Related: Evidence exists that the AE has an etiology other than study treatment (e.g., past medical history, underlying disease, intercurrent illness, or concomitant medication); and/or the AE has no plausible temporal relationship to administration of study drug (e.g., cancer diagnosed 2 days after first dose of study drug).

8.3.5. Action Taken with the Study Drug

The action taken with regard to the study drug in response to an AE will be classified as one of the following:

- No change (medication schedule maintained or no action taken),
- Withdrawn,
- Interrupted,
- Dose reduced,
- Dose increased,
- Not applicable.

The study drugs are required to be administrated only once, so the "actions taken" should be "not applicable".

8.3.6. Other Action Taken

AEs requiring therapy must be treated with recognized standards of medical care to protect the health and well-being of the subject.

8.3.7. Outcome and Date and Time of Outcome

The outcome of an AE will be classified as one of the following:

- Recovered (without sequelae): the event has returned to normal or baseline/is considered stable by the investigator. The "AE end date" will be recorded, and the follow-up can be ended;
- Recovered (with sequelae): the AE has recovered with chronic or lifelong sequelae (e.g., blindness caused by diabetes mellitus, hemiplegia caused by stroke). The "AE end date" will be recorded, and the follow-up can be ended;

- Recovering/resolving: the AE is recovering/resolving, and further follow-up is required;
- Not recovered/not resolved/ongoing: the severity of the event is not upgraded, the event is not resolved and is ongoing, and further follow-up is required;
- Fatal: when the AE is the cause of death of the subject, the time of death should be recorded;
- Unknown: the investigator cannot obtain the outcome of an AE, for example, when the subject is lost to follow-up;

If the outcome of an AE is "not recovered/not resolved/ongoing" or "unknown", the AE end date may not be recorded temporarily. If the outcome of an AE is "recovered/resolved (without sequelae)", "recovered (with sequelae)" or "fatal", the AE end date must be recorded.

8.4. Adverse Events of Special Interest

Adverse events of special interest (AESI) are events of scientific and medical interest to the study drug and may require close monitoring and prompt communication with the sponsor by the investigator. AESI can be serious or non-serious. AESI expedited reporting enables continuous monitoring of these events in order to describe and understand their association with use of the study drug.

The AESI of this study is hypersensitivity reactions. The investigator should submit a SAE Report Form to the Sponsor within 24 hours after becoming aware of hypersensitivity reactions and record the event on the AE page of the eCRF.

Although no hypersensitivity reactions (including anaphylactic reaction and events with fatal outcome) have been observed in subjects treated with TNM002 in previous studies, sites should still have drugs and emergency equipment to manage these reactions. Participants will be closely observed. Once allergic reaction symptoms such as hypotension, dyspnea, throat tightness, facial or upper respiratory tract edema, pruritus, urticaria occur after the injection, treatment should be provided immediately (see Section 8.5.1).

8.5. Special Precautions and Management of AEs

8.5.1. Anaphylaxis

All therapeutic monoclonal (mAbs) have the potential to cause hypersensitivity, or even anaphylaxis. As a routine precaution, all subjects will receive the study treatment as inpatients so that close monitoring for signs or symptoms of anaphylaxis, and proper treatments can be provided.

In case anaphylaxis occurs, the following interventions may be considered:

- Start IV saline infusion; recommend bronchodilators, epinephrine 0.01 mg/kg per single dose intramuscular injection, and/or diphenhydramine 50 mg IV with methylprednisolone 100 mg IV (or equivalent), as needed.
- Participants should be monitored until the investigator is satisfied that the symptoms will not recur. Investigators should follow their institutional guidelines for the

treatment of anaphylaxis. In the case of late-occurring hypersensitivity symptoms (for example, appearance of a localized or generalized pruritis within 1 week after treatment), symptomatic treatment may be given (e.g., oral antihistamine, or corticosteroids).

8.6. DEFINITION OF SAE

An SAE is defined as an untoward medical event that meets any of the following criteria after a subject receives the study drug at any dose:

1) **Resulting in death**

2) **Life-threatening**

"Life-threatening" refers to an immediate risk of death faced by a subject at the time of the AE, rather than a speculated death that may occur in the future if the event gets worse.

3) **Results in hospitalization or prolongation of an exist hospitalization**

In general, hospitalization means that the subject has been detained (usually involving at least an overnight stay) at the hospital or emergency ward for observation and/or treatment that would not have been appropriate in the physician's office or outpatient setting. Complications that occur during hospitalization are AEs. If a complication prolongs hospitalization or fulfils any other serious criteria, the event is serious. When in doubt as to whether "hospitalization" occurred or was necessary, the AE is to be considered serious.

Hospitalization for elective treatment of a preexisting condition that did not worsen from baseline is not considered an AE.

4) **Resulting in permanent or significant disability/dysfunction**

The term disability means a substantial disruption of a person's ability to conduct normal life functions.

This definition is not intended to include experiences of relatively minor medical significance such as uncomplicated headache, nausea, vomiting, diarrhea, influenza, and accidental trauma (eg, sprained ankle) which may interfere with or prevent everyday life functions but do not constitute a substantial disruption.

5) **Congenital anomalies/birth defects**

6) **Important medical event**

Medical or scientific judgment should be exercised in deciding whether SAE reporting is appropriate in other situations, such as important medical events that may not be immediately life-threatening or result in death or hospitalization but may jeopardize the subject or may require medical or surgical intervention to prevent one of the other outcomes listed in the above definition. These events should usually be considered serious.

Examples of such events include invasive or malignant cancers, intensive treatment in an emergency room or at home for allergic bronchospasm, blood dyscrasias or convulsions that do not result in hospitalization, or development of drug dependency or drug abuse.

In this study, tetanus incidence will be collected as a secondary efficacy endpoint rather as an AE. Hospitalizations or life-threatening events due to tetanus will also no longer collected as SAEs.

8.7. Collecting and Reporting of SAE

All SAEs occurring in a subject during the active collection period are reported to Trinomab Pharmacovigilance Department on the SAE Report Form immediately and under no circumstance should this exceed 24 hours after the investigator becomes aware of them.

There may be situations in which an SAE has occurred, and the investigator has minimal information to include in the initial report to the Sponsor. However, **it is very important that the investigator always make an assessment of causality for every event before the initial transmission of the SAE data to the Sponsor.** The investigator may change his/her opinion of causality in light of follow-up information and send an SAE follow-up report with the updated causality assessment.

After the initial SAE report, the investigator is required to proactively follow each subject at subsequent visits/contacts. For each event, the investigator must pursue and obtain adequate information until resolution, stabilization, the event is otherwise explained, or the subject is lost to follow-up.

In general, follow-up information will include a description of the event in sufficient detail (including any information relevant to the event, such as concomitant medications and illnesses) to allow for a complete medical assessment of the case and independent determination of possible causality. In the case of a subject death, a summary of available autopsy findings must be submitted as soon as possible to Trinomab Pharmacovigilance Department.

The investigator will submit any updated SAE data to the Sponsor within 24 hours of it being available.

SAEs occurring after the active collection period of AEs has ended are reported to Trinomab Pharmacovigilance Department if the investigator becomes aware of them and believes the SAE have at least a reasonable possibility of being related to study drug.

8.8. Regulatory Reporting Requirements for SAEs

Prompt notification by the investigator to the Sponsor of an SAE is essential so that legal obligations and ethical responsibilities towards the safety of subjects and the safety of a study drug under clinical investigation are met.

The Sponsor has a legal responsibility to notify both the local regulatory authority and other regulatory agencies about the safety of a study drug under clinical investigation. The Sponsor will comply with country-specific regulatory requirements relating to safety reporting to the regulatory authority, ECs, and investigators.

Investigator safety reports must be prepared for suspected unexpected serious adverse reactions (SUSARs) according to local regulatory requirements and Sponsor policy and forwarded to investigators as necessary.

An investigator who receives an investigator safety report describing an SAE or other specific safety information (eg, summary or listing of SAEs) from the Sponsor will review and then file it along with IB and will notify the EC, if appropriate according to local requirements.

8.9. Exposure During Pregnancy

If a female subject or female partner of a male subject is directly or indirectly exposed to the study drug during pregnancy, the investigator should report it to Trinomab Pharmacovigilance within 24 hours of awareness and follow up until the outcome of the pregnancy (delivery or termination of pregnancy including spontaneous and induced abortions). The collection interval for pregnancy events is the same as the collection interval for AEs as detailed in Section 8.2.

Abnormal pregnancy outcomes (eg, spontaneous abortion, fetal death, stillbirth, congenital anomalies, ectopic pregnancy) are considered SAEs.

9. STATISTICAL ANALYSIS

The general principal for statistical analyses is outlined here and further detailed in a statistical analysis plan (SAP). The SAP may modify what is outlined in the protocol where appropriate; however, any major modifications of the primary endpoint or its analysis must be reflected in a protocol amendment.

9.1. Sample Size Determination

Approximately 675 subjects will be randomized such that approximately 540 evaluable subjects will complete the study.

Primary Hypothesis:

The hypothesis is that TNM002 has efficacy that is superior to HTIG as prophylaxis against tetanus. To be more specific, the lower limit of the 95% confidence interval (CI) of the difference (TNM002 group - HTIG group) in the proportion of subjects with Δtiters ≥ 0.01 IU/mL at 12 hours after the receipt of study drug is > 0.

The sample size estimation is based on the above hypothesis and following assumptions: (1) The proportion of subjects with Δ titers ≥ 0.01 IU/mL at 12 hours after receipt of TNM002 and HTIG based on results of earlier clinical trials; (2) Due to the fact that HTIG is a well-studied product whose efficacy and safety does not need to be evaluated in large amount of subjects in a clinical trial setting, the randomization ratio between TNM002 and HTIG groups is 2:1; (3) One-sided Type I error of 0.025; (4) TNM002 requires adequate exposure during clinical development to fully understand its safety.

Taken together, the estimated sample size is approximately 540 subjects, i.e., 360 in the TNM002 group and 180 in the HTIG group. This will provide a power of >99% to evaluate the above efficacy hypothesis. The dropout rate is expected to be 20% in this study. Therefore, the planned enrollment will be 675 subjects, i.e., 450 in the TNM002 group and 225 in the HTIG group.

Secondary Hypothesis:

The tetanus protection rate is assumed to be 99.99% within 28 days post-dose in the HTIG group and 99.999% within 28 days post-dose in the TNM002 group. The current sample size will not provide sufficient power to test a formal statistical hypothesis in terms of tetanus protection rate between TNM002 and HTIG.

Safety Hypothesis:

Table 2 shows the probability of observing at least 1 AE for a given true event rate of a particular AE, for various sample sizes. For example, if the true AE rate is 1%, there is a 95.1% probability of observing at least 1 AE in 300 subjects.

Table 2: Probability of Observing at Least One Adverse Event at Different Sample Sizes and Different True Incidences of Adverse Events

Assumed True Incidence of AEs	Probability of observing at least one AE at different sample sizes			
	N=200	N=300	N=500	N=600
0.10%	18.0%	25.9%	39.4%	45.2%
0.50%	63.3%	77.8%	91.8%	95.1%
1.00%	86.6%	95.1%	>99.0%	>99.0%
2.00%	98.2%	>99.0%	>99.0%	>99.0%
3.00%	>99.0%	>99.0%	>99.0%	>99.0%
4.00%	>99.0%	>99.0%	>99.0%	>99.0%
5.00%	>99.0%	>99.0%	>99.0%	>99.0%
6.00%	>99.0%	>99.0%	>99.0%	>99.0%
7.00%	>99.0%	>99.0%	>99.0%	>99.0%
8.00%	>99.0%	>99.0%	>99.0%	>99.0%
9.00%	>99.0%	>99.0%	>99.0%	>99.0%
10.00%	>99.0%	>99.0%	>99.0%	>99.0%

9.2. Analysis Set

For the purposes of analysis, the following analysis sets are defined:

**Analysis Set**	**Description**
All-Screened Set	All subjects who have signed the informed consent form.
Intention-to-Treat (ITT) Population	All subjects who have been randomized.
Full Analysis Set (FAS)	All randomized subjects who have received the study drug.
Safety Set (SS)	All randomized subjects who have received the study drug with at least one post-dose safety evaluation.

Immunogenicity Set (IAS)	All randomized subjects who have received TNM002 with at least one post-dose immunogenicity data point.
Pharmacokinetic Set (PKS)	All randomized subjects who have received TNM002 with at least one post-dose evaluable PK concentration. If the PK concentration results are affected by important protocol deviations, all or partial PK data of corresponding subjects will not be included in the PK analysis.
Per Protocol Set (PPS)	All subjects in the FAS who do not have any important protocol deviations considered to have a major effect on the primary efficacy endpoint.

9.3. Statistical Analysis

PK parameters will be calculated using Phoenix WinNonlin version 7.0 or above, and all remaining statistical analyses will be performed using SAS version 9.4 or above. For descriptive statistics, continuous variables will be described using the number of subjects, mean, median, standard deviation (SD), minimum (Min) and maximum (Max); while categorical variables will be described using frequencies and percentages for each category. In addition, applicable PK concentrations and PK parameters will be presented using coefficient of variance (CV), geometric mean (GM), and geometric CV.
Data conversion, missing values, out-of-window sampling, handling of outliers and sensitivity analysis will be described in detail in the SAP.

9.3.1. Efficacy Analysis

9.3.1.1. Primary Efficacy Analysis

In all randomized subjects who have received the study drug, the proportion of subjects with Δ titers ≥ 0.01 IU/mL from baseline at 12 hours after receipt of study drug and its two-sided 95% CI (by Clopper-Pearson method) will be calculated for each treatment group. The difference in the proportions between the TNM002 and HTIG groups and its two-sided 95% CI (stratified by with or without concomitant administration of tetanus vaccine as determined before randomization) will also be calculated.

Subjects who are diagnosed with tetanus within 12 hours after receipt of study drug, or have received additional passive immunizing agent due to the original wound, or have experienced AE leading to study discontinuation within 12 hours after receipt of study drug, are considered to have a Δ titer < 0.01 IU/mL.

Subgroup analysis, handling of additional intercurrent events, and other details are provided in statistical analysis plan (SAP).

9.3.1.2. Secondary Efficacy Analysis

In all randomized subjects who have received study drug, the tetanus protection rate within 28 days after receipt of study drug and its two-sided 95% CI (by Clopper-Pearson method) will be calculated for each treatment group. The difference in tetanus protection rate between the TNM002 and HTIG groups and its two-sided 95% CI (stratified by with or without concomitant administration of tetanus vaccine as determined before randomization) will also be calculated.

Subgroup analysis, handling of intercurrent events, and other details are provided in SAP.

9.3.1.3. Other Efficacy Analyses

9.3.1.3.1. Anti-tetanus neutralizing antibody titers at 3, 7, 28 and 90 days after receipt of study drug

This anti-tetanus neutralizing antibody titer analysis will be performed in all randomized subjects who have received study drug, and haven't received tetanus vaccine up to the respective time points. Subjects who are diagnosed with tetanus as of the respective time point, or have received additional passive immunizing agent due to the original wound, are considered to have an antibody titer of LLOQ/2 and a Δ titer < 0.01 IU/mL at that time point.

9.3.1.3.1.1. Proportion of subjects with Δ titers ≥ 0.01 IU/mL at 3, 7, 28 and 90 days after receipt of study drug, respectively

Using methods described in Section 9.3.1.1, the proportion of subjects with Δ titers ≥ 0.01 IU/mL from baseline at 3, 7, 28 and 90 days after receipt of study drug and its two-sided 95% CI will be calculated for each treatment group. The difference in the proportions between the TNM002 and HTIG groups and its two-sided 95% CI will also be calculated.

9.3.1.3.1.2. Geometric mean of Δ titers at 3, 7, 28 and 90 days after receipt of study drug, respectively

Δ Titers will be summarized by treatment group at each scheduled time point using summary statistics including median, Min, Max, GMT, geometric SD, and 95% CI of GMT.

In addition, Δ titers at each scheduled time point after dosing will be compared between treatment groups based on the mixed-effect model for repeated measures (MMRM). The MMRM model will use the log-transformed Δ titers at each scheduled time point as the dependent variable, the treatment group, time point, and treatment group-time point interaction as the independent variables, and the baseline anti-tetanus neutralizing antibody titer as the covariate. The intra-subject variance-covariance structure will be the unstructure (UN). The difference in Δ titers and 95% CI of the difference between different treatment groups will be calculated and then antilog-transformed to obtain the ratio of GMT and CI of ratio. If the model does not converge when the covariance structure is UN, other possible covariance structures, such as Toelitz, autoregression (1) (AR (1)), etc., can be selected, and the covariance structure with the smallest final AIC value will be used for the final model.

9.3.1.3.1.3. Anti-tetanus neutralizing antibody titers in subjects who receive concomitant administration of tetanus vaccine

In all randomized subjects who have received study drug, and tetanus vaccine as determined before randomization, the anti-tetanus neutralizing antibody titers and its increase from baseline will be descriptively summarized.

9.3.1.3.2. Tetanus protection rate within 90 and 105 days after receipt of study drug

Using methods described in Section 9.3.1.2, tetanus protection rate within 90 and 105 days after receipt of study drug will be compared in randomized subjects who receive study drug.

Details for analysis methods including subgroup analysis are provided in the SAP.

9.3.2. Safety Analysis

Safety analysis will be performed in randomized subjects who have received study drug and having at least one post-dose safety evaluation. Participants will be analyzed according to the study drug they actually receive.

AEs will be coded using the Medical Dictionary for Regulatory Activities (MedDRA) version 25.0 or higher. The number and incidence of all AEs, AEs related to the study drug, and SAEs in both treatment groups will be calculated and presented by system organ class (SOC) and preferred term (PT) based on the safety set.

The quantitative safety variables such as laboratory tests, vital signs, ECGs, and their changes from baseline will be summarized using descriptive statistics. The changes from baseline in safety variables such as laboratory tests, ECGs, and physical examinations will also be described using the shift tables. The test items with abnormal values and clinical significance will be listed.

Details for analysis methods including subgroup analysis are provided in the SAP.

9.3.3. PK Analysis

In all randomized subjects who have received TNM002 with at least one post-dose evaluable PK concentration, drug concentrations will be summarized descriptively by nominal collection time points of serum samples. In order to describe the PK properties and to identify and quantify the source and extent of PK variation, the nonlinear mixed effect model (NONMEM) will be used to establish a PopPK model of TNM002.

If the PK concentration results are affected by important protocol deviations, all or partial PK data of corresponding subjects will not be included in the analysis.

Details for analysis methods including subgroup analysis are provided in the SAP.

9.3.4. Immunogenicity Analysis

ADA positive rate (and the incidence of neutralizing antibody, if applicable) at baseline and at different post-baseline timepoints will be summarized by treatment group in all randomized subjects who have received TNM002 and having post-dose immunogenicity data. For ADA positive subjects, the number and percentage of NAb positive subjects will also be further calculated if data permit. The impact of ADA and/or NAb production on the efficacy, PK profile, and safety of the study drug will also be assessed, as applicable.

9.4. Analysis Timeline

The primary analysis will be performed after the last enrolled subject has completed the Day 28 visit. This analysis includes the final analysis of the primary and secondary efficacy endpoints and the interim analysis of the safety and other endpoints. Relevant Sponsor staff will be informed of the unblinded analysis result. The Sponsor will clearly document the personnel who are informed of the unblinded analysis result, and the measures taken to maintain the double-blind status for the remainder of the study. The investigators and study site staff will remain blinded throughout the study.

9.5. Data Monitoring Committee

Not applicable

10. REGULATORY, ETHICAL, AND STUDY OVERSIGHT CONSIDERATIONS

10.1. Regulatory and Ethical Considerations

This study will be conducted in accordance with the protocol and the following principles:

- Consensus ethical principles derived from international guidelines including the Declaration of Helsinki and Council for International Organizations of Medical Sciences (CIOMS) International Ethical Guidelines,

- International Council for Harmonisation (ICH) GCP guidelines,
- Applicable laws and regulations, including applicable privacy laws.

The protocol, protocol amendment, ICF, IB, and other relevant documents (eg, advertisements) must be approved by the Sponsor, submitted to an EC by the investigator and approved by the EC before the study is initiated.

The investigator will be responsible for the following:

- Providing written summaries of the status of the study to the EC annually or more frequently in accordance with the requirements, policies, and procedures established by the EC,
- Notifying the EC of any SAEs or other significant safety findings as required by EC procedures;
- Providing oversight of the conduct of the study at the site and adherence to, ICH guidelines, the EC, and all other applicable local regulations.

10.2. Informed Consent Process

The investigator or his/her representative will explain the nature of the study to the subject and answer all questions regarding the study. Participants must be informed that their participation is voluntary. Participants will be required to sign a statement of informed consent that meets the local regulations, ICH guidelines, and the EC or study site.

The investigator must ensure that each subject is fully informed about the nature and objectives of the study, the sharing of study data, and possible risks associated with participation, including the risks associated with the processing of the subject's personal data. The subject must be informed that his/her personal study data will be used by the Sponsor in accordance with local data protection law. The level of disclosure must also be explained to the subject.

The subject must be informed that his/her medical records may be examined by Clinical Quality Assurance auditors or other authorized personnel appointed by the Sponsor, by appropriate EC members, and by inspectors from regulatory authorities. The investigator further must ensure that each subject is fully informed about his or her right to access and correct his or her personal data and to withdraw consent for the processing of his or her personal data.

The medical record must indicate that documented informed consent was obtained before the subject entered the study and the date the written consent was obtained. The authorized person obtaining the informed consent must also sign the ICF.

If the ICF is updated during the study, the subject must be reconsented to the most current version of the ICF(s). An original ICF(s) must be provided to the subjects.

10.3. Data Protection

All parties in the study should comply with all applicable laws, including laws regarding the implementation of organizational and technical measures to ensure protection of subject data.

Participants' personal data will be stored at the study site in electronic form and will be password protected to ensure that only authorized study staff have access. The study site will implement appropriate technical and organizational measures to ensure that the personal data can be recovered in the event of disaster. In the event of a potential personal data breach, the study site shall be responsible for determining whether a personal data breach has in fact occurred and, if so, providing breach notifications to relevant parties as required by law.

To protect the rights and freedoms of natural persons with regard to the processing of personal data, subjects will be assigned a single, subject-specific numerical code. Any subject records or data sets that are transferred to the Sponsor will contain the numerical code; subject names will not be transferred. All other identifiable data transferred to the Sponsor will be identified by this single, subject-specific code. The study site will maintain a confidential list of subjects who participated in the study, linking each subject's numerical code to his or her actual identity. In case of data transfer, the Sponsor will protect the confidentiality of subjects' personal data consistent with the clinical study agreement and applicable privacy laws.

10.4. Study and Site Closure

The Sponsor designee reserves the right to close the study site or terminate the study at any time for any reason at the sole discretion of the Sponsor. Study sites will be closed upon study completion. A study site is considered closed when all required documents and study supplies have been collected and a study-site closure visit has been performed.

The investigator should initiate study-site closure at any time upon notification to the Sponsor if requested to do so by the responsible EC or if such termination is required to protect the health of subjects.

Reasons for the early closure of a study site by the Sponsor may include but are not limited to:

- Failure of the investigator to comply with the protocol, the requirements of the EC or local health authorities, the Sponsor's procedures, or GCP guidelines,
- Inadequate recruitment of subjects by the investigator,
- Discontinuation of further study drug development.

Study termination is also provided for in the clinical study agreement. If there is any conflict between the contract and this protocol, the contract will control as to termination rights.

11. DATA HANDLING

11.1. Source Documents

Source documents provide evidence for the existence of the subject and substantiate the integrity of the data collected. Source documents are filed at the study site.

Data entered on the eCRF transcribed from source documents must be consistent with the source documents or the discrepancies must be explained. The investigator may need to request previous medical records or transfer records as required by the study. Also, current medical records must be available.

11.2. Electronic Case Report Form

An eCRF system provided by an independent third party will be used for data capture during the study. This system must be validated prior to use. Access at different levels to the system should be granted/revoked following Trinomab and/or vendor procedures, in accordance with regulatory and system requirements.

Data should be entered into the system after the subject has attended a visit. The investigator will approve the eCRF entries for each subject with an electronic signature which is equivalent to a handwritten signature.

Errors occurring in the eCRF will be corrected electronically. Such corrections/modifications will be automatically tracked by an audit trail detailing the date and time of the correction and the name of the person making the correction.

The eCRF system and the database will be hosted at the independent third party. After the trial database is declared clean and released to the statistician, a final copy of the database will be stored at Trinomab.

11.3. Data Management

A data management plan will be created under the responsibility of the Contract Research Organization (CRO) company. The data management plan will be issued before data collection begins and will describe all functions, processes, and specifications for data collection, cleaning and validation.

When all data have been processed, queries resolved, medical coding completed and any issues from review of protocol violations and data listings resolved, the database will be locked, and all update access removed.

11.4. Provision of Additional Information

On request, the investigator may need to provide the Sponsor with additional data relating to the study, or copies of relevant source records, duly anonymized. In case of particular issues or governmental queries, it may be necessary to have access to the complete study documents, provided that the subjects' confidentiality is protected in accordance with applicable requirements.

12. DATA QUALITY ASSURANCE

All subject data relating to the study will be recorded on eCRF unless transmitted to the Sponsor or designee electronically. The investigator is responsible for verifying that data entries are accurate and correct by physically or electronically signing the eCRF.

The investigator must maintain proper documentation (source data) that supports the information entered in the eCRF. The investigator must ensure that the eCRFs are securely stored at the study site in encrypted electronic form and are password protected to prevent access by unauthorized third parties.

The investigator must permit study-related monitoring, audits, EC review, and regulatory agency inspections and provide direct access to source data documents. It is important that the investigator(s) and their relevant personnel are available during the monitoring visits and possible audits or inspections and that sufficient time is devoted to the process.

Monitoring details describing strategy (eg, risk-based initiatives in operations and quality such as risk management and mitigation strategies and analytical risk-based monitoring), methods, responsibilities, and requirements, including handling of noncompliance issues and monitoring techniques (central, remote, or on-site monitoring), are provided in the monitoring plan.

Study monitors will perform ongoing source data verification to confirm that data entered into the eCRF by authorized site personnel are accurate, complete, and verifiable from source documents; that the safety and rights of subjects are being protected; and that the study is being

conducted in accordance with the currently approved protocol and any other study agreements, ICH GCP, and all applicable regulatory requirements.

After study completion, records and documents, including signed ICFs, pertaining to the conduct of this study must be retained by the investigator until 5 years after the marketing authorization unless local regulations or institutional policies require a longer retention period. No records may be destroyed during the retention period without the written approval of the Sponsor. No records or documents may be transferred to another location or party without written notification to the Sponsor. The investigator must ensure that the records and documents continue to be stored securely for as long as they are maintained.

When subject data are to be deleted, the investigator will ensure that all copies of such data are promptly and irrevocably deleted from all systems.

The investigator(s) will notify the Sponsor or its agent immediately of any regulatory inspection notification in relation to the study. Moreover, the investigator will cooperate with the Sponsor or its agent to prepare the investigator site for the inspection and will allow the Sponsor or its agent, whenever feasible, to be present during the inspection. The investigator site and investigator will promptly resolve any discrepancies that are identified between the study data and the subject's medical records. The investigator will promptly provide copies of the inspection findings to the Sponsor or its agent. Before response submission to the regulatory authorities, the investigator will provide the Sponsor or its agent with an opportunity to review and comment on responses to any such findings.

13. REPORTING AND PUBLICATION

13.1. Clinical Study Report

The data and information collected during this study will be reported in a clinical study report prepared by Trinomab and submitted for comments and signature to the signatory investigator(s).

13.2. Confidentiality and Ownership of Trial Data

Any confidential information relating to the study drug or the study, including any data and results from the study will be the exclusive property of Trinomab. The investigator and any other persons involved in the study will protect the confidentiality of this proprietary information belonging to Trinomab.

13.3. Publication Policy

At the end of the study, one or more manuscripts for joint publication may be prepared in collaboration between the investigator(s) offered authorship and Trinomab. In a multi-site study based on the collaboration of many sites, any publication of results must acknowledge all sites. Results from multi-site studies must be reported in a responsible and coherent manner and results from subsets should not be published in advance or without clear reference to the primary publication of the entire study.

Authorship will be granted based on International Committee of Medical Journal Editors (ICMJE) standards (see current official version: http/www.ICMJE.org). The total number of

authors is based on the guideline from the relevant journal or congress. In the event of any disagreement in the content of a publication, both the Investigator's and Trinomab's opinion will be fairly and sufficiently represented in the publication.

Any external CRO or laboratory involved in the conduct of this study has no publication rights regarding this study.

If the investigator wishes to independently publish/present any results from the study, the draft manuscript/presentation must be submitted in writing to Trinomab for comment prior to submission. Comments will be given within four weeks from receipt of the draft manuscript. This statement does not give Trinomab any editorial rights over the content of a publication, other than to restrict the disclosure of Trinomab's intellectual property. If the matter considered for publication is deemed patentable by Trinomab, scientific publication will not be allowed until after a filed patent application is published. Under such conditions the publication will be modified or delayed at the Investigator's discretion, to allow sufficient time for Trinomab to seek patent protection of the invention.

ICMJE member journals have adopted a trials-registration policy as a condition for publication. This policy requires that all clinical trials be registered in a public, clinical trials registry. Thus, it is the responsibility of Trinomab to register the trial in an appropriate registry, i.e.http://www.ClinicalTrials.gov which is Sponsored by the United States National Institutes of Health and http://www.cde.org.cn which is designated by NMPA.

14. REFERENCES

1. Wang Chuanlin, et al. Guidelines for the use of post-traumatic tetanus vaccines and passive immune preparation. Chinese Journal of Preventive Medicine, 2019 (12): 1212-1217.

2. Roper MH, Vandelaer JH, Gasse FL. Maternal and neonatal tetanus. Lancet, 2007, 370(9603):1947-1959.

3. Tetanus vaccines: WHO position paper - February 2017. Wkly Epidemiol Rec, 2017. 92(6): p. 53-76.

4. Yu Chao, Xu Yuming, Xu Jin, et al. Progress of clinical application and safety of tetanus antitoxin. Chinese Journal of Pharmacovigilance, 2016, 13 (1): 36-41.

5. Perey B J F. Progress in tetanus prophylaxis: the advent of human antitoxin. Canadian Medical Association Journal, 1966, 94(9): 437.

6. Srivastava P, B.K.C.J., Trends in tetanus epidemiology in the United States, 1972-2001. 39th National Immunization Conference Workshop 27, 2005. Available at: <http://cdc.confex.com/cdc/nic2005/techprogram/paper_7813.htm>. 2005.

7. Plotkin, S.A., W.A. Orenstein and P.A. Offit, Plotkin's vaccines. Seventh edition. ed. 2018. 1691.

8. WHO Immunological Basis for Immunization Series Module3-Tetanus. 2018.

9. Forrat, R., et al., Evaluation of the safety and pharmacokinetic profile of a new, pasteurized, human tetanus immunoglobulin administered as sham, postexposure prophylaxis of tetanus. Antimicrob Agents Chemother, 1998. 42(2): 298-305

15. APPENDICES AND ANNEXES

Appendix 1: Wound Exposure Category

The following categories are based on different wound exposure.

A complete medical history of the subject including the exact course of the injury and the environmental condition should be obtained for the classification:

a) Clean wounds: simple wounds located in areas of the body with minimal bacterial colonization, and promptly treated after injury (such as a cut from a blade);

b) Unclean wounds: wounds located in areas of the body with high bacterial colonization (e.g., armpits, groin, and perineum) or simple wounds that have not been treated within 6 hours of injury;

c) Contaminated wounds: wounds contaminated by dirt, organic soil (e.g., swamp or jungle soil), feces, or saliva (e.g., animal or human bites), wounds that are already infected, or wounds containing necrotic tissue (e.g., necrosis or gangrene), gunshot wounds, frostbite, burns, etc.

Appendix 2: Diagnostic Basis for Non-neonatal Tetanus:

According to the *Specifications for Diagnosis and Treatment of Non-neonatal Tetanus*, the diagnosis of non-neonatal tetanus is based on typical clinical manifestations with at least one of the following: (1) trismus or risus sardonicus; (2) painful muscle spasms.

For subjects with suspected diagnosis, the tongue blade test can be used. The method involves lightly touching the patient's posterior pharynx with a tongue blade, and a positive result is indicated by the occurrence of a reflexive contraction of the jaw muscles rather than the normal reflexive gagging. This method shows high sensitivity (94%) and specificity (100%).

Appendix 3: Abbreviations

The following is a list of abbreviations that may be used in the protocol.

Abbreviation	Definition
ADA	Anti-drug antibody
AE	Adverse events
AESI	Adverse events of special interest
ALT	Alanine aminotransferase
AST	Aspartate aminotransferase
AUC_{0-last}	Area under the plasma concentration-time curve from time zero to the last measurable concentration
CI	Confidence interval
CIOMS	Council for International Organizations of Medical Sciences
CL/F	Apparent clearance
C_{max}	Maximum concentration
CRF	Case report form
eCRF	Electronic case report form
CRO	Contract research organization
EC	Ethics Committee
ECG	Electrocardiogram
$F(ab')_2$	Equine anti-tetanus immunoglobulin
FAS	Full analysis set
GCP	Good Clinical Practice
GMP	Good Manufacturing Practice
HED	Human equivalent dose
HTIG	Human tetanus immunoglobulin
IAS	Immunogenicity set
IB	Investigator's brochure
ICF	Informed consent form
ICH	International Council for Harmonisation
ICMJE	International Committee of Medical Journal Editors

Abbreviation	Definition
ICR	Institute of Cancer Research
Ig	Immunoglobulin
IM	Intramuscular injection
IRT	Interactive Response Technology
IV	Intravenous infusion
IWR	Interactive Web-based Response
LD50	Lethal dose, 50%
mAb	Monoclonal antibody
MedDRA	Medical Dictionary for Regulatory Activities
MMRM	Mixed-effect model for repeated measures
MTD	Maximum tolerated dose
NAb	Neutralizing antibody
NMPA	National Medical Products Administration
NOAEL	No observed adverse effect level
NONMEM	Nonlinear mixed effect model
PD	Pharmacodynamics
PK	Pharmacokinetics
PKS	Pharmacokinetic set
PT	Prothrombin
PT	Preferred term
SAE	Serious adverse event
SAP	Statistical analysis plan
SoA	Schedule of Activities
SOC	System organ class
SS	Safety set
SUSAR	Suspected Unexpected Serious Adverse Reaction
$t_{1/2}$	Half-life
TAT	Tetanus antitoxin
TEAE	Treatment emergent adverse event

**Abbreviation**	**Definition**
TIG	Tetanus immunoglobulin
T_{max}	Time to maximum concentration
$V_{d,ss}$	Volume of distribution at steady state
WHO	World Health Organization
WOCBP	Women of childbearing potential

Annex 1: Package Insert for Tetanus Human Immunoglobulin

Annex 2: Package Insert for Adsorbed Tetanus Vaccine

Statistical Analysis Plan

Protocol title: A Multicenter, Randomized, Double-Blind, Parallel-Group, Active-Controlled Phase III Study to Compare the Efficacy and Safety of Passive Immunization with TNM002 Injection and Human Tetanus Immunoglobulin as Prophylaxis Against Tetanus

Statistical Analysis Plan: Final
Statistical Analysis Plan: 2.0
Statistical Analysis Plan Date: 30AUG2023

Investigational Product: TNM002

Protocol Reference: TNM002-301
[redacted]
Sponsor: TRINOMAB BIOTECH CO., LTD.

Author:
[redacted]
SENIOR BIOSTATISTICIAN

Table of Contents

Glossary of Abbreviations 6
1. Source Documents 7
2. Background 8
3. Protocol Details 9
3.1. Total Study Design 9
3.2. Study Objectives 10
3.2.1. Primary Efficacy Objective(s) 10
3.2.2. Secondary Efficacy Objective(s) 10
3.2.3. Safety Objective(s) 10
3.2.4. Exploratory Objective(s) 10
3.3. Sample Size and Power 10
4. Endpoints 12
4.1. Primary Efficacy Endpoint 12
4.2. Secondary Efficacy Endpoint 12
4.3. Other Efficacy Endpoints 12
4.4. Safety Endpoints 12
4.5. Immunogenicity Endpoints 12
4.6. Pharmacokinetic Endpoints 13
5. Estimands 13
5.1. Estimand(s) for Primary Endpoint 13
5.1.1. Treatment Condition of Interest 13
5.1.2. Population of Subjects Targeted by the Clinical Question 13
5.1.3. Variable Obtained from Each Subject Required to Address the Clinical Question13
5.1.4. Handling of Intercurrent Events to Reflect the Clinical Question of Interest 14
5.1.5. Population-level Summary for Comparison between Treatment Conditions 14
5.1.6. Sensitivity Analysis for Primary Estimand 14
5.1.7. Supplementary Analyses for Primary Estimand 14
5.2. Estimand(s) for Secondary Endpoint 15
5.2.1. Treatment Condition of Interest 15

5.2.2. Population of Subjects Targeted by the Clinical Question 15
5.2.3. Variable Obtained from Each Subject Required to Address the Clinical Question15
5.2.4. Handling of Intercurrent Events to Reflect the Clinical Question of Interest 15
5.2.5. Population-level Summary for Comparison between Treatment Conditions 16
5.2.6. Sensitivity Analysis for Secondary Estimand 16
5.2.7. Supplementary Analyses for the Secondary Estimand 16
6. Analysis Populations 16
6.1. All-Screened Set 16
6.2. Intention-To-Treat (ITT) Population 16
6.3. Full Analysis Set (FAS) 17
6.4. Per Protocol Set (PPS) 17
6.4.1. Important Protocol Deviations Leading to Exclusion from the PPS 17
6.5. Safety Set (SS) 17
6.6. Immunogenicity Analysis Set (IAS) 17
6.7. Pharmacokinetic Set (PKS) 18
7. Data Handling 18
7.1. Time Points and Visit Windows 18
7.1.1. General Definitions 18
7.1.2. Baseline 18
7.1.3. Visit Windows 18
7.2. Calculation of Δ titers 18
7.3. Handling of Dropouts, Missing Data, and Outliers 19
7.3.1. Handling of Missing Efficacy Data 19
7.3.2. Handling of Missing Safety Data 19
7.3.3. Handling of Partial and Missing Dates for Prior / Concomitant Medications and Surgeries/Procedures 19
7.3.4. Handling of Serum Concentrations that are Below the Lower Limit of Quantification(BLQ) 20
8. Statistical Methods 22
8.1. General Principles 22
8.2. Subject Disposition and Data Sets Analyzed 22

8.3. Protocol Deviations 23
8.4. Demographic and Other Baseline Characteristics 23
8.4.1. Demographic and Baseline Characteristics 23
8.4.2. Medical History 25
8.4.3. Prior and Concomitant Medications 25
8.4.4. Prior and Concomitant Surgeries/Procedures 26
8.5. Measurements of Treatment Compliance 27
8.6. Efficacy 27
8.6.1. Primary Efficacy Analysis 27
8.6.2. Sensitivity for the Primary Efficacy Analyses 28
8.6.3. Supplementary for Primary Efficacy Analyses 28
8.6.4. Secondary Efficacy Analysis 29
8.6.5. Other Efficacy Analyses 29
8.7. Safety 32
8.7.1. Adverse Events 32
8.7.2. Laboratory Evaluations 34
8.7.3. Vital Signs 35
8.7.4. Electrocardiograms 36
8.7.5. Physical Examination 37
8.7.6. Primary Analysis and Data Monitoring Committee (DMC) 37
8.8. Pharmacokinetics Analysis 38
8.8.1. Presentation of Pharmacokinetic Concentration Data 38
8.9. Immunogenicity Analysis 39
9. Changes in the Conduct of the Study or Planned Analysis 39
10. References 40
11. Appendices 41

Reviewers

The following reviews of the Statistical Analysis Plan (SAP) were conducted:

[redacted]	[redacted]	[redacted]	[redacted]
[redacted]	[redacted]	[redacted]	[redacted]
[redacted]	[redacted]	[redacted]	[redacted]
[redacted]	[redacted]	[redacted]	[redacted]
[redacted]	[redacted]	[redacted]	[redacted]
[redacted]	[redacted]	[redacted]	[redacted]
[redacted]	[redacted]	[redacted]	[redacted]
[redacted]	[redacted]	[redacted]	[redacted]
[redacted]	[redacted]	[redacted]	[redacted]
[redacted]	[redacted]	[redacted]	[redacted]
[redacted]	[redacted]	[redacted]	[redacted]
[redacted]	[redacted]	[redacted]	[redacted]
[redacted]	[redacted]	[redacted]	[redacted]
[redacted]	[redacted]	[redacted]	[redacted]
[redacted]	[redacted]	[redacted]	[redacted].

Glossary of Abbreviations

Abbreviation	Definition
ADA	Anti-drug antibody
AE	Adverse event
AESI	Adverse event of special interest
ALT	Alanine aminotransferase
AST	Aspartate aminotransferase
BLQ	Below the lower limit of quantification
CI	Confidence interval
CV	Coefficient of variation
CS	Clinically significant
eCRF	Electronic case report form
ECG	Electrocardiogram
FAS	Full analysis set
GMT	Geometric mean titer
HTIG	Human tetanus immunoglobulin
IAS	Immunogenicity Analysis Set
IB	Investigator's brochure
ICF	Informed consent form
IM	Intramuscular
MedDRA	Medical Dictionary for Regulatory Activities
MMRM	Mixed-effect model for repeated measures
NAb	Neutralizing antibody
NCS	Not clinically significant
PE	Physical Examination
PK	Pharmacokinetic
PKS	Pharmacokinetic set
PT	Preferred term
SAE	Serious adverse event
SAP	Statistical analysis plan
SI	International System of Units
SoA	Schedule of Activities
SOC	System organ class
SS	Safety Set
TEAE	Treatment emergent adverse event
VS	Vital Sign

1. Source Documents

The SAP was written based on the following documentation:

Document	Date	Version
Protocol	16Jun2023	3.0
eCRF	21Feb2023	2.0

2. Background

This Statistical Analysis Plan (SAP) is based on Protocol TNM002-301 “A Multicenter, Randomized, Double-Blind, Parallel-Group, Active-Controlled Phase III Study to Compare the Efficacy and Safety of Passive Immunization with TNM002 Injection and Human Tetanus Immunoglobulin (HTIG) as Prophylaxis Against Tetanus”. This SAP provides details of the planned analyses and statistical methods for TNM002-301. This SAP should be read in conjunction with the study protocol and electronic case report forms (eCRFs). Any analyses that are beyond those outlined in the protocol are delineated in this document.

3. Protocol Details

3.1. Total Study Design

This is a Phase III, multicenter, randomized, double-blind, parallel-group, active-controlled study to compare the efficacy and safety of TNM002 injection (hereinafter referred to as TNM002) 10 mg and human tetanus immunoglobulin (HTIG) 250 IU as prophylaxis against tetanus in subjects with dirty or contaminated wounds caused by various injury and who require passive immunizing agents against tetanus.

Approximately 675 subjects are planned to be enrolled in this study. These subjects will be randomized and receive a single intramuscular (IM) gluteal injection of TNM002 10 mg (450 subjects) or HTIG 250 IU (225 subjects) in a 2:1 ratio. Randomization will be stratified by with or without concomitant administration of absorbed tetanus vaccine as determined before randomization. The proportion of subjects with concomitant absorbed tetanus vaccine administration will be approximately 10%.

The study personnel responsible for drug preparation and administration will be unblinded due to the apparent difference in the dosing volumes of TNM002 (0.5 mL) and HTIG (2.5 mL). During the study drug administration, measures should be taken to block subject's line of sight, in order to ensure that the subject cannot observe any identifying information of the study drug. The injection time of all study drugs should be controlled within 10 (± 1) seconds. The subjects, investigators, and the other study personnels at the study sites will be blinded throughout the study.

Before and after study drug administration, wound management should be performed in accordance with the clinical standard procedure. Concomitant medicine or other treatment required for injury itself or comorbidities are permitted during the study.

Serum samples for anti-tetanus neutralizing antibody and pharmacokinetic (PK) will be collected from all subjects at pre-dose, 12 hours, and 3, 7, 28 and 90 days after dosing. Serum samples for immunogenicity tests will be collected from all subjects at pre-dose, and 7, 28 and 90 days after dosing. These tests will be performed at a third party bioassay laboratory.

During the visits, subjects will also undergo safety assessments including collection of AEs, physical examinations (PE), vital signs (VS), 12-lead ECGs, and safety laboratory tests (hematology, clinical chemistry, coagulation, and urinalysis), and the tetanus occurrence will also be monitored. The last visit for this study is planned to be performed at 105 days after dosing and will be completed by telephone contact.

As the diagnosis of tetanus is mainly based on clinical symptoms and no reliable objective diagnostic criteria are available, a clinical adjudication committee consisting of clinical

experts in the field of tetanus disease will be established. For subjects with tetanus-related symptoms, the final diagnosis will be made by experts in this committee after discussion.

The primary analysis is planned to be performed after the last enrolled subject has completed the visit at 28 days after dosing, in order to evaluate the efficacy and safety of the study drug. The analysis will be carried out by an independent unblinded team. Sponsor will be responsible for overseeing this team to ensure data analysis is carried out in line with SAP.The Sponsor will clearly document the personnel who are informed of the unblinded analysis result and the measures taken to maintain the double-blind status for the rest of the study. The investigator and, study site staff and all personnels should be kept as blind, and will remain blinded throughout the study.

3.2. Study Objectives

3.2.1. Primary Efficacy Objective(s)

- To compare the efficacy of TNM002 Injection 10 mg with HTIG 250 IU as prophylaxis against tetanus following a single IM injection.

3.2.2. Secondary Efficacy Objective(s)

- To compare the clinical outcomes after administration of TNM002 10 mg with HTIG 250 IU as prophylaxis against tetanus following a single IM injection.

3.2.3. Safety Objective(s)

- To compare the safety profile of TNM002 10 mg with HTIG 250 IU as prophylaxis against tetanus following a single IM injection.

3.2.4. Exploratory Objective(s)

- To compare the protective antibody level of TNM002 10 mg with HTIG 250 IU as prophylaxis against tetanus at different time points following a single IM injection.
- To compare the long-term clinical outcomes after administration of TNM002 10 mg with HTIG 250 IU as prophylaxis against tetanus following a single IM injection.
- To evaluate the PK properties of TNM002 in subjects requiring tetanus passive immunization.
- To evaluate the immunogenicity of TNM002 in subjects requiring tetanus passive immunization.

3.3. Sample Size and Power

Approximately 675 subjects will be randomized such that approximately 540 evaluable subjects will complete the study.

Primary Hypothesis:

The hypothesis is that TNM002 has efficacy that is superior to HTIG as prophylaxis against tetanus. To be more specific, the lower limit of the 95% confidence interval (CI) of the difference (TNM002 group - HTIG group) in the proportion of subjects with Δtiters ≥0.01 IU/mL at 12 hours after the receipt of study drug is >0.

The sample size estimation is based on the above hypothesis and following assumptions: (1) The proportion of subjects with Δ titers ≥0.01 IU/mL at 12 hours after receipt of TNM002 and HTIG based on results of earlier clinical trials; (2) Due to the fact that HTIG is a well-studied product whose efficacy and safety does not need to be evaluated in large amount of subjects in a clinical trial setting, the randomization ratio between TNM002 and HTIG groups is 2:1; (3) One-sided Type I error of 0.025; (4) TNM002 requires adequate exposure during clinical development to fully understand its safety.

Taken together, the estimated sample size is approximately 540 subjects, i.e., 360 in the TNM002 group and 180 in the HTIG group. This will provide a power of >99% to evaluate the above efficacy hypothesis. The dropout rate is expected to be 20% in this study. Therefore, the planned enrollment will be 675 subjects, i.e., 450 in the TNM002 group and 225 in the HTIG group.

Secondary Hypothesis:

The tetanus protection rate is assumed to be 99.99% at 28 days post-dose in the HTIG group and 99.999% at 28 days post-dose in the TNM002 group. The current sample size will not provide sufficient power to test a formal statistical hypothesis in terms of tetanus protection rate between TNM002 and HTIG.

Safety Hypothesis:

Table 1 shows the probability of observing at least 1 AE for a given true event rate of a particular AE, for various sample sizes. For example, if the true AE rate is 1%, there is a 95.1% probability of observing at least 1 AE in 300 subjects.

Table 1: Probability of Observing at Least One Adverse Event at Different Sample Sizes and Different True Incidences of Adverse Events

Assumed True Incidence of AEs	Probability of observing at least one AE at different sample sizes			
	N=200	N=300	N=500	N=600
0.10%	18.0%	25.9%	39.4%	45.2%
0.50%	63.3%	77.8%	91.8%	95.1%
1.00%	86.6%	95.1%	>99.0%	>99.0%
2.00%	98.2%	>99.0%	>99.0%	>99.0%
3.00%	>99.0%	>99.0%	>99.0%	>99.0%

Assumed True Incidence of AEs	Probability of observing at least one AE at different sample sizes			
	N=200	N=300	N=500	N=600
4.00%	>99.0%	>99.0%	>99.0%	>99.0%
5.00%	>99.0%	>99.0%	>99.0%	>99.0%
6.00%	>99.0%	>99.0%	>99.0%	>99.0%
7.00%	>99.0%	>99.0%	>99.0%	>99.0%
8.00%	>99.0%	>99.0%	>99.0%	>99.0%
9.00%	>99.0%	>99.0%	>99.0%	>99.0%
10.00%	>99.0%	>99.0%	>99.0%	>99.0%

4. Endpoints

4.1. Primary Efficacy Endpoint

- The increase of anti-tetanus neutralizing antibody titers from baseline at 12 hours after receipt of study drug

4.2. Secondary Efficacy Endpoint

- Tetanus protection rate within 28 days after receipt of study drug

Tetanus protection rate is defined as 1 - tetanus incidence within 28 days after receipt of study drug.

4.3. Other Efficacy Endpoints

- The increase of anti-tetanus neutralizing antibody titers from baseline at 3, 7, 28 and 90 days after receipt of study drug
- Tetanus protection rate within 90 and 105 Days after receipt of study drug

4.4. Safety Endpoints

- AEs
- SAEs
- Laboratory tests, vital signs, physical examinations, and electrocardiograms (ECGs)

4.5. Immunogenicity Endpoints

- Anti-drug antibodies (ADA) in subjects following TNM002 administration

4.6. Pharmacokinetic Endpoints

- Serum TNM002 concentrations in subjects following TNM002 administration.

5. Estimands

The ICH E9 (R1) addendum on estimands[2] and sensitivity analysis in clinical trials to the guideline on statistical principles for clinical trials came into effect on 30 July 2020. This section addresses the construction of estimands for the primary and secondary objectives. Each estimand is defined according to the following five attributes:

1) The **treatment** condition of interest, and as appropriate, the alternative condition to which comparison will be made.
2) The **population** of subjects targeted by the clinical question.
3) The **variable** (or endpoint) to be obtained for each subject that is required to address the clinical question.
4) The clinical question of interest in respect of **other intercurrent events** not covered through the precise specifications of treatment, population and variable.
5) A **population-level summary** for the variable providing a basis for comparison between treatment conditions.

5.1. Estimand(s) for Primary Endpoint

The primary estimand is the difference in proportions between TNM002 10 mg and HTIG 250 IU with an increase in anti-tetanus neutralizing antibody titers from baseline (Δ titers) ≥0.01 IU/mL at 12 hours after receipt of study drug, in subjects as defined by the enrollment criteria.

The main estimand is defined through the following five attributes:

5.1.1. Treatment Condition of Interest

The primary treatment condition of interest is a single IM gluteal injection of TNM002 10 mg; compared against the alternative treatment condition of HTIG 250 IU.

5.1.2. Population of Subjects Targeted by the Clinical Question

The population targeted by the clinical question is defined through the inclusion and exclusion criteria as part of the clinical trial protocol 3.0. It will be analyzed in all randomized subjects who received study drug.

5.1.3. Variable Obtained from Each Subject Required to Address the Clinical Question

For each subject in the study, the variable to address the clinical question is a binary response variable of an increase of anti-tetanus neutralizing antibody titers (Δ titers) ≥0.01 IU/mL from baseline at 12 hours after receipt of study drug.

5.1.4. Handling of Intercurrent Events to Reflect the Clinical Question of Interest

The following table describes how intercurrent events will be collected and handled within the analysis.

Table 2 Handling of Intercurrent Events for the Primary Estimand

Intercurrent event	Data collection and analysis
AE leading to discontinuation from study prior to the 12 hours after dosing	The event will be integrated into the non-response variable (Δ titers <0.01 IU/mL) in line with a composite strategy.
Important protocol deviations that affect neutralizing antibody titers prior to the 12 hours after dosing	Subjects will be followed and data collected after the intercurrent event will be used in analysis in line with a treatment-policy strategy.
Receiving additional passive immunizing agents for tetanus due to reasons unrelated to the original wound prior to the 12 hours after dosing	Subjects will be followed and data collected after the intercurrent event will be used in analysis in line with a treatment-policy strategy.
Receiving additional passive immunizing agents for tetanus due to reasons related to the original wound prior to the 12 hours after dosing	The event will be integrated into the non-response variable (Δ titers <0.01 IU/mL) in line with a composite strategy.

5.1.5. Population-level Summary for Comparison between Treatment Conditions

The treatment effect will be quantified by evaluating the differenceof proportions of subjects with an increase in anti-tetanus neutralizing antibody titers (Δ titers) ≥0.01 IU/mL from baseline at 12 hours after receipt of TNM002 and HTIG.

5.1.6. Sensitivity Analysis for Primary Estimand

The sensitivity analysis for primary estimand of the difference in the proportions between the TNM002 and HTIG groups and its two-sided 95% CI will be performed by stratified Newcombe Wilson method instead of the main analysis of stratified Miettinen-Nurminen method.

5.1.7. Supplementary Analyses for Primary Estimand

The following table describes how intercurrent events will be handled within the primary supplementary analysis.

Table 3 Handling of Intercurrent Events for the Supplementary Analyses for Primary Estimand

Intercurrent event	Data collection and analysis
AE leading to discontinuation of study prior to the 12 hours after dosing	While on treatment strategy, exclude values after discontinuation of study.
Important protocol deviations that affect neutralizing antibody titers prior to the 12 hours after dosing	While on treatment strategy, exclude values after the important protocol deviation occurred.
Receiving additional passive immunizing agents for tetanus due to reasons unrelated to the original wound prior to the 12 hours after dosing	While on treatment strategy, exclude values after additional passive immunizing agents for tetanus received.
Receiving additional passive immunizing agents for tetanus due to reasons related to the original wound prior to the 12 hours after dosing	While on treatment strategy, exclude values after additional passive immunizing agents for tetanus received.

5.2. Estimand(s) for Secondary Endpoint

The secondary estimand is the difference between TNM002 10 mg and HTIG 250 IU in tetanus protection rate within 28 days after receipt of study drug, in subjects as defined by the enrollment criteria.

5.2.1. Treatment Condition of Interest

The secondary treatment condition of interest is a single IM gluteal injection of TNM002 10 mg; compared against the alternative treatment condition of HTIG 250 IU.

5.2.2. Population of Subjects Targeted by the Clinical Question

The population targeted by the clinical question is defined through the inclusion and exclusion criteria as part of the clinical trial protocol 3.0.

5.2.3. Variable Obtained from Each Subject Required to Address the Clinical Question

For each subject in the study, the variable to address the clinical question is a binary response variable of without tetanus occurrence within 28 days after receipt of study drug.

5.2.4. Handling of Intercurrent Events to Reflect the Clinical Question of Interest

The following table describes how intercurrent events will be handled within the analysis.

Table 4 Handling of Intercurrent Events for the Secondary Estimand

Intercurrent event	Data collection and analysis
AE leading to discontinuation of study prior to 28 days after receipt of study drug	Discontinuation of study will be ignored (treat as missing data) in line with a treatment-policy strategy.
Important protocol deviations that affect tetanus occurrence prior to 28 days after receipt of study drug	Subjects will be followed and data collected after the intercurrent event will be used in analysis in line with a treatment-policy strategy.
Receiving additional passive immunizing agents for tetanus due to reasons unrelated to the original wound prior to 28 days after receipt of study drug	Subjects will be followed and data collected after the intercurrent event will be used in analysis in line with a treatment-policy strategy.
Receiving additional passive immunizing agents for tetanus due to reasons related to the original wound prior to 28 days after receipt of study drug	Subjects will be followed and data collected after the intercurrent event will be used in analysis in line with a treatment-policy strategy.

5.2.5. Population-level Summary for Comparison between Treatment Conditions

The treatment effect will be quantified by evaluating thetetanus protection rate difference within 28 days after receipt of TNM002 and HTIG,where, tetanus protection rate = 1 - tetanus incidence.

5.2.6. Sensitivity Analysis for Secondary Estimand

The sensitivity analysis for secondary estimand of the difference in tetanus protection rate between the TNM002 and HTIG groups and its two-sided 95% CI will be performed by stratified Newcombe Wilson method instead of the main analysis of stratified Miettinen-Nurminen method.

5.2.7. Supplementary Analyses for the Secondary Estimand

Supplementary analyses are not considered for secondary estimand.

6. Analysis Populations

6.1. All-Screened Set

The All-Screened Set will include every subject who has signed the informed consent form. The All-Screened Set will be used for summaries of disposition and the associated listing.

6.2. Intention-To-Treat (ITT) Population

All subjects randomized will be included in the ITT Population. ITT Population are analyzed according to their randomized treatment.

6.3. Full Analysis Set (FAS)

All randomized subjects who received the study drug. FAS subjects are analyzed according to their randomized treatment.

6.4. Per Protocol Set (PPS)

The PPS will consist of all subjects in the FAS who do not have any important protocol deviations considered to have a major effect on primary efficacy leading to exclusion from the PPS. PPS subjects are analyzed according to their randomized treatment.

Protocol deviations are defined as any change, divergence, or departure from the study design or procedures defined in the study protocol. Important protocol deviations are a subset of protocol deviations which may significantly impact the correctness, accuracy, and / or reliability of the study data or that may significantly affect a subject's rights, safety, or well-being. Section 6.4.1 details the deviations.

6.4.1. Important Protocol Deviations Leading to Exclusion from the PPS

Deviations from the protocol, as defined in the protocol and / or protocol deviation plan, will e documented by the study monitors and project management throughout the study period.

Only those important protocol deviations considered to have a major effect on primary efficacy will lead to complete exclusion of the subject from the PPS.

Criteria which require medical and/or clinical pharmacological interpretation will be reviewed prior to interim database lock for primary analysis and final database lock for final analysis. All important protocol deviations leading to exclusion from the PPS occurring during the study will be reviewed and approved by Sponsor prior to interim and final database lock.

6.5. Safety Set (SS)

All randomized subjects who received the study drug with at least one post-dose safety evaluation. SS subjects are analyzed according to the actual treatment they received.

6.6. Immunogenicity Analysis Set (IAS)

All randomized subjects who received TMN002 with at least one post-dose immunogenicity data. If the immunogenicity results are affected by protocol deviations, all or partial immunogenicity data of corresponding subjects will not be included in the IAS.

6.7. Pharmacokinetic Set (PKS)

All randomized subjects who received TNM002 with at least one post-dose evaluable PK concentration. If the PK concentration results are affected by important protocol deviations, all or partial PK data of corresponding subjects will not be included in the PKS. All important protocol deviations leading to exclusion from the PKS occurring during the study will be reviewed and approved by Investigators and Sponsor prior to interim and final database lock.

7. Data Handling

7.1. Time Points and Visit Windows

7.1.1. General Definitions

All assessment days will be related to the first day of treatment.

Day 1 is defined as the date of dosing of study drug. Relative days after Day 1 are calculated as (assessment date – Day 1 date) + 1. Relative days prior to Day 1 are calculated as (assessment date – Day 1 date). The day prior to Day 1 is Day -1. Day 0 is not defined.

The date of the dosing of study drug for each subject will be taken from the Study Drug Administration eCRF page.

7.1.2. Baseline

For all subjects, the baseline is defined as the period from informed consent to the dosing of study drug.

The baseline value will be defined as last scheduled or unscheduled value collected prior to the dosing of study drug. For post-baseline, only data from scheduled visits will be included in the summary tables.

7.1.3. Visit Windows

All data will be analyzed using nominal study visit as defined in the Study Schedule and eCRF. No visit windows will be applied for summary and analysis.

7.2. Calculation of Δ titers

Unless specified otherwise, Δ Titers in TNM002 group will be calculated by post-dose antibody titers - pre-dose antibody titers, both measured by TNM002 PD bioanalysis method; Δ Titers in HTIG group will be calculated by post-dose antibody titers - pre-dose antibody titers, both measured by HTIG PD bioanalysis method.

7.3. Handling of Dropouts, Missing Data, and Outliers

7.3.1. Handling of Missing Efficacy Data

Subjects with missing data for the primary efficacy endpoint of the increase of anti-tetanus neutralizing antibody titers from baseline at 12 hours after receipt of study drug will be treated as non-responders if missing data caused by AE leading to discontinuation of study prior to the 12 hours after dosing for the primary analysis. Additionally, subjects who receive additional passive immunizing agents for tetanus due to reasons related to the original wound prior to the 12 hours after dosing will also be deemed non-responders, regardless of any subsequent response recorded for the primary efficacy endpoint. Besides, subjects whose data is missing at some time points but without discontinuation from the study will be excluded from the analysis for the missing time points.

In addition, NR (Not Reported) will be reported for subjects in TNM002 group if the baseline antibody level over a certain value, and will exclude these subjects from analysis.

Subjects with missing data for the endpoint of tetanus protection rate at certain time point after receipt of study drug will be treated as without tetanus occurrence if the subject stayed in the study and continued to be tetanus free at later time points.

7.3.2. Handling of Missing Safety Data

In general, summary statistics will be reported for observed data only. Missing data will not be imputed in this study such as missing clinical laboratory data, vital signs data, ECG data and efficacy data. Adverse event imputations for missing severity or relationship are given in Section 8.7.1.2. Unknown or partial dates of medication and surgeries/procedures imputations are given below and to be used only for the assessment of prior/concomitant status for medications, surgeries/procedures.

7.3.3. Handling of Partial and Missing Dates for Prior / Concomitant Medications and Surgeries/Procedures

<u>Missing or Partial Prior / Concomitant Medication and Surgeries/Procedures Start Dates</u>

Missing and / or incomplete dates for medications, surgeries/procedures are imputed in a manner resulting in the earliest onset or the longest duration after the start of treatment, whilst ensuring that the start date does not occur after the stop date. The stop date will not be imputed if the medication is “Ongoing”. Technically, this will be done as follows:

For a missing / incomplete start date / time the earliest date / time of the following will be imputed:

- The later date of: the earliest possible start date / time, and the date / time of first dose of treatment.
- The latest possible start date / time.
- The latest possible stop date / time.

For a missing / incomplete stop date / time the later date / time of the following will be imputed:

- The earlier date / time of the latest possible stop date / time and the date / time of last dose of treatment.
- The earliest possible stop date / time.
- The earliest possible start date / time.

Here, the earliest possible date / time is defined as:

- The date / time of the first day of the month at 00:00hrs, if month and year are available but the day / time is missing.
- The date / time of the first day of the year at 00:00hrs, if year is available but day / time and month are missing.
- 00:00hrs on the day of informed consent, if the date / time is completely missing.

The latest possible date / time is defined as:

- The date / time of the last day of the month at 23:59hrs, if month and year are available but the day / time is missing.
- The date / time of the last day of the year at 23:59hrs, if year is available but day / time and month are missing.
- 23:59hrs on the date of last known date on the study for the subject plus one year, if the date / time is completely missing.

7.3.4. Handling of Serum Concentrations that are Below the Lower Limit of Quantification(BLQ)

7.3.4.1. Pharmacokinetic Analysis

Serum concentrations that are below the lower limit of quantification (BLQ) will be handled as follows for descriptive statistics:

- Values that are BLQ will be set to 0 for the calculation of summary statistics.
- Arithmetic mean or median values that are BLQ will be presented as 0.
- If any BLQ results (treated as 0) are in a series of summarized data, geometric mean and coefficient of variation (CV) % of geometric mean will be reported as not calculated (NC).

7.3.4.2. Anti-tetanus Neutralizing Antibody Titers Analysis

Anti-tetanus neutralizing antibody titers that are below the lower limit of quantification (BLQ) will be handled as follows for descriptive statistics:

- Titers that are BLQ will be set to 0 for the calculation of summary statistics.
- Values of Δ titers ≤0 will be set to 0 for the calculation of summary statistics. Descriptive statistics of geometric mean and geometric CV% will be reported as not calculated (NC) when the treatment group contains 0.

8. Statistical Methods

8.1. General Principles

All data processing, summarization and analyses will be performed using [redacted] SAS Environment / Version 9.4 (or later) of the SAS® statistical software package.

The default summary statistics for continuous variables will be the number of non-missing observations (n), mean, standard deviation (SD), median, minimum (min) and maximum (max)， for those subjects with data, unless stated otherwise.

For categorical variables, the number (n) and percentage (%) of subjects with non-missing observations per category will be the default summary presentation, and where appropriate and present, the number of missing values as a “Missing” category. Number of subjects in the analysis population will be used as denominator for percentages calculation, unless stated otherwise in TFLs mock shell(s).

All statistical comparisons will be made using two sided tests at the p=0.05 significance level unless specifically stated otherwise. All null hypotheses will be of no treatment difference. All alternative hypotheses will be two-sided including calculating 95% CI. Specifications for table, figures and data listing formats can be found in the TFL shells specifications for this study.

8.2. Subject Disposition and Data Sets Analyzed

Subject disposition will be summarized by treatment group and overall, where appropriate, for the All-Screened Set, unless stated otherwise.

The following information will be reported:

- Number of subjects for the following categories:
    - Screened
- Number and percentage of subjects for the following categories:
    - Screen Failure,
    - Reason for screen failure,
    - Randomized,
    - Treated,
    - Not Treated,
    - Completed the study,
    - Discontinued the Study,
    - Reasons for study discontinuation.
- Number and percentage of subjects included in, and excluded from, each study population together with the reasons for exclusion from the analysis set;

- Number and percentage of subjects who met / did not meet all eligibility criteria, together with the criteria not met.
- Number and percentage of subjects by stratification factors. Each stratification variable will be presented by the stratum collected in IXRS. This will be repeated in the two subgroups by age group (≤65 vs >65).

The following subject data listings will be prepared as below:
A listing of all subjects with their study completion status, including the reasons for study discontinuation will be presented for the All-Screened Set.

A listing of all screen failed subjects with their reasons for screen failure will be presented for the All-Screened Set. A separate listing of subjects who failed at least one inclusion / exclusion criteria including a text description of the criterion failed will be presented for the All-Screened Set.

A listing of all randomized subjects with their randomization details, including dosing date and time, and actual treatment received will be presented for the ITT Population.

A listing of subject unblinding information will be presented for the ITT Population.

A listing of all subjects excluded from at least one analysis set will be presented for the ITT Population.

8.3. Protocol Deviations

All important protocol deviations will be summarized for the ITT Population by treatment group and overall as described below:

- The number of unique subjects with at least one important protocol deviation as well as the number of subjects in each important protocol deviation category will be presented by default descriptive summary statistics for categorical variables.

A listing of all subjects with one or more important / non important protocol deviations will be presented for the ITT Population.

8.4. Demographic and Other Baseline Characteristics

8.4.1. Demographic and Baseline Characteristics

Demographic characteristics will be summarized for the FAS by treatment group separately and overall as described below. All missing data will be presented as part of a missing category, if appropriate. Standard descriptive statistics will be presented for the continuous variables of:

- Age (years)
- Height (cm)
- Weight(kg)
- Body mass index (kg/m^2) [calculated as (body weight / height^2) where weight is in kg and height is in m] and presented to one decimal precision
- Anti-tetanus antibody titer at baseline (by HTIG PD bioanalysis method)

Anti-tetanus antibody titers will use the summary statistics including n, arithmetic mean, arithmetic SD, arithmetic CV%, median, min, max, GMT (geometric mean titer), geometric SD, geometric CV% , 95% CI of GMT, and proportion of BLQ subjects. If the GMT and geometric CV% could not be calculated due to subjects with values of anti-tetanus antibody titer containing BLQ (i.e., set to 0), the below sensitivity analysis will be performed as follow:

- LLOQ/2 of the corresponding detection method will be added to all values in the treatment group, and then the GMT and geometric CV% will be calculated. The final GMT and 95% CI of GMT will be calculated by subtracting LLOQ/2 of the corresponding detection method.

For the following category variables, the total counts and percentage will be provided.

- Age group (years):
    - ≤65
    - >65 to ≤75
    - >75
- Gender
    - Male
    - Female
- Is the subject of childbearing potential? (female only)
    - Yes
    - No
- Ethnicity
    - Han Nationality
    - Other
- Race
    - Asian
    - Other
- Classification of anti-tetanus antibody titer at baseline
    - < 0.01 IU/mL

- ≥ 0.01 IU/mL

Besides, demographic characteristics will be repeated by two subgroups based on the use of tetanus vaccine as determined before randomization, and by two subgroups based on age group (≤65 vs >65).

Demographic characteristics will be listed for the FAS.

8.4.2. Medical History

Medical history refers to the information of prior or concomitant diseases that exist prior to dosing, including history of infectious diseases, surgery, injury and allergy.

Medical history will be coded using the Medical Dictionary for Regulatory Activities (MedDRA)[version 25.1 or later] and will be presented by System Organ Class (SOC) and Preferred Term (PT). The SOCs and PTs are to be sorted by descending order of incidence in TNM002 group.

Medical history records will be summarized for the FAS by treatment group and overall as follows:

- The number and percentage of subjects with at least one medical history record will be presented.
- The number and percentage of subjects with at least one medical history record within each primary SOC and PT will be presented. The summary will be sorted using descending order of numerical counts in TNM002 group first and then HTIG group for both SOC and PT. Where terms tie, these will be sorted alphabetically.

Medical history records will be listed by-subject and within-subject by medical history start date for the FAS.

8.4.3. Prior and Concomitant Medications

All medications will be coded using the WHO Drug Global Dictionary, Format B3 [Version Sep 2022 (or a later version if updated during the study)], Anatomical Therapeutic Chemical (ATC) Classification codes.

Prior and concomitant medications are defined as follows:

- Prior medications are defined as medications that stopped before the date of dosing of study medication.
- Concomitant medications are defined as any medications taken on or after the dosing of study medication until the end of the study.

See Section 7.3.3 for imputation of missing or partial dates for medication.

Prior and concomitant medications will be summarized separately for the FAS by treatment group and overall as follows:

- The number and percentage of subjects with at least one prior / concomitant medication will be presented.
- The number and percentage of subjects with at least one prior / concomitant medication within each Anatomical Group (ATC Level 1), Therapeutic Subgroup (ATC Level 2), and preferred term will be presented. The summary will be sorted using numerical counts by descending order of Anatomical Group, then descending order of Therapeutic Subgroup, then descending order of preferred term in the TNM002 group first and then HTIG group. Where groups or terms tie these will be sorted alphabetically.

Prior medications and concomitant medications will be listed separately for the FAS. In the listings the relative start and stop day of prior / concomitant medication use will be calculated relative to the dosing date and time of treatment and will be presented for those subjects who received at least one dose of treatment. If the concomitant medication is "Ongoing" it will be indicated as such in the listing and the relative stop day will not be calculated. Only original dates will be presented in the listing even though the relative day may be based on an imputed date.

In addition, additional passive immunizing agents for tetanus and adsorbed tetanus vaccine administration will be coded using the WHO Drug Global Dictionary, Format B3 [Version Sep 2022 (or a later version if updated during the study)], Anatomical Therapeutic Chemical (ATC) Classification codes as well, and will be summarized separately for the FAS by treatment group and overall, similar to the analysis of prior and concomitant medications.

Tetanus vaccination history will be summarized using standard descriptive statistics for the FAS by treatment group separately and overall as well.

8.4.4. Prior and Concomitant Surgeries/Procedures

All surgeries/procedures will be coded using the Medical Dictionary for Regulatory Activities (MedDRA)[version 25.1 or later], System Organ Class (SOC) and Preferred Term (PT).

Prior and concomitant surgeries/procedures are defined as follows:

- Prior surgeries/procedures are defined as surgeries/procedures that stopped before the date of dosing of study medication.
- Concomitant surgeries/procedures are defined as any surgeries/procedures taken on or after the dosing of study medication until the end of the study.

See Section 7.3.3 for imputation of missing or partial dates for surgeries/procedures.

Prior and concomitant surgeries/procedures will be summarized separately for the FAS by treatment group and overall as follows:

- The number and percentage of subjects with at least one prior / concomitant surgeries/procedures will be presented.
  The number and percentage of subjects with at least one prior / concomitant surgeries/procedures within each primary SOC and PT will be presented. The summary will be sorted using numerical counts in the TNM002 group first and then HTIG group by descending order for SOC and PT. Where groups or terms tie these will be sorted alphabetically.

Prior surgeries/procedures and concomitant surgeries/procedures will be listed separately for the FAS. In the listings the relative start and stop day of prior / concomitant surgeries/procedures will be calculated relative to the dosing date and time of treatment and will be presented for those subjects who received at least one dose of treatment. If the concomitant surgery/procedure is "Ongoing" it will be indicated as such in the listing and the relative stop day will not be calculated. Only original dates will be presented in the listing even though the relative day may be based on an imputed date.

8.5. Measurements of Treatment Compliance

Not applicable for this study.

8.6. Efficacy

Efficacy analysis will be performed on the FAS.

8.6.1. Primary Efficacy Analysis

The primary efficacy variable is defined as binary response of increase of anti-tetanus neutralizing antibody titers (Δ titers) $\geq$ 0.01 IU/mL from baseline at 12 hours after receipt of study drug.

Subjects with missing data for the primary efficacy endpoint will be treated as non-responders if missing data caused by AE leading to discontinuation of study prior to the 12 hours after dosing for the primary analysis. Additionally, subjects who receive additional passive immunizing agents for tetanus due to reasons related to the original wound prior to the 12 hours after dosing will also be deemed non-responders, regardless of any subsequent response recorded for the primary efficacy endpoint. Besides, subjects whose data is missing at some time points but without discontinuation from the study will be excluded from the analysis for the missing time points.

The primary efficacy analysis can be summarized as follows:

1. Summarizing the proportion of subjects with an increase of anti-tetanus neutralizing antibody titers (Δ titers) $\geq$ 0.01 IU/mL from baseline at 12 hours after receipt of study drug and its two-sided 95% CI from FAS.

Clopper-Pearson method, which is an alternative to calculating binomial CI using normal approximation, will be used to calculate the 95% CI for each of the two treatment groups.

2. Calculating the difference of the proportion of subjects with an increase of anti-tetanus neutralizing antibody titers (Δ titers) ≥0.01 IU/mL from baseline at 12 hours after receipt of study drug and the corresponding 95% CI stratified by with or without concomitant administration of tetanus vaccine as determined before randomization.

   Stratified Miettinen-Nurminen method, will be used for constructing CI of the difference in binomial proportions between the TNM002 and HTIG groups from those stratified 2×2 samples.

Subjects who are diagnosed with tetanus or receive additional passive immunizing agents for tetanus due to reasons related to the original wound within 12 hours after receipt of study drug will be considered to have Δ titers < 0.01 IU/mL.

The above analysis will be repeated in subjects from FAS whose baseline antibody titers <0.01 IU/mL. If appropriate, the above analysis will be repeated in the two subgroups by age group (≤ 65 vs > 65).

8.6.2. Sensitivity for the Primary Efficacy Analyses

To assess the sensitivity of the results to the primary analysis, the difference in the proportions between the TNM002 and HTIG groups and its two-sided 95% CI (by Newcombe Wilson method stratified by with or without concomitant administration of tetanus vaccine as determined before randomization) will also be performed. The handling manner of intercurrent events is same as the primary estimand.

8.6.3. Supplementary for Primary Efficacy Analyses

8.6.3.1. Supplementary Analysis I

The handling manner of intercurrent events is same as the supplementary analyses for primary estimand in Table 3.

The analysis method is same as the primary analysis.

8.6.3.2. Supplementary Analysis II

To assess the robustness of the results to the primary analysis, the supplementary analysis will be performed using the PPS with observed data only.

8.6.4. Secondary Efficacy Analysis

8.6.4.1. Tetanus protection rate within 28 days after receipt of study drug

The secondary efficacy variable is defined as a binary response variable of without tetanus occurrence within 28 days after receipt of study drug.

Secondary efficacy analysis can be summarized as follows:

1. The tetanus protection rate at 28 days after receipt of study drug and its two-sided 95% CI in FAS will be calculated for each treatment group. The protection rate defined as 1− tetanus incidence.

   Clopper-Pearson method will be used to construct the 95% CI for each of those two treatment groups.

2. The difference in tetanus protection rate between the TNM002 and HTIG groups and its two-sided 95% CI will be calculated as well. The difference of 95% CI will be stratified by the use of tetanus vaccine as determined before randomization.

   Stratified Miettinen-Nurminen method, will be used for constructing CI of the difference in binomial proportions between the TNM002 and HTIG groups from those stratified 2×2 samples.

If appropriate, subjects will be divided into two subgroups based on the use of tetanus vaccine as determined before randomization, as well as in the two subgroups by age group (≤65 vs >65). The tetanus protection rate at 28 days after receipt of study drug and its two-sided 95% CI in FAS will be presented by treatment group and subgroup. Clopper-Pearson method will be used to construct the 95% CI for each treatment groups.

8.6.4.2. Sensitivity Analyses for the Secondary Efficacy Analysis

To assess the sensitivity of the results to the secondary analysis, the difference in tetanus protection rate between the TNM002 and HTIG groups and its two-sided 95% CI by Newcombe Wilson method stratified by with or without concomitant administration of tetanus vaccine as determined before randomization will also be performed.

8.6.5. Other Efficacy Analyses

The other efficacy endpoints will be analyzed using the FAS with observed data only.

8.6.5.1. Anti-tetanus neutralizing antibody titers at 3, 7, 28 and 90 days after receipt of study drug

The anti-tetanus neutralizing antibody titer analysis at 3, 7, 28 and 90 days after receipt of study drug will be performed in subjects from FAS that didn't receive tetanus vaccine up to the respective time point.

Subjects who were diagnosed with tetanus or receive additional passive immunizing agents for tetanus due to reasons related to the original wound before the respective time point are considered to have a titer = LLOQ/2 and a Δ titer <0.01 IU/mL at that time point.

8.6.5.1.1. Proportion of subjects with Δ titers ≥0.01 IU/mL from baseline at 3, 7, 28 and 90 days after receipt of study drug, respectively

Using the same methods described in Section 8.6.1, the proportion of subjects with Δ titers ≥0.01 IU/mL from baseline at 3, 7, 28 and 90 days after receipt of study drug and its two-sided 95% CI will be calculated for each treatment group. The difference in those proportions between the TNM002 and HTIG groups and its two-sided 95% CI will also be calculated. Miettinen-Nurminen method will be used for constructing CI of the difference of proportions between the TNM002 and HTIG groups.

If appropriate, the above analysis will be repeated in the two subgroups by age group (≤65 vs >65).

8.6.5.1.2. Geometric mean of Δ titers from baseline at 12 hours, 3, 7, 28 and 90 days after receipt of study drug, respectively

The anti-tetanus neutralizing antibody titers and Δ Titers will be summarized by treatment group at each scheduled time point using the summary statistics including n, arithmetic mean, arithmetic SD, arithmetic CV%, median, min, max, geometric mean, geometric SD, geometric CV% and 95% CI of geometric mean.

The analysis will be performed in subjects from FAS that haven't received tetanus vaccine up to the respective time point.

Individual and mean (including geometric and arithmetic) TNM002 titers - time profiles and Δ titers - time profiles will also be presented graphically, nominal collection time will be used in mean profile and actual collection time will be used in individual profile.

If the GMT and geometric CV% could not be calculated due to subjects with anti-tetanus antibody titer of BLQ (i.e., set to 0), the below sensitivity analysis will be performed:

- LLOQ/2 of the corresponding detection method will be added to all values in the treatment group , and then the GMT and geometric CV% will be calculated. The final GMT and 95% CI of geometric mean will be calculated by subtracting LLOQ/2 of the corresponding detection method.

If the geometric mean and geometric CV% could not be calculated due to the large number of visit contain values of Δ titers ≤0 (i.e., set to 0), two sensitivity analyses will be performed as follow:

- The geometric mean and geometric CV% will be calculated by setting 0 to LLOQ/4 of the corresponding detection method.
- LLOQ/2 of the corresponding detection method will be added to all values in the treatment group , and then the geometric mean and geometric CV% will be calculated. The final geometric mean and 95% CI of geometric mean will be calculated by subtracting LLOQ/2 of the corresponding detection method.

In addition, Δ titers at each scheduled time point after dosing will be compared and analyzed between treatment groups based on the mixed-effect model for repeated measures (MMRM) method. The MMRM model will use the log-transformed Δ titers at each scheduled time point as the dependent variable, the treatment group, time point, and treatment group-time point interaction as the independent variables, the the log-transformed baseline anti-tetanus neutralizing antibody titer as the covariate. Baseline anti-tetanus neutralizing antibody titers that are BLQ will be treated as LLOQ/2 for the purpose of log-transformation. The intra-subject variance-covariance structure will be the unstructured (UN). The difference in Δ titers and 95% CI of the difference between different treatment groups will be calculated and then antilog-transformed to obtain the ratio of GMT and CI of ratio. If the model does not converge when the covariance structure is UN, other possible covariance structures, such as Toelitz, autoregression (1) (AR (1)), etc., can be selected, and the covariance structure with the smallest final AIC value will be the standard to select the best for the final model.

The above analysis will be repeated in for subjects in TNM002 group by subgroups of ADA status. No statistical comparisons will be performed.

If appropriate, the above analysis will be repeated in the two subgroups by age group (≤65 vs >65). No statistical comparisons will be performed.

8.6.5.1.3. Antibody titers in the subgroup of subjects who received Tetanus vaccine as determined before randomization

In randomized subjects who received study drug and tetanus vaccine as determined before randomization, the proportion of subjects with Δ titers ≥0.01 IU/mL from baseline at 3, 7, 28 and 90 days after receipt of study drug and its two-sided 95% CI will be calculated for each treatment group using method described in section 8.6.1.

Antibody titers and Δ titers from baseline at 12 hours, 3, 7, 28 and 90 days after receipt of study drug will be summarized in the subgroup of FAS consisting of subjects who received tetanus vaccine as determined before randomization. Baseline and post-dose antibody titers by HTIG bioanalysis PD method will be used for HTIG group. Baseline and post-dose antibody titers by both TNM002 and HTIG bioanalysis PD method will be

presented for TNM002 group. Antibody titers and Δ titers will be summarized by treatment group and detection method at each scheduled time point using the summary statistics including n, arithmetic mean, arithmetic SD, arithmetic CV%, median, min, max, geometric mean, geometric SD, geometric CV% , 95% CI of geometric mean, the proportion of subjects with Δ titers ≥0.1 IU/mL and the proportion of subjects with antibody titers ≥0.1 IU/mL. Data handling rules, statistical methods and appropriate sensitivity analysis from previous sections will apply.

8.6.5.2. Tetanus protection rate within 90 and 105 Days after receipt of study drug

The tetanus protection rate within 90 and 105 days will be calculated in FAS for each treatment group respectively.

The protection rate differences at those two respective timepoints will be compared between TNM002 and HTIG groups stratified by the use of tetanus vaccine before randomization, same as the secondary analysis method.

Subjects will be divided into two subgroups based on the use of tetanus vaccine before randomization. Clopper-Pearson method will be used to construct the 95% CI for each treatment group. The difference in tetanus protection rate between the TNM002 and HTIG groups will be calculated and its two-sided 95% CI will be performed by Miettinen-Nurminen method. If appropriate, the above analysis will also be repeated in the two subgroups by age group (≤65 vs >65).

8.7. Safety

Safety analysis will be summarized for SS according to the study drug they actually received. Subjects will be divided into two subgroups based on the actual administration of tetanus vaccine and all the safety assessments will be repeated, except AEs leading to death or discontinuation of study and physical examination.

8.7.1. Adverse Events

If appropriate, the analysis described in this section will be repeated in the two subgroups by age group (≤65 vs >65).

8.7.1.1. Injection Site Assessment

An injection site reaction assessment should be performed 30 (± 5) minutes, 12 hours (± 30 mins) and 3 days (± 4 hours) after IM injection of the study drug. The number and percentage of injection site assessed and injection site reaction symptom will be summarized using default descriptive statistics by treatment group separately and overall at scheduled time points for the SS.

The injection site reaction assessment will be listed.

8.7.1.2. Other Adverse Events

All AEs recorded on the eCRF are TEAEs with start date and time after the start of treatment, and will be coded using the MedDRA dictionary [version 25.1 or higher].

- Treatment Emergent Serious AEs (TESAEs) will be defined as TEAEs regarded by the investigator as Serious Event= "Yes".
- The relationship between a TEAE and treatment is assessed as related, or not related. A treatment-related TEAE will be defined as a TEAE considered by the investigator or sponsor as related to treatment or with missing relationship to treatment.
- Assessment of AE severity will be based on the following guidelines:
  - Mild: asymptomatic or mild symptoms; clinical or diagnostic observations only; or intervention not indicated;
  - Moderate: requiring small, local or noninvasive treatment; restricted in age-appropriate instrumental activities of daily living (ADLs) (ADLs refer to cooking, shopping for clothing, using the telephone, managing money, etc.);
  - Severe: serious or medically significant but not immediately life-threatening; requiring hospitalization or prolongation of hospitalization; disabling; restricted in self-care ADLs (ADLs refer to bathing, dressing, undressing, eating, toileting, taking medications, etc., rather than bedridden).

In addition to the afore mentioned AE types, hypersensitivity reactions will be identified as TEAEs of special interest .

Adverse events will be summarized by default descriptive summary statistics for categorical variables for the SS by treatment group and overall as follows:

- An overview of TEAEs including the number and percentage of subjects with at least one of each mentioned TEAE type:
  - Any TEAE
    - Leading to death
    - Leading to discontinuation of study
    - Mild
    - Moderate
    - Severe
  - Any study treatment related TEAE
    - Mild
    - Moderate
    - Severe
  - Any serious TEAE
  - Any serious study treatment related TEAE
  - TEAEs of Special Interest
- The number and percentage of subjects reporting each TEAE will be summarized by SOC and PT for the following types of TEAEs:

  - TEAEs
  - TEAEs of Special Interest
  - TEAEs Leading to Death
  - TEAEs Leading to Discontinuation of Study
  - TEAEs by Maximum Severity
  - TEAEs by Relationship to Treatment
  - TEAEs by Relationship and Severity
  - Study Treatment related TEAEs
  - Serious TEAEs
  - Study Treatment Related Serious TEAEs
- The number and percentage of subjects who died will be summarized by the primary reason of death

In the above summaries, subjects with more than one TEAE within a particular SOC are counted only once for that SOC. Similarly, subjects with more than one TEAE within a particular PT are counted only once for that PT, respectively.

For summaries by maximum severity, subjects with multiple TEAEs within a particular SOC or PT will be counted under the category of their most severe TEAE within that SOC or PT. TEAEs with missing severity will be included (as Severe) in the overall count of subjects with TEAEs, and will be included in the counts of subjects with TEAEs within a SOC or PT as well.

Summaries by SOCs and PTs will be sorted by SOCs and PTs within SOC by descending order of incidence in TNM002 group first and then HTIG group. Where SOCs or preferred terms tie will be sorted alphabetically.

No statistical comparisons of AEs between treatment groups will be performed.

All AE data will be listed will be presented. The listing will present the relative start and stop day of the AE calculated relative to the dosing of study drug and will be presented for those subjects who received study treatment. If the AE is “Ongoing” it will be indicated as such in the listing and the relative stop day will not be calculated.

In addition, the following listings will be presented:

- Listing of Deaths
- Listing of TEAEs Leading to Discontinuation of Study
- Listing of Serious TEAEs
- Listing of TEAEs of Special Interest
- Listing of Treatment Related TEAEs
- Listing of Treatment Related Serious TEAEs

8.7.2. Laboratory Evaluations

Data for the following clinical chemistry, hematology, coagulation, urinalysis and pregnancy tests are required by the protocol as below:

Table 5 Laboratory Tests

Chemistry	Hematology	Urinalysis

• Total protein<br>• Albumin<br>• Sodium<br>• Potassium<br>• Chloride<br>• Calcium<br>• Glucose<br>• Aspartate aminotransferase (AST)<br>• Alanine aminotransferase (ALT)<br>• Total bilirubin<br>• Direct bilirubin<br>• Urea (blood urea nitrogen)<br>• Creatinine	• Red blood cell count<br>• Hemoglobin<br>• Platelet count<br>• White blood cell count<br>• Absolute neutrophil count<br>• Neutrophil percentage<br>• Absolute lymphocyte count<br>• Lymphocytes percentage<br>• Absolute monocyte count<br>• Hematocrit	• Protein<br>• Glucose<br>• Urobilinogen<br>• Urine occult blood<br>• Red blood cell<br>• White blood cell
	• **Coagulation**	**Others**
	• Prothrombin time<br>• Activated partial thromboplastin time	• Serum pregnancy test (β-hCG)

All laboratory data will be reported in SI units. Normal, abnormal (including clinically significant (CS) and not clinically significant (NCS)) will be used to evaluate the laboratory data. All quantitative laboratory test values at each assessed timepoints will be compared with the relevant reference range in SI units and categorized as:

- o Low: Below the lower limit of the reference range.
- o Normal: Within the reference range (upper and lower limits included).
- o High: Above the upper limit of the reference range.

For analysis purposes, values preceded by a “<” or a “>” sign (i.e. those below or above the limits of quantification) will be considered equal to the lower or upper limit of quantification, respectively.

Laboratory data will be summarized by default descriptive summary statistics for continuous and categorical variables for the SS by treatment group and overall as follows:

- Observed values and percentage change from baseline at each assessed timepoint for each standard continuous laboratory parameter;
- Shifts from baseline (normal vs. abnormal, NCS vs. abnormal, CS) to each post-baseline timepoint for each laboratory parameter.

Listings of all clinical laboratory data including derived percentage change from baseline will be provided for the SS. Within each listing, laboratory values outside the normal ranges will be flagged as either high or low for applicable laboratory assessments.

8.7.3. Vital Signs

Vital signs data will be summarized descriptively by scheduled time point at SoA defined in

the Protocol. The following vital sign parameters will be assessed:

- Systolic blood pressure (mmHg)

- Diastolic blood pressure (mmHg)
- Pulse rate (bpm)
- Respiration rate (breaths / min)
- Body temperature (ear,°C)

Normal, abnormal(including CS and NCS) will be used to evaluate the vital sign measurements.

The following will be presented for SS:

- Observed values and change from baseline at each scheduled timepoint for each standard vital sign parameter using default summary statistics for continuous variables.
- Shifts from baseline of vital sign (normal vs. abnormal, NCS vs. abnormal, CS) to each post-baseline timepoint.

A listing of all vital signs data including derived percentage change from baseline will be provided for the SS.

8.7.4. Electrocardiograms

The following electrocardiogram (ECG) assessments will be taken during the study:

- An overall investigator assessment classified as normal, abnormal, NCS / abnormal, CS
- Heart rate (bpm)
- PR interval (msec)
- QT interval (msec)
- QTc interval (msec)
- QRS interval(ms)

The maximum post-baseline QTc values will be classified in accordance with the ICH E14[1], Boundaries as presented in Table 6.

Table 6 QTcF / QTcB Interval ICH E14 Boundaries

QTc Interval	Criteria (msec)
Observed QTc interval	≤450 msec<br>>450 to ≤480 msec<br>>480 to ≤500 msec<br>>500 msec
Change from baseline in QTc interval	≤30<br>>30 to ≤60 msec<br>>60 msec

The ECG findings will be summarized by treatment group and overall for the SS as follows:

- Observed values and change from baseline at each scheduled timepoint for each standard ECG parameter using default summary statistics for continuous variables.
- Shifts from baseline of overall ECG assessment (normal vs. abnormal, NCS vs. abnormal, CS) to each post-baseline timepoint.
- A categorical summary of QTc classification according to ICH E14 boundaries will be provided using counts and percentages for baseline and maximum post-baseline value.

A listing of all ECG data including derived percentage change from baseline will be provided for the SS.

8.7.5. Physical Examination

Physical examinations will be performed according to SoA. The physical examination generally includes skin, lymph nodes, eyes, head and neck, chest, abdomen, spine, extremities, neurological, etc.

The physical examination data will be summarized descriptively at scheduled time points by treatment group separately and overall for the SS as follows.

- Shifts from baseline of physical examinations (normal vs. abnormal, NCS vs. abnormal, CS) to each post-baseline timepoint.

Physical examination findings (normal / abnormal) and details of abnormalities will be listed for each subject at each assessed timepoint.

8.7.6. Primary Analysis and Data Monitoring Committee (DMC)

The primary analysis will be performed after the last enrolled subject has completed the Day 28 visit. This analysis includes the final analysis of the primary and secondary efficacy endpoints and the interim analysis of the safety and other endpoints. The Sponsor is responsible for overseeing the whole process and ensuring that it is in line with the SAP. The Sponsor will clearly document the personnel who are informed of the unblinded

analysis result and the measures taken to maintain the double-blind status for the rest of the study. The investigators, study site staff and all personnels should be kept as blind will remain blinded throughout the study.

Data Monitoring Committee is not applicable for this study.

8.8. Pharmacokinetics Analysis

Pharmacokinetic analyses will be performed on the PKS. If appropriate, the analysis will be repeated in subgroups based on whether or not subjects received Tetanus vaccine as determined before randomization as well as in the two subgroups by age group (≤65 vs >65).

Samples collected for measurement of serum concentrations of investigational product will be sent to a third party bioassay laboratory and analyzed using a validated analytical method in compliance with applicable SOPs. The units of concentration and resulting PK parameters, with amount or concentration in the unit, will be presented as they are received from the analytical laboratory.

Drug concentration-time relationship will be summarized by nominal collection time points for serum samples using descriptive statistics. Individual and mean (including arithmetic and geometric) TNM002 concentration-time profiles will also be presented graphically, nominal collection time will be used in mean profile and actual collection time will be used in individual profile.

8.8.1. Presentation of Pharmacokinetic Concentration Data

- Descriptive statistics of number of subjects (n), arithmetic mean, arithmetic SD and arithmetic CV%, geometric mean, geometric SD and geometric CV%, median, minimum and maximum will be calculated in summaries.
- For the calculation of summary statistics, the BLQ values will be set to zero.
- If an embedded BLQ value is considered anomalous within the concentrationtime profile, this value will be excluded from the summary statistics.
- Where there is no result (NR), these will be set to missing.
- If there are less than three values in the data series, only the n, min and max will be presented. The other summary statistics will be denoted as not calculated (NC).
- If all the values are BLQ, then the arithmetic mean, arithmetic SD, median, min and max will be presented as zero, and the geometric mean, geometric SD and geometric CV% as well as the arithmetic CV% will be denoted as NC.
- If the value of the arithmetic mean or median is below the lower limit of quantification, these values will be presented as zero and the geometric mean,

geometric SD and geometric CV% as well as the arithmetic CV% will be denoted as NC.

- Out-of-window blood samples will be excluded from calculation of descriptive statistics.

8.9. Immunogenicity Analysis

Serum samples for immunogenicity tests will be collected from all subjects at pre-dose, and 7, 28 and 90 days after dosing.

Immunogenicity analysis will evaluate the result of titers and rate of ADA positivity in the subject. If confirming ADA positive, Neutralizing antibodies (NAbs) will be tested further. Meanwhile the effect of ADA on the PK and PD profile of the study drug will be evaluated if the data allow as well.

Based on the analysis results ADA positive rate (and the incidence of NAb, if applicable) at baseline and at different post-baseline timepoints will be summarized for the IAS. The number and percentage of NAb will also be further calculated for those positive subjects if data permit.

Descriptive statistics of number of subjects (n), arithmetic mean, arithmetic SD and arithmetic CV%, geometric mean, geometric SD and geometric CV%, median, minimum and maximum will be calculated for titers of ADA.

Immunogenicity data analysis will be performed on the IAS.

9. Changes in the Conduct of the Study or Planned Analysis

When defining the Immunogenicity Set (IAS), if the immunogenicity results are affected by protocol deviations, all or partial immunogenicity data of corresponding subjects will not be included in the IAS.

10. References

[1]Wang, Hongbo,Liu Si,Liu Cheng,Li Xueying,Xiong Hui,Wang, Chuanlin. *Meta-analysis on tetanus- antibody protection rate of healthy population born after 1978 in China*[C]//. Compilation of papers of the 2019 China Animal Injury Treatment Summit Forum,2019:86.DOI:10.26914/c.cnkihy.2019.014966.

[2]ICH. *Addendum on Estimands and Sensitivity Analysis in Clinical Trials*, Guideline E9(R1). Available at

https://database.ich.org/sites/default/files/E9-R1_Step4_Guideline_2019_1203.pdf

[3] Brown H and Prescott R. *Applied Mixed Models in Medicine*. John Wiley & sons (Chichester), 1999.

[4] ICH. *E14 Clinical Evaluation of QT/QTc Interval Prolongation and Proarrhythmic Potential for Non-Antiarrhythmic Drugs.* 12 May 2005. Available at

https://database.ich.org/sites/default/files/E14_Guideline.pdf

11. Appendices

Appendix 1: Document History

**Document Version, Status, Date**	**Summary / Reason for Changes**
Version 1.0, Final, 09JUL2023	Final version
Version 2.0, Final, 30AUG2023	Updated wording based on protocol 3.0 (16Jun2023). No planned analysis was changed from Version 1.0 to Version 2.0.
