# Supplementary material for: Recombinant monoclonal antibody siltartoxatug versus plasma-derived human tetanus immunoglobulin for tetanus: a randomized, double-blind, active-controlled, phase 3 trial
Source: Nat Med. 2025 Jul 8;31(8):2673–81. doi: 10.1038/s41591-025-03791-8 (PMC12353795; doi:10.1038/s41591-025-03791-8)
Supplement: Supplementary file 2 — Reporting Summary [file 41591_2025_3791_MOESM2_ESM.pdf]

nature portfolio

Corresponding author(s): Wanmei Wang MD PhD, Chuanlin Wang MD

Last updated by author(s): May 8, 2025

Reporting Summary

Nature Portfolio wishes to improve the reproducibility of the work that we publish. This form provides structure for consistency and transparency in reporting. For further information on Nature Portfolio policies, see our Editorial Policies and the Editorial Policy Checklist.

Statistics

For all statistical analyses, confirm that the following items are present in the figure legend, table legend, main text, or Methods section.

n/a	Confirmed	
☐	☒	The exact sample size (*n*) for each experimental group/condition, given as a discrete number and unit of measurement
☐	☒	A statement on whether measurements were taken from distinct samples or whether the same sample was measured repeatedly
☐	☒	The statistical test(s) used AND whether they are one- or two-sided<br>*Only common tests should be described solely by name; describe more complex techniques in the Methods section.*
☐	☒	A description of all covariates tested
☐	☒	A description of any assumptions or corrections, such as tests of normality and adjustment for multiple comparisons
☐	☒	A full description of the statistical parameters including central tendency (e.g. means) or other basic estimates (e.g. regression coefficient) AND variation (e.g. standard deviation) or associated estimates of uncertainty (e.g. confidence intervals)
☐	☒	For null hypothesis testing, the test statistic (e.g. *F*, *t*, *r*) with confidence intervals, effect sizes, degrees of freedom and *P* value noted<br>*Give P values as exact values whenever suitable.*
☒	☐	For Bayesian analysis, information on the choice of priors and Markov chain Monte Carlo settings
☒	☐	For hierarchical and complex designs, identification of the appropriate level for tests and full reporting of outcomes
☐	☒	Estimates of effect sizes (e.g. Cohen's *d*, Pearson's *r*), indicating how they were calculated

Our web collection on statistics for biologists contains articles on many of the points above.

Software and code

Policy information about availability of computer code

Data collection	Clinical data were electronically captured using Medidata Rave EDC 2022.3.0 - 2023.1.4
Data analysis	All statistical analysis were performed with SAS version 9.4

For manuscripts utilizing custom algorithms or software that are central to the research but not yet described in published literature, software must be made available to editors and reviewers. We strongly encourage code deposition in a community repository (e.g. GitHub). See the Nature Portfolio guidelines for submitting code & software for further information.

Data

Policy information about availability of data

All manuscripts must include a data availability statement. This statement should provide the following information, where applicable:

- Accession codes, unique identifiers, or web links for publicly available datasets
- A description of any restrictions on data availability
- For clinical datasets or third party data, please ensure that the statement adheres to our policy

The data supporting the findings of this trial are available within the article and its Supplementary Information. To ensure protection of participant privacy and proprietary information, de-identified individual participant data are available under restricted access. All requests for additional data sharing need to be reviewed by the lead study sites (Peking University People's Hospital and The First Affiliated Hospital of Guangzhou Medical University), as well as the sponsor (Zhuhai Trinomab Pharmaceutical Co., Ltd.) to assess any potential intellectual property or confidentiality concerns. Data requests should be sent to the corresponding

authors with detailed proposals for information use, and responses to such requests can be expected within 1 month. A signed data access agreement with the sponsors is required before data sharing.

Research involving human participants, their data, or biological material

Policy information about studies with human participants or human data. See also policy information about sex, gender (identity/presentation), and sexual orientation and race, ethnicity and racism.

Reporting on sex and gender	Both men and women aged ≥18 years could participate in the study. Participants' sex was determined based on self-report and official identification records before being documented in the electronic case report form. Among the enrolled participants, 58.7% were male, and 41.3% were female. Study objectives and endpoints were designed regardless of sex. Additionally, previous studies showed no clinically significant impact of sex on the antibody titer levels of siltartoxatug. Hence, no pre-specified subgroup analysis by sex was conducted.
Reporting on race, ethnicity, or other socially relevant groupings	The study was conducted solely in China and all enrolled participants were Chinese.
Population characteristics	Eligible participants were male or female at age of ≥ 18 years with unclean or contaminated wounds caused by various injuries, and who had incomplete or unknown tetanus immunization history, thus requiring tetanus passive immunization. The time from injury to study drug administration was required to be less than 24 hours.<br>Participants were excluded if they had a clean wound, needed HTIG 500 IU due to a very severe wound, had a prior vaccination history with ≥3 doses of tetanus toxoid-containing vaccine, were previously diagnosed with selective IgA deficiency, used immunoglobulins or blood products less than 6 months before enrollment, or had a history of anaphylaxis to study treatment components, human immunoglobulin products, or therapeutic mAbs.
Recruitment	Participants were recruited primarily from emergency departments in 28 hospitals across 14 provinces in China, between 22 December 2022 and 23 March 2023. The enrollment of a participant was determined by investigators based on the eligibility criteria pre-specified in the study protocol. The study adopted a double-blind design to furthest avoid potential biases.
Ethics oversight	The study protocol and its amendments were reviewed and approved by the independent ethics committee at each study site, as following: The First Affiliated Hospital of Guangzhou Medical University; Peking University First Hospital; the First Affiliated Hospital of Shenzhen University, Shenzhen Second People's Hospital; the Affiliated Nanhua Hospital, Hengyang Medical School, University of South China; Affiliated Hospital of Zunyi Medical University; The Third Affiliated Hospital of Guangzhou Medical University; The Second Affiliated Hospital of Nanchang Medical University; Tongji Hospital of Tongji Medical College, Huazhong University of Science and Technology; Liuzhou Worker's Hospital; The Second Affiliated Hospital of Anhui Medical University; Yuncheng Central Hospital; The First People's Hospital of Jinzhong; The Central Hospital of Wuhan, Tongji Medical College, Huazhong University of Science and Technology; The Second Xiangya Hospital of Central South University; Shanxi Bethune Hospital, Shanxi Academy of Medical Sciences, Third Hospital of Shanxi Medical University; PKUcare Luzhong Hospital; Zhejiang Provincial People's Hospital; Guangdong Provincial Hospital of Chinese Medicine; Renmin Hospital of Wuhan University; Nanfang Hospital, Southern Medical University; Beijing Tiantan Hospital, Capital Medical University; Hefei First People's Hospital; Hainan General Hospital (Hainan Affiliated Hospital of Hainan Medical University); The First Affiliated Hospital, Sun Yat-sen University; Guangzhou First People's Hospital; Zhongnan Hospital of Wuhan University; Peking University People's Hospital.

Note that full information on the approval of the study protocol must also be provided in the manuscript.

Field-specific reporting

Please select the one below that is the best fit for your research. If you are not sure, read the appropriate sections before making your selection.

☒ Life sciences ☐ Behavioural & social sciences ☐ Ecological, evolutionary & environmental sciences

For a reference copy of the document with all sections, see nature.com/documents/nr-reporting-summary-flat.pdf

Life sciences study design

All studies must disclose on these points even when the disclosure is negative.

Sample size	Sample size calculation was based on the following hypotheses and assumptions: (1) the lower limit of the 95% CI of the difference (siltartoxatug group - HTIG group) in the primary efficacy outcome would be > 0; (2) the proportion of participants with ΔTiters ≥0.01 IU/mL at 12 hours after receiving siltartoxatug and HTIG would be 91.8% and 55.9%, respectively, based on the results of phase 2 trial; (3) a 2:1 randomization ratio between the siltartoxatug and HTIG groups and a one-sided type I error of 0.025; (4) the dropout rate was expected to be 20%; (5) siltartoxatug required adequate exposure during clinical development to fully understand its safety. The planned sample size was 675 participants, ie., 450 in the siltartoxatug group and 225 in the HTIG group. This would provide a power of >99% to evaluate the above efficacy hypothesis, while ensuring adequate exposure of siltartoxatug to evaluate its safety.
Data exclusions	The efficacy analysis was conducted in the full analysis set, which included all randomized participants who received the study drugs. Additionally, a per-protocol set analysis was performed, encompassing all participants from the full analysis set who did not have important protocol deviations that could substantially impact the primary efficacy endpoint. Safety was analyzed in the safety set including all treated participants with at least 1 post-administration safety evaluation. Immunogenicity was analyzed in all randomized participants who received siltartoxatug and had available immunogenicity data for at least one post-administration time point.

Replication	This is a double-blind clinical trial, thus no experiments or measures were taken to replicate study findings.
Randomization	Participants were randomized at a 2:1 ratio to receive a single intramuscular gluteal injection of siltartoxatug 10 mg or HTIG 250 IU.Randomization was stratified based on whether or not the adsorbed tetanus vaccine was concomitantly administered, as determined at randomization. The proportion of participants receiving concomitant vaccination was set at approximately 10%, with the vaccine provided by four study sites qualified to administer it. The randomization sequence was generated by an independent randomization specialist. Investigators registered participants and assigned them according to the randomization sequence obtained from an Interactive Response Technology system.
Blinding	Participants, investigators and study site personnel (apart from the unblinded pharmacist and/or unblinded study nurses responsible for drug preparation and administration) remained blinded to all randomization assignments throughout the study.

Reporting for specific materials, systems and methods

We require information from authors about some types of materials, experimental systems and methods used in many studies. Here, indicate whether each material, system or method listed is relevant to your study. If you are not sure if a list item applies to your research, read the appropriate section before selecting a response.

Materials & experimental systems

n/a	Involved in the study
☐	☒ Antibodies
☒	☐ Eukaryotic cell lines
☒	☐ Palaeontology and archaeology
☒	☐ Animals and other organisms
☐	☒ Clinical data
☒	☐ Dual use research of concern
☒	☐ Plants

Methods

n/a	Involved in the study
☒	☐ ChIP-seq
☒	☐ Flow cytometry
☒	☐ MRI-based neuroimaging

Antibodies

Antibodies used	Siltartoxatug (formerly TNM002 or TT069) is provided by Zhuhai Trinomab Pharmaceutical Co., Ltd.<br>The positive control for this study, human tetanus immunoglobulin (HTIG), is manufactured by Hualan Biological Chongqing Co., Ltd.
Validation	Siltartoxatug is developed based on the fourth-generation antibody technology platform HitmAb®. See manufacturer's website for any detail: https://www.trinomab.com/en-us/<br>The concept validation for prophylaxis of tetanus by the antibody was published: Structural basis of tetanus toxin neutralization by native human monoclonal antibodies. Cell Rep. 35, 109070 (2021)

Clinical data

Policy information about clinical studies
All manuscripts should comply with the ICMJE guidelines for publication of clinical research and a completed CONSORT checklist must be included with all submissions.

Clinical trial registration	ClinicalTrials.gov, NCT05664750
Study protocol	The protocol is available in Supplementary Information of this paper.
Data collection	A total of 675 participants were recruited from 28 hospitals in China. The first and the last participants were enrolled in the study on 22 December 2022 and 23 March 2023, respectively. Safety data were collected throughout the course of the study, and other data were collected at specified study visits.
Outcomes	The primary efficacy endpoint was the increase of anti-tetanus neutralizing antibody titers from baseline (ΔTiters) at 12 hours post-administration, and the estimand for the primary endpoint was the proportions of participants with anti-tetanus neutralizing antibody ΔTiters ≥0.01 IU/mL at 12 hours post-administration, in all randomized participants who have received study drug.<br>Two quantitative determination methods were developed for the siltartoxatug and HTIG groups to measure the levels of anti-tetanus neutralizing antibodies. A quantitative ligand-binding electrochemiluminescence assay using a Meso Scale Discovery (QUICKPLEX SQ120MM – model 1300) platform was utilized to quantify anti-tetanus neutralizing monoclonal antibodies in the siltartoxatug group, referred to as the TNM002 PD bioanalytical method. A quantitative ligand-binding chemiluminescence assay, referred to as the HTIG PD bioanalytical method, was developed to detect anti-tetanus neutralizing polyclonal antibodies produced by participants in the HTIG group. The anti-tetanus neutralizing antibody ΔTiters in each group were calculated as the post-administration value at each time point minus the baseline value measured by the respective bioanalytical method. Furthermore, the HTIG PD bioanalytical method was also utilized to evaluate the levels of anti-tetanus antibodies (polyclonal antibodies) in the subgroup of participants who received the concomitant adsorbed tetanus vaccine, as well as to measure the baseline anti-tetanus antibody levels of all participants.<br>The secondary efficacy endpoint was the tetanus protection rate (1 – tetanus incidence) within 28 days post-administration. The investigators assessed potential occurrence of tetanus through patient interviews conducted at each study visit.

The tertiary efficacy endpoints included the anti-tetanus neutralizing antibody ΔTiters 3, 7, 28 and 90 days post-administration and the tetanus protection rate within 90 and 105 days post-administration.
Safety was assessed with (serious) adverse events, laboratory test, vital signs, physical examinations, and 12-lead electrocardiograms.

Plants

Seed stocks

Report on the source of all seed stocks or other plant material used. If applicable, state the seed stock centre and catalogue number. If plant specimens were collected from the field, describe the collection location, date and sampling procedures.

Novel plant genotypes

Describe the methods by which all novel plant genotypes were produced. This includes those generated by transgenic approaches, gene editing, chemical/radiation-based mutagenesis and hybridization. For transgenic lines, describe the transformation method, the number of independent lines analyzed and the generation upon which experiments were performed. For gene-edited lines, describe the editor used, the endogenous sequence targeted for editing, the targeting guide RNA sequence (if applicable) and how the editor was applied.

Authentication

Describe any authentication procedures for each seed stock used or novel genotype generated. Describe any experiments used to assess the effect of a mutation and, where applicable, how potential secondary effects (e.g. second site T-DNA insertions, mosiacism, off-target gene editing) were examined.
